# New Benzimidazole 3′-Deoxynucleosides: Synthesis and Antiherpes Virus Properties

**DOI:** 10.3390/biom15070922

**Published:** 2025-06-23

**Authors:** Aleksandra O. Arnautova, Irina A. Aleksakhina, Ekaterina A. Zorina, Maria Ya. Berzina, Ilya V. Fateev, Barbara Z. Eletskaya, Konstantin V. Antonov, Olga S. Smirnova, Alexander S. Paramonov, Alexey L. Kayushin, Valeria L. Andronova, Georgii A. Galegov, Maria A. Kostromina, Evgeny A. Zayats, Inna L. Karpenko, Svetlana K. Kotovskaya, Valery N. Charushin, Roman S. Esipov, Anatoly I. Miroshnikov, Irina D. Konstantinova

**Affiliations:** 1 Shemyakin-Ovchinnikov Institute of Bioorganic Chemistry, Russian Academy of Sciences, Miklukho-Maklaya St. 16/10, 119991 Moscow, Russia; aleksahinaia@gmail.com (I.A.A.); katuzorina@mail.ru (E.A.Z.); berzina_maria@mail.ru (M.Y.B.); ifateev@gmail.com (I.V.F.); fraubarusya@gmail.com (B.Z.E.); antonov.kant@yandex.ru (K.V.A.); gescheites@gmail.com (O.S.S.); apar@nmr.ru (A.S.P.); kayushin.alexej@yandex.ru (A.L.K.); kostromasha@mail.ru (M.A.K.); eaz96post@gmail.com (E.A.Z.); esipov@ibch.ru (R.S.E.); aiv@mail.ibch.ru (A.I.M.); 2 Engelhardt Institute of Molecular Biology, Russian Academy of Sciences, Vavilova St. 32, 119991 Moscow, Russia; ilkzkil@gmail.com; 3 Ivanovsky Institute of Virology, Gamaleya National Research Center for Epidemiology, Ministry of Healthcare of the Russian Federation, Gamaleya St. 18, 123098 Moscow, Russia; andronova.vl@yandex.ru (V.L.A.); kid196892@gmail.com (G.A.G.); 4 Postovsky Institute of Organic Synthesis, The Ural Branch of the Russian Academy of Sciences, S. Kovalevskaya/Academicheskaya St. 22/20, 620041 Ekaterinburg, Russia; sk-kotovskaya-665@yandex.ru (S.K.K.); charushin@ios.uran.ru (V.N.C.); 5 Department of Organic and Biomolecular Chemistry, Yeltsin Ural Federal University, N., Mira St. 19, 620002 Ekaterinburg, Russia

**Keywords:** nucleosides, fluorinated benzimidazoles, transglycosylation reaction, purine nucleoside phosphorylase, herpes simplex virus, 3′-deoxyribonucleosides

## Abstract

A series of new 3′-deoxyribosides of substituted benzimidazoles was obtained by the chemo-enzymatic method using genetically engineered *E. coli* purine nucleoside phosphorylase (PNP). In the case of asymmetrically substituted benzimidazole derivatives, a mixture of N1- and N3-regioisomers was formed (confirmed by NMR). The antiviral activity of the obtained compounds against herpes simplex virus 1 of reference strain L2 and a strain deeply resistant to acyclovir in Vero E6 cell culture was studied. 4,6-Difluoro-1-(β-D-3′-deoxyribofuranosyl)benzimidazole (IC_50_ = 250.92 µM, SI = 12.00) and 4,5,6-trifluoro-1-(β-D-3′-deoxyribofuranosyl)benzimidazole (IC_50_ = 249.96 µM, SI = 16.00) showed significant selective activity against both viral models in comparison to ribavirin (IC_50_ = 511.88 µM, SI > 8.00).

## 1. Introduction

The prevalence of infections caused by the herpes simplex virus 1 (HSV-1) in the worldwide adult population, according to WHO, reaches 67% [1]. After primary infection, HSV establishes a lifelong latent infection in peripheral sensory ganglia and is periodically activated, causing productive lytic infections clinically manifested by various cutaneous and mucosal pathologies [2]. In immune naïve or immunocompromised individuals, HSV can cause serious diseases, including blindness, life-threatening systemic infections, and encephalitis [3]. Basic anti-HSV drugs are nucleoside analogue-based medicaments (acyclovir, penciclovir) or their metabolic precursors (valtrex, famciclovir). After activation (triphosphorylation), they directly target viral DNA polymerase and inhibit viral DNA replication [4]. It is known that resistance can occur to drugs of this group, mainly in the group of immunocompromised patients, and the efficiency of therapy decreases [5]. Therefore, the search for new antiviral agents remains an urgent issue of medicinal chemistry.

The benzimidazole nucleus is an outstanding pharmacophore in the field of medicinal chemistry [6,7,8]. Drug molecules containing benzimidazole moiety exhibit versatile biological activities, like antiviral, antibacterial, antifungal, antitumor, anticancer, antihypertension, and antiparasitic effects and often immunosuppressive properties [9].

Benzimidazole nucleosides have selective antiviral activity against human herpes simplex viruses 1 and 2 (HSV-1 and HSV-2), as well as human herpes virus 6, human cytomegalovirus (HCMV), and Epstein-Barr virus (EBV) [10,11]. They are characterized by low systemic toxicity [12,13].

**Figure 1:**
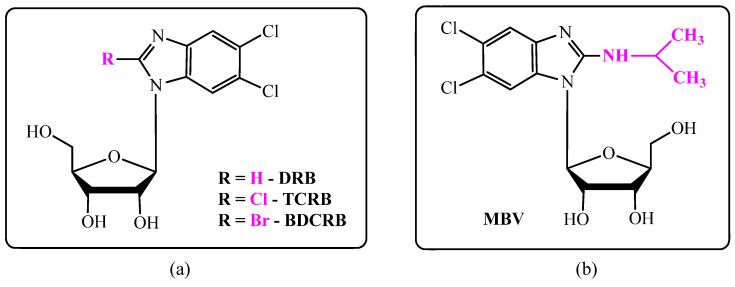
Structures of typical halogenated benzimidazole nucleosides. (**a**) benzimidazole nucleosides with antiviral activity; (**b**) structure of Maribavir.

In 1954, a series of halogenated benzimidazole nucleosides (Figure 1a) was obtained, and their antiviral activity was evaluated. Among the compounds obtained, 5,6-dichloro-1-(β-D-ribofuranosyl)benzimidazole (DRB) was found to be the most active against influenza A and B viruses [14] and other RNA- and DNA-based viruses (e.g., cowpox virus) and inactive against HCMV and HSV-1 (IC_50_ = 42 and 30 µM, respectively) [15,16,17]. In addition, DRB is characterized by high cytotoxicity, and it cannot be used as an antiviral drug [18]. The 2-substituted DRB analogs, 2,5,6-trichloro-1-(β-D-ribofuranosyl)benzimidazole (TCRB) and 2-bromo-5,6-dichloro-1-(β-D-ribofuranosyl)benzimidazole (BDCRB) (Figure 1a), were minimally active against HSV-1 but highly selective against HCMV at non-cytotoxic concentrations; moreover, BDCRB was more than four times more active than TCRB [19].

Among the modern derivatives of the benzimidazole family, only 2-isopropylamino-5,6-dichloro-1-(β-L-ribofuranosyl)benzimidazole—Maribavir (MBV) (Figure 1b) with specific activity against HCMV is currently used in clinical practice [20]. Maribavir (Livtencity^®^, Takeda, Tokyo, Japan) was approved for medical use in the United States in November 2021 [21] and in the European Union in November 2022 [22] for treating adults and pediatric patients (12 years old and older and weighing at least 35 kg) with post-transplant CMV infection/disease that does not respond (with or without genetic mutations that cause resistance) to available antiviral treatment for CMV. The U.S. Food and Drug Administration (FDA) considers it to be a first-in-class medication.

Ribose residue modifications of DRB did not result in enhanced antiviral activity and reduced cytotoxicity [23,24]. 2′-Deoxy-β-D-ribo forms of TCRB and BDCRB possess higher cytotoxicity and less pronounced activity against HSV-1 and HCMV (compared to ganciclovir) and are comparable in activity to foscarnet [25].

Benzimidazole nucleosides structurally mimic purine nucleosides and exhibit a wide range of biological activities by interacting with DNA, RNA, and/or proteins [26]. Notably, the biological activity profile of benzimidazole nucleosides can be regulated by changing the substituents in the benzimidazole ring and/or carbohydrate residue. Obviously, even small structural modifications of the benzimidazole fragment lead to a significant change in biological activity [27]. It is known that the introduction of fluorine atoms into the molecule of a heterocyclic compound leads to a significant change in the pharmacokinetic and pharmacodynamic properties of the molecule [28].

The cytotoxicity and biological activity of nucleosides can be influenced not only by the introduction of substituents into the heterocyclic base, but also by the replacement of natural carbohydrate residues (D-ribose or 2-deoxy-D-ribose) with non-natural ones. It has been shown that synthetic 2′-deoxy-2′-fluoroarabinosides of substituted benzimidazole exhibit selective anti-herpetic activity in vitro [29].

The aim of the present study was to develop the enzymatic synthesis of new 3′-deoxyribosides of substituted fluorinated benzimidazoles and to investigate the antiviral activity of the obtained compounds against HSV-1 reference strain L2 (HSV-1/L_2_) and a strain profoundly resistant to acyclovir HSV-1/L_2_/R^ACV^), in vitro. The motivation for the choice of bases and their structures are given in the Results and Discussion. One of the main challenges we faced was to reduce cytotoxicity while retaining antiviral activity.

## 2. Materials and Methods

### 2.1. General Procedures

Unless otherwise noted, materials were obtained from commercial suppliers and used without any purification. 3′-Deoxyinosine was obtained in accordance with [30]. Substituted benzimidazoles were obtained by the IOS of the Ural Branch of the Russian Academy of Sciences (Yekaterinburg). Recombinant *E. coli* PNP (protein concentration by the Bradford method—28 mg/mL, activity—50 U/mg of the protein) was obtained in accordance with the method previously reported [31]. Herpes simplex virus 1 strain L2 was obtained from the State′s collection of viruses at FSBI «N. F. Gamaleya National Research Centre for Epidemiology and Microbiology, Russian Ministry of Health, Russia» (“D. I. Ivanovsky Institute of Virology” subdivision).

Column chromatography was performed on Silica gel 100 C18-reversed-phase (Fluka, Buchs, Switzerland). High-performance liquid chromatography (HPLC) was performed on the Waters system (Waters 1525, Waters 2489, Breeze 2) using column Ascentis Express C18, 3.0 × 75 mm, 2.7 µm, flow rate 0.5 mL/min, detection at 280 nm.

HPLC Method 1: Eluent A: 0.1% TFA in water, eluent B: 0.1% TFA in acetonitrile–water, 70:30 (*v*/*v*). Gradient: 0–30% B, 20 min. HPLC Method 2: Eluent A: 0.1% TFA in water, eluent B: 0.1% TFA in acetonitrile–water, 70:30 (*v*/*v*). Gradient: 30–100% B, 20 min. Preparative HPLC was performed using column MZ-PREPARATIVE, 250 × 20 mm, PerfectSil Target, ODS-3, 5 µm, flow rate 4 mL/min, detection at 280 nm.

NMR spectra were recorded on Bruker Avance II 700 spectrometers (Bruker BioSpin, Rheinstetten, Germany) in DMSO-d6 at 303 K. The operating frequency for ^1^H-NMR—700 MHz, for ^13^C—176 MHz, and for ^15^N—71 MHz. Chemical shifts in ppm (δ) were measured relative to the residual solvent signals as internal standards (2.50). Coupling constants (J) were measured in Hz. NMR spectra ^1^H-NMR (700 MHz, DMSO-d6), ^13^C (176 MHz, DMSO-d6), ^15^N (71 MHz, DMSO-d6) are presented in Supplementary Materials (Figures S17–S32). Liquid chromatography mass spectrometry was performed on Agilent 6210 TOF LC/MS system (Agilent Technologies Inc., Santa Clara, CA, USA).

### 2.2. Enzymatic Reactions

Each reaction mixture (1 mL, pH 6.0–11.0) contained 1–3 mM tested heterocyclic base, 1–3 mM 3′-deoxyinosine, 2–10 mM potassium phosphate buffer, and *E. coli* PNP (0.7–21 units). The reaction mixtures were incubated at 50 °C. Substrate and product quantities were determined using HPLC.

### 2.3. Nucleosides Synthesis

#### 2.3.1. 1-(β-D-3′-Deoxyribofuranosyl)benzimidazole (**9**)

Benzimidazole **1** (271 mg, 2.29 mmol) and 3′-deoxyinosine (64 mg, 0.25 mmol) were dissolved in 200 mL of 10 mM potassium phosphate buffer, pH 7.0; 4200 units of PNP were added and incubated at 50 °C for 20 days. The progress of the reaction was monitored by HPLC (Method 1). The reaction was stopped when the conversion reached 54% by adding 20 mL of ethanol.

The solution was concentrated to 5 mL, product **9** was isolated by reversed-phase chromatography (column 15 × 170 mm) in a concentration gradient of methanol in water (0–30%, 300 mL of each buffer). Preparative HPLC (eluent A–0.1% aqueous TFA, eluent B–70% acetonitrile in 0.1% aqueous TFA, concentration gradient—0–30% B, 4 h) was used for final purification. Fractions containing compound **9** were combined, neutralized with aqueous ammonia solution, concentrated, and desalted by reversed-phase chromatography (10 × 100 mm) in a gradient of methanol in water (0–50%, 140 mL of each buffer). The fractions containing the target product **9** were evaporated and lyophilized.

Yield: 16 mg (0.07 mmol, 27%), purity: 99% (HPLC data).

ESI/MS [M − H]^−^: calculated for C_12_H_13_N_2_O_3_ 233.0932, found 233.1180.

#### 2.3.2. 5,6-Difluoro-1-(β-D-3′-deoxyribofuranosyl)benzimidazole (**10**)

5,6-Difluorobenzimidazole **2** (183 mg, 1.18 mmol) and 3′-deoxyinosine (100 mg, 0.39 mmol) were dissolved in 100 mL of 10 mM potassium phosphate buffer, pH 7.0; 1400 units of PNP were added and incubated at 50 °C for 40 days. The progress of the reaction was monitored by HPLC (Method 1). The reaction was stopped when 93% conversion was reached by adding 20 mL of ethanol.

The reaction mixture was concentrated to 5 mL, product **10** was isolated by reversed-phase chromatography (15 × 170 mm) in a concentration gradient of methanol in water (0–30%, 150 mL of each buffer). The fractions contained product **10** mixed with 5,6-difluoro-benzimidazole **2**. The mixture was concentrated to a minimum volume, and product **10** was purified by preparative HPLC (eluent A—0.1% aqueous TFA, eluent B—40% acetonitrile in 0.1% aqueous TFA, concentration gradient 10—100% B, 3 h). Fractions containing the target compound **10** were combined, neutralized with aqueous ammonia solution, concentrated, and desalted by reversed-phase chromatography (15 × 170 mm) in a gradient of methanol in water (0—60%, 100 mL of each buffer). The fractions containing the target product **10** were combined, evaporated, and lyophilized.

Yield: 33 mg (0.07 mmol, 32%), purity: 99% (HPLC data).

ESI/MS [M + H]^+^: calculated for C_12_H_13_F_2_N_2_O_3_ 271.0816, found 271.0888.

#### 2.3.3. 4,6-Difluoro-1-(β-D-3′-deoxyribofuranosyl)benzimidazole (**11**)

4,6-Difluorobenzimidazole **3** (0.025 g, 0.16 mmol) and 3′-deoxyinosine (0.205 g, 0.81 mmol) were dissolved in 190 mL of 6 mM potassium phosphate buffer, pH 7.0; 1400 units of PNP were added and incubated at 50 °C for 40 days. The progress of the reaction was monitored by HPLC (Methods 1, 2). The reaction was stopped when the conversion reached 73% by adding 20 mL of ethanol.

The solution was concentrated to 5 mL, and products **11** were isolated by reversed-phase chromatography (20 × 190 mm) in a concentration gradient of methanol in water (0–40%, 300 mL each). Fractions containing compounds **11** were combined, concentrated, and separated by preparative HPLC. Conditions: eluent A—0.1% aqueous TFA, eluent B—70% acetonitrile in 0.1% aqueous TFA, concentration gradient 40–100% B, 4 h. Fractions containing compounds **11a** and **11b** were neutralized with aqueous ammonia solution, concentrated, and desalted by reversed-phase chromatography (15 × 140 mm) in a gradient of methanol in water (0–40%, 150 mL of each buffer). Fractions containing products **11a** and **11b** were evaporated and lyophilized.

Yield of nucleoside **11a**—4 mg (0.014 mmol, 14%), **11b**—3 mg (0.011 mmol, 11%), purity: 95% and 90%, respectively (HPLC data).

ESI/MS [M + H]^+^: calculated for C_12_H_13_F_2_N_2_O_3_ 271.0816, found 271.0848.

#### 2.3.4. 4,5,6-Trifluoro-1-(β-D-3′-deoxyribofuranosyl)benzimidazole (12)

4,5,6-Trifluorobenzimidazole **4** (110 mg, 0.64 mmol) and 3′-deoxyinosine (32.37 mg, 0.13 mmol) were dissolved in 100 mL of 2 mM potassium phosphate buffer, pH 7.0; 2800 units of PNP were added and incubated at 50 °C for 40 days. The progress of the reaction was monitored by HPLC (Method 1). The reaction was stopped when the conversion reached 58% by adding 20 mL of ethanol.

The reaction mixture was concentrated to 5 mL, products **12** were isolated by reversed-phase chromatography (15 × 170 mm) in a concentration gradient of methanol in water (0–30%, 150 mL of each buffer). The fractions contained a mixture of products and 4,5,6-trifluoro-benzimidazole. The mixture was concentrated to a minimum volume, and products **12** were separated by preparative HPLC (eluent A—0.1% aqueous TFA, eluent B—40% acetonitrile in 0.1% aqueous TFA, concentration gradient 10—100% B, 3 h). Fractions containing the target nucleoside **12a** and minor nucleoside **12b** were neutralized with aqueous ammonia solution, concentrated, and desalted by reversed-phase chromatography (15 × 170) in a gradient of methanol in water (0–60%, 100 mL of each buffer). Fractions containing products **12a** and **12b** were evaporated and lyophilized.

Yield of nucleoside **12a**—11 mg (0.038 mmol, 30%), **12b**—6 mg (0.021 mmol, 16%), purity: 98% and 99%, respectively (HPLC data).

ESI/MS [M + H]^+^: calculated for C_12_H_12_F_3_N_2_O_3_ 289.0722, found 289.0796.

#### 2.3.5. 4,6-Difluoro-5-metoxy-1-(β-D-3′-deoxyribofuranosyl)benzimidazole (**13**)

4,6-Difluoro-5-methoxybenzimidazole **5** (230 mg, 0.12 mmol) and 3′-deoxyinosine (215 mg, 0.85 mmol) were dissolved in 100 mL of 6 mM potassium phosphate buffer, pH 7.0; 2100 units of PNP were added and incubated at 50 °C for 48 h. The progress of the reaction was monitored by HPLC (Method 1). The reaction was stopped when 86% conversion was reached by adding 50 mL of ethanol.

The solution was concentrated to a minimum volume, and product **13** was isolated by reversed-phase chromatography (20 × 200 mm) in a concentration gradient of methanol in water (0–40%, 250 mL each). The fractions containing target product **13** were evaporated and lyophilized.

Yield: 20 mg (0.067 mmol, 58%), purity: 95% (HPLC data).

ESI/MS [M − H]^−^: calculated for C_13_H_13_F_2_N_2_O_4_ 299.0922, found 299.0954.

#### 2.3.6. 2-Amino-5,6-difluoro-1-(β-D-3′-deoxyribofuranosyl)benzimidazole (**16**)

2-Amino-5,6-difluorobenzimidazole **8** (601 mg, 3.5 mmol) and 3′deoxyinosine (100 mg, 0.4 mmol) were dissolved in 200 mL of 9 mM potassium phosphate buffer, pH 7.0; and 3500 units of PNP were added. The reaction mixture was incubated at 50 °C for 30 days. The progress of the reaction was monitored by HPLC (Method 1). The reaction was stopped when the conversion value reached 62% by adding 50 mL of ethanol.

The solution was concentrated to a minimum volume, and product **16** was isolated by reversed-phase chromatography (20 × 190 mm) in a concentration gradient of methanol in water (0–40%, 400 mL each). Fractions containing a mixture of nucleoside **16** and the initial 2-amino-5,6-difluorobenzimidazole were concentrated and isolated by preparative HPLC. Chromatography conditions: eluent A—water, eluent B—50% aqueous methanol, concentration gradient 40–100% B, 4 h. Fractions containing the target product were evaporated and lyophilized.

Yield: 8.5 mg (0.03 mmol, 8%), purity: 97% (HPLC data).

ESI/MS [M + H]^+^: calculated for C_12_H_14_F_2_N_3_O_3_ 286.0925, found 286.0951.

### 2.4. Antiviral Activity and Cytotoxicity

The research was carried out for the foundation of the Ivanovsky Institute of Virology (Gamaleya National Research Center for Epidemiology, Ministry of Healthcare of the Russian Federation).

Vero 6 cells for determining cytotoxicity and antiviral activity were cultured according to the method described in [29].

We obtained an acyclovir-resistant strain (HSV-1/L_2_/R^ACV^) by serial passaging of HSV-1/L_2_ in the presence of acyclovir followed by cloning. Amino acid substitutions in the thymidine kinase (TK) of HSV-1/L_2_/R^ACV^ (W88R, R220H) have been identified that may be associated with changes in drug sensitivity (localized in conserved regions of the enzyme or described in the literature, respectively). Phenotypically, the virus was characterized as TK-negative. Ribavirin, a nucleoside analogue and well-known commercial broad-spectrum antiviral drug, was chosen as a comparison drug.

In 96-well plastic plates with the generated cell monolayer, serial dilutions of the compounds were prepared at a multiplicity of infection (MOI) of 1:2 with the maintenance medium (Eagle′s medium and medium 199, mixed in a ratio 1:1). Vero E6 cells were then infected with the infection material at an MOI of 0.01 PFU/cell; incubation was performed at 37 °C in an atmosphere of 5% CO_2_. Cytotoxicity was determined quantitatively by the trypan blue exclusion method and expressed as CD_50_: the concentration of the drug at which 50% of cells die after 72 h. The definition of the antiherpes activity in vitro of compounds **1**–**13**, **16** was produced by the method of cytopathic effect (CPE) inhibition assay. Three controls were used: (1) Toxicity control: Uninfected cells were incubated in the presence of the drug in the same concentration range as in the experiment; (2) Virus control: Cells were infected under conditions described above, but the maintenance medium did not contain the drug; and (3) Cell control: Uninfected cell cultures were incubated in the maintenance medium, which contained no drug. The duration of incubation was 48 h and 72 h for MOI 0.01 PFU/mL, respectively. In the control, viral CPE was developed to 95–100%. An antiviral activity was assessed by determining ID_50_ and ID_95_ (concentrations of compounds that inhibit the development of virus-induced CPE as compared with the control by 50% and almost completely). Standard deviation was not more than 5%. The results of three independent experiments are shown in Table S1 (Supplementary Materials).

## 3. Results and Discussion

### 3.1. Enzymatic Synthesis

Currently, enzymatic methods for the preparation of benzimidazole nucleosides using bacterial N-deoxyribosyltransferases (NDTs) [32,33] and nucleoside phosphorylases (NPs) are being actively developed. NDTs are known to show strict specificity to 2′-deoxynucleosides [34], whereas NPs show relatively low substrate specificity and find application in the synthesis of both deoxy- and ribonucleosides. Moreover, benzimidazole bases are good substrates for PNPs because of their high acceptor capacity due to their affinity for the PNP active site [35].

PNP is present in all tissues and cells of the body and performs a key function in the metabolism of purine bases, catalyzing phosphorolysis leading to the degradation of purine nucleosides. Genetically engineered PNP of *E. coli* is a hexameric protein with a molecular mass of 162 kDa [36]. The structure of the active site of the enzyme determines its broad substrate specificity with respect to many compounds of purine nucleosides [37], which allows the synthesis of many new modified nucleosides with interesting biological properties.

**Figure 2:**
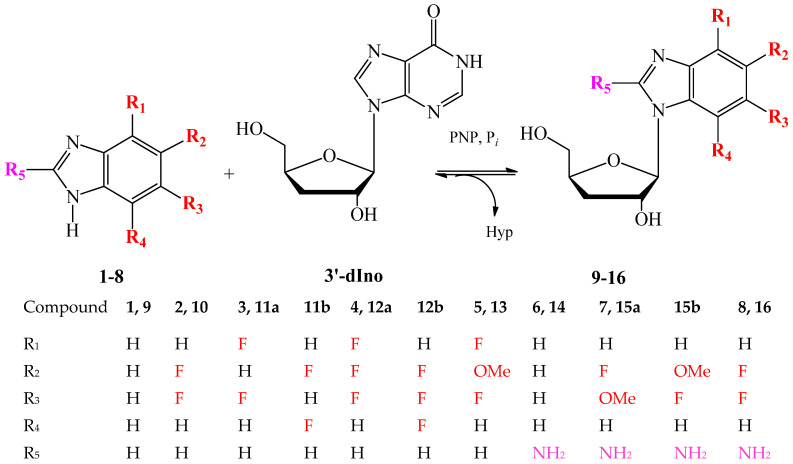
Scheme for the synthesis of 3′-deoxyribosides of benzimidazole and its derivatives.

A series of modified 3′-deoxyribosides of benzimidazole and its fluorinated analogs were synthesized by a transglycosylation reaction catalyzed by recombinant PNP *E.coli* according to the scheme shown in Figure 2. PNP in the presence of an inorganic phosphate catalyzes the reversible phosphorolysis of 3′-dIno, resulting in cleavage of the glycosidic C-N bond, formation of a free hypoxantine and α-D-pentofuranosyl-1-phosphate (pentose-1-phosphate), and transfer of pentose-1-phosphate to another heterocyclic base.

The enzymatic approach does not require the introduction of protecting groups either in the heterocyclic base or in the carbohydrate part of the nucleoside molecule and is characterized by the stereo- and relative regio-selectivity of the process.

The choice of bases **2**–**8** is due to the results obtained by screening the antiviral activity of previously synthesized 2′-deoxy-2′-fluoroarabinosides of substituted benzimidazole [29], as well as the results of [38]. In the latter study, it was shown that 2-amino-5,6-difluorobenzimidazole riboside exhibited a selective antiviral activity (selectivity index > 32) against a wild strain of the herpes simplex virus 1, as well as towards virus strains that are resistant to acyclovir, cidofovir, and foscarnet. Therefore, we were interested in whether 3-deoxyribosides of various benzimidazoles would exhibit antiviral activity. The choice of base 7 (2-amino-5-fluoro-6-methoxybenzimidazole) was based on the data of its antiviral activity and the possibility to modify it by obtaining its nucleosides.

Optimization of the synthesis conditions for each nucleoside was carried out according to the following scheme: (i) qualitative determination of the substrate specificity of the enzyme towards the base; (ii) determination of the optimal substrate ratio for the transglycosylation reaction; and (iii) determination of the optimal enzyme concentration.

**Figure 3:**
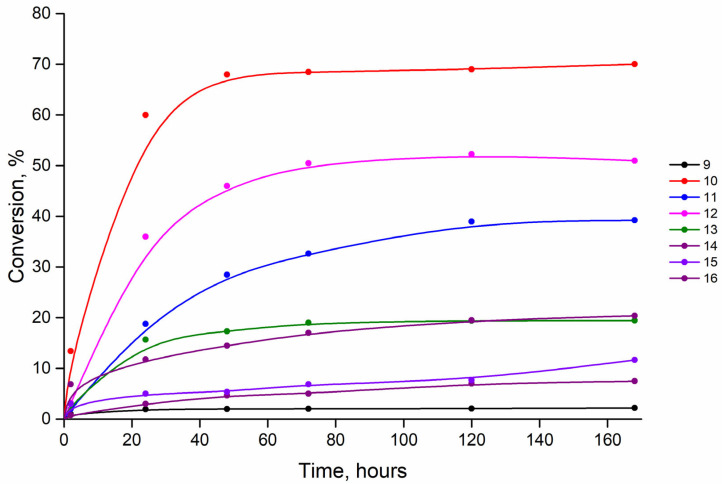
Conversion of 3′-dIno to nucleosides **9**–**16**. Conditions: the ratio of bases **1**–**8** to 3′-dIno—3:1 (by moles); 50 °C, 2 mM potassium phosphate (pH 7.0), PNP concentration 7 units/mL.

In the first step, we studied the substrate-specific properties of PNP towards benzimidazole **1**, chosen as a reference base, and substituted bases **2**–**8**. 3′-Deoxyinosine (3′-dIno), which we synthesized earlier [30], was chosen as the carbohydrate residue donor. It is known that the substrate specificity testing of PNP is usually carried out at a two- to three-fold excess (by moles) of the carbohydrate fragment donor with respect to the modified base. But in our case, the technology of 3′-dIno synthesis was more complicated than that of benzimidazole bases. Therefore, we tested the substrate specificity of PNP using the three-fold excess of benzimidazole base to 3′-dIno. The results are summarized in Figure 3.

According to the dynamics of the accumulation of products **9**–**16** (Figure 3), it is obvious from the presented data that the conversion of bases into nucleosides **9**, **13**–**16** is low under these conditions, which required further optimization of the synthesis. At the same time, the conversion values of bases **2** and **4** to nucleosides **10** and **12** indicate that they are good substrates for PNP. The results of the experiments on the selection of substrate ratios and enzyme concentrations are presented in Table 1 (Figures S1–16).

The data from experiments on the selection of substrate ratios show that the reactions with bases **3** and **5** with the formation of products **11** and **13**, respectively, are characterized by an increase in conversion at an excess of the 3-deoxyribose donor, 3′-dIno. In other reactions, the conversion value increases with the increasing amount of base in the reaction mixture. The *E. coli* PNP concentration in the test reactions ranged from 0.7 to 21 units/mL. In all cases, the reaction rate increased expectedly with increasing amounts of PNP. In reactions with bases **1** to **5**, a PNP concentration of 7 units/mL was sufficient to achieve high conversion. Changing the reaction conditions with 2-aminobenzimidazole **6** had almost no effect on the conversion to product **14** (Figures S11 and S12), while with base **7,** it increased the conversion to product **15** from 11 to 28% (Figures S13 and S14).

One of the reasons for low conversion can be poor solubility of substituted benzimidazoles in water.

To improve the solubility of the bases, 5% DMSO was added to the reaction mixtures.

**Figure 4:**
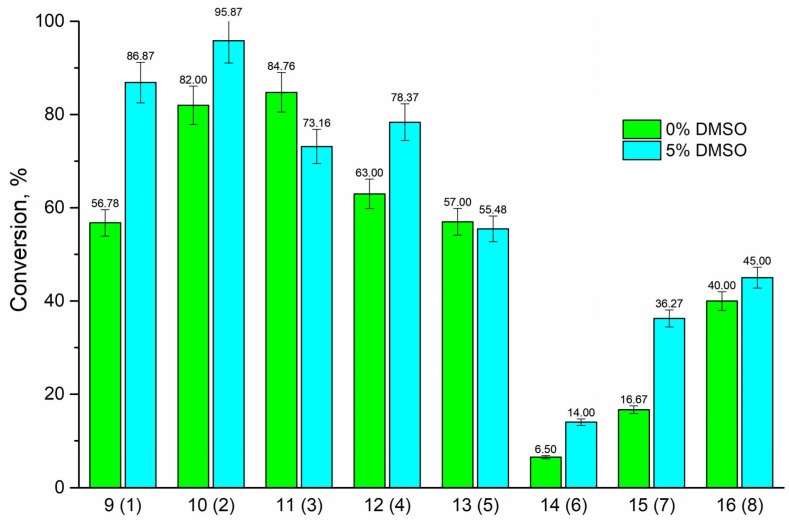
Effect of 5% DMSO on the conversion of starting substrates to nucleosides **9**–**16**. Conditions: reaction volume 1 mL, base ratios **1**–**8** to 3′-dIno, amount of PNP- are given in Table 1, 50 °C, 2–10 mM potassium phosphate (pH 7.0), reaction time—168 h.

According to the data obtained (Figure 4), it is obvious that the addition of 5% DMSO to the reaction mixtures with bases **1**, **2**, **4**, **7**, and **8** led to an increase in conversion: with 2-aminobenzimidazole **6**—a slight change in the conversion value from 6.5% to 14%, with base **3**—a decrease in conversion. It should be noted that the increase in conversion is characteristic only for those reactions in which the base was taken in excess. Obviously, when the solubility of bases **1**, **2**, **4**, **7**, and **8** is improved in the presence of DMSO, the bases’ molar ratios in the reaction mixture increase, which favorably affects the formation of reaction products. And the opposite effect is observed in the case of reactions with bases **3** and **5**, where 3′-dIno was taken in excess.

The addition of DMSO to the solution can significantly alter the enzyme activity, substrate specificity, and stability [39,40,41,42]. The presence of an organic solvent may affect the substrate’s solubility, the strength of protein–ligand hydrophobic interactions, and the structure of the active site of the enzyme. According to our previous experience (unpublished research), the presence of DMSO can significantly alter *E. coli* PNP activity towards unnatural substrates, decreasing or increasing in different cases. Moreover, it should be noted that the catalytic cycle of *E. coli* PNP includes conformational changes [43]. The presence of DMSO can theoretically influence the transition between the open and the closed conformations, which in turn could affect the substrate specificity of the enzyme.

After optimization of the conditions of the transglycosylation reactions, nucleosides **9**–**13**, and **16** were synthesized (experimental data are shown in Table 2).

The substituted benzimidazole nucleosides **9**–**13** and **16** were isolated from the reaction mixtures by reversed-phase chromatography with a purity of 90–99% (HPLC data) and characterized by mass spectrometry and NMR spectroscopy (Figures S17–S41).

In the case of asymmetrically substituted benzimidazole derivatives **3** and **4**, N1- and N3-nucleosides were formed. The formation of a mixture of regioisomers as a result of the transglycosylation reaction using *E. coli* PNP was shown for the first time earlier in [44].

**Figure 5:**
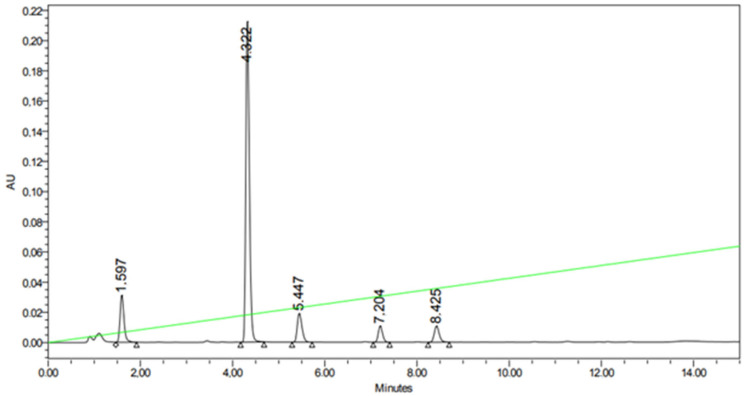
HPLC profile of the reaction mixture with substituted benzimidazole **3**.

Figure 5 shows the HPLC profile of the reaction mixture with substituted benzimidazole **3**. The peaks with RT 7.204 and 8.425 min correspond to compounds **11b** and **11a**.

Both compounds **11** have the same [M + H]^+^ values of 271.0888, hence they are isomers.

**Figure 6:**
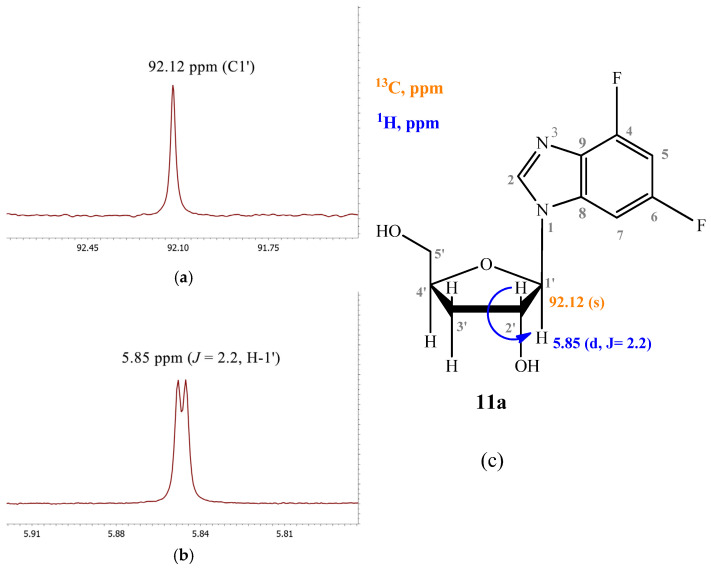
(**a**) fragment of ^13^C NMR spectrum; (**b**) fragment of ^1^H NMR spectrum of compound **11a**; (**c**) structure of compound **11a** and a schematic of the interactions of some nuclei.

The signal of the C1′ atom of **11a** is a singlet (92.12 ppm) (Figure 6a). The signal of H1′ is a doublet (5.85 ppm, J = 2.2) (Figure 6b).

**Figure 7:**
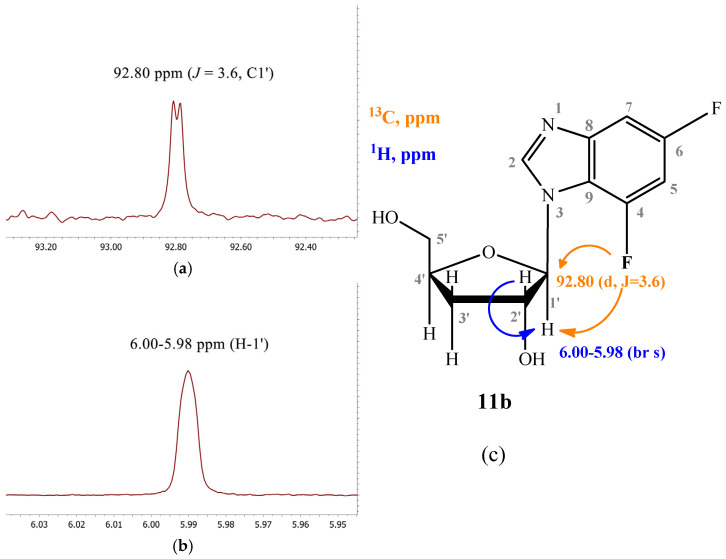
(**a**) fragment of ^13^C NMR spectrum; (**b**) fragment of ^1^H NMR spectrum of compound **11b;** (**c**) structure of compound and a schematic of the interactions of some nuclei.

In the case of compound **11b**, the C1′ signal is split into a doublet (92.80 ppm, J = 3.6) due to long-range interaction with the F nucleus at C4 (Figure 7a). The H1′ signal is broad (6.00–5.98 ppm) due to the proximity of the coupling constants H1′–H2′ and H1′–F4 (Figure 7b).

**Figure 8:**
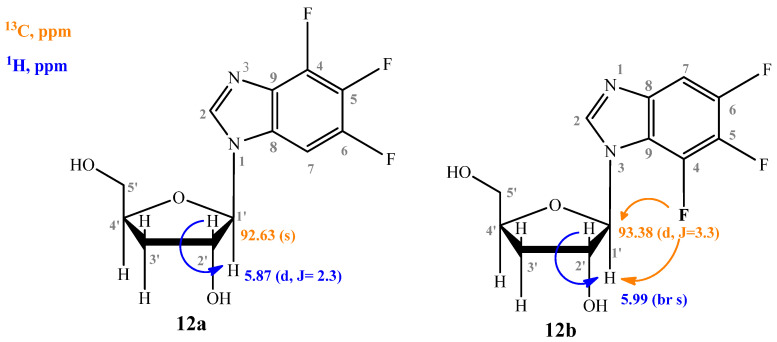
Structures of compounds **12a** and **12b**, and the interaction scheme of some of the nuclei.

The structures of the N1- and N3-regioisomers of nucleoside **12** were established in a similar manner (Figure 8).

Purine nucleoside phosphorylase is capable of glycosylation at both nitrogen atoms of the imidazole moiety of the heterocyclic base. The heterocyclic base binds to the enzyme active site through one of the N-atoms (equally probable), while glycosylation proceeds on the second one.

The ratio of N1- and N3-regioisomers depends on the nucleophilcity of the N atoms in the benzimidazole cycle. In turn, the nucleophilcity depends on the combination of the -I and +M effect strength (the influence of F atoms). In the case of **11**, the N atoms’ nucleophilcity is almost equal (the ratio of N1-isomer:N3-isomer is 54:46). The addition of C5-F (**12**) decreases an electronic density on N3; as a result, this ratio becomes 73:27. The replacement of C5-F by C5-OCH_3_ (**13**) (relatively low −I, relatively high +M) results in the dramatic increase of electron density on N1 (and, correspondingly, nucleophilcity) and only N1-isomer forms. The N3-isomer should form, too, but the percent of this isomer is too small, and we cannot detect it.

It was previously shown that 2-aminobenzimidazole is a good substrate for *E. coli* PNP in ribosylation and 2′-deoxyribosylation reactions. However, in reactions with 3′-deoxyinosine, a sufficient conversion value could not be achieved, so it can be concluded that 2-aminobenzimidazole and 3′-deoxyinosine is a poor combination of substrates for PNP. Therefore, the preparation of nucleoside **14** by the enzymatic method was not feasible due to the low conversion. Also, product **15** could not be obtained and isolated in an amount sufficient for mass spectrometry and NMR because irreversible sorption of the product onto the C18 sorbent occurred.

### 3.2. In Vitro Antiviral Activity of 3′-Deoxyribosides of 1–8

**Figure 9:**
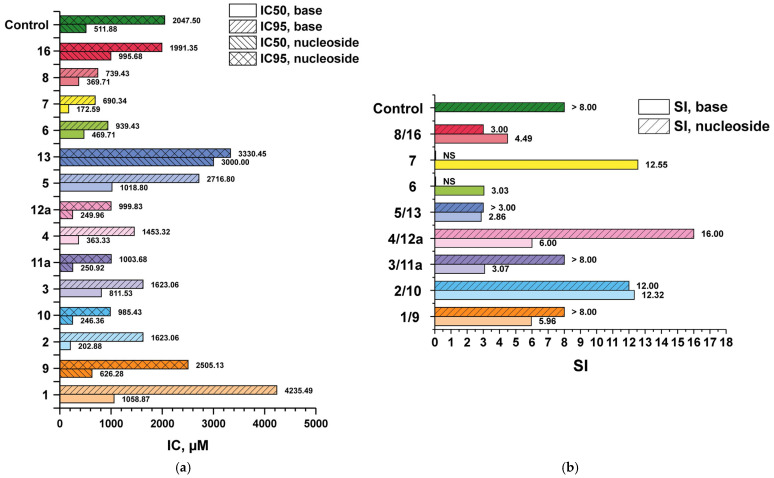
(**a**) IC_50_ and IC_95_ values for nucleosides **9**, **10**, **11a**, **12a**, **13**, **16** and corresponding bases **1**–**8,** obtained from in vitro antiviral activity evaluation using the reference HSV-1/L_2_ strain, where control is ribavirin with IC_50_ and IC_95_ values of 511.88 and 2047.5 μM, respectively; (**b**) SI values for nucleosides **9**, **10**, **11a**, **12a**, **13**, **16** and corresponding bases **1**–**8,** obtained from in vitro antiviral activity evaluation using the reference HSV-1/L_2_ strain, where control is ribavirin with >8.00.

**Figure 10:**
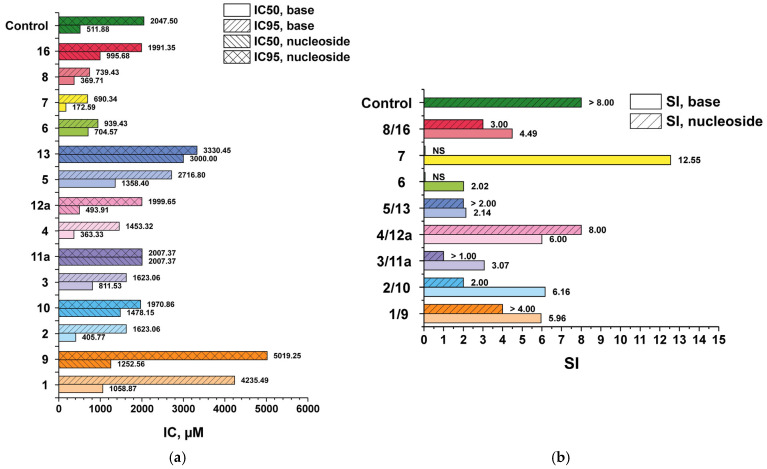
(**a**) IC_50_ and IC_95_ values for nucleosides **9**, **10**, **11a**, **12a**, **13**, **16** and corresponding bases **1**–**8,** obtained from in vitro antiviral activity evaluation using acyclovir-resistant HSV-1/L_2_/R^ACV^ strain, where control is ribavirin, with IC_50_ and IC_95_ values of 511.88 and 2047.5 μM, respectively; (**b**) SI values for nucleosides **9**, **10**, **11a**, **12a**, **13**, **16** and corresponding bases **1**–**8**, obtained from in vitro antiviral activity evaluation using the reference acyclovir-resistant HSV-1/L_2_/R^ACV^ strain, where control is ribavirin with >8.00.

In order to evaluate the effect of benzimidazole chemical modifications on the profiles of antiherpes virus activity and cytotoxicity, the synthesized compounds were tested in vitro using a reference strain of HSV-1/L_2_, including a virus variant profoundly resistant to acyclovir (IC_50_ > 100 μg/mL or >444.05 μM). From the data presented in the diagrams (Figure 9 and Figure 10) and Table S1, it is evident that the initial compound **1**, as well as its 3′-deoxyriboside **9**, were of little toxicity to Vero E6 cell culture, and the minimum antiviral activity (the minimum inhibitory concentration IC_50_) these compounds exhibited was in the range of non-cytotoxic concentrations.

To evaluate the antiviral properties of the obtained compounds, another criterion was used—selectivity index (SI) (Figure 9b and Figure 10b). SI is the ratio of antiviral efficacy of a drug substance to its toxicity (CD_50_/IC_50_; CD_50_—50% cytotoxic concentration of compound, required to reduce the viability of Vero E6 cells by 50%). The higher the SI value, the more effective and safer the drug is considered to be in the treatment of viral infection.

Modifications of base **1** and, respectively, its 3′-deoxyriboside **9**, resulted in increased cytotoxicity. Nevertheless, the 3′-deoxyribosides were less toxic than their corresponding benzimidazole derivatives (pairs **4/12a**, **5/13**, **8/16**) or the cytotoxicity (CC_50_) values were comparable (pairs **2/10**, **3/11a**).

Substitution of the hydrogen atom with fluorine at the C4 and C5 positions of the benzimidazole ring (compounds **3/11a**) or at C5 and C6 (compounds **2/10**) resulted in an increase in cytotoxicity, whereas the antiviral activity parameters (IC_50_/IC_95_) did not change significantly compared to the pair of compounds **1/9**. The trifluoro-substituted benzimidazole analogue **4** was also characterized by higher toxicity to cell culture, but the IC_50_/IC_95_ values were reduced to the same extent. Thus, the selectivity index (SI) values characterizing the selectivity of the antiviral action of bases **4** and **1** were equal. However, the 3′-deoxyriboside of base **4** (N1-regioisomer **12a**) was not only less cytotoxic but also a more active antiviral agent. Accordingly, the SI value of compound **12a** was higher (16). In terms of SI value, compound **12a** outperformed all the studied compounds. Substitution of the fluorine atom with the oxymethyl group in the C5 position of the heterocyclic base of compounds **5/13** led to a significant decrease in the antiviral activity of compounds in comparison with the pair **4/12a**, which was reflected in the SI value.

Compounds containing an amino group in the C2 position (**6**, **7**, **8**, **16**) were more toxic to cell culture but inhibited the development of virus-induced cytopathic effect at lower concentrations than **1** and **9**. Nevertheless, the SI index, which characterizes the selectivity of their anti-herpetic action, was comparable to the SI of compounds **1** and **9**. The exception was base **7** with SI = 12.55. Probably, the replacement of fluorine with an oxymethyl group at the C6 position contributed not only to the reduction of cytotoxicity but also to the increase of antiviral activity (pair **7/15**). Unfortunately, the antiviral activity of nucleoside **15** (3′-deoxyriboside of base **7**) has not been tested to date due to its unavailability. The chemotherapeutic characteristics of 3′-deoxyriboside can be expected to be more favorable than base **7**.

It is interesting that the antiviral activity of both the benzimidazole derivatives and their corresponding 3′-deoxyribosides was maintained against the virus strain resistant to acyclovir, which we characterized earlier as TK-negative. Apparently, the group of compounds under consideration has a different mechanism of activation (nucleotide synthesis) not mediated by the action of viral thymidine kinase due to a significant difference in the structure of the benzimidazole base and the structure of triazole carboxamide.

It cannot be excluded that benzimidazole derivatives and their 3′-deoxyribosides suppress HSV reproduction by inhibiting, for example, the virus terminase complex, similar to the compounds TCRB and BDCRB, which have activity against HCMV [45].

Our results allow us to predict the structures of potentially more effective anti-HSV agents.

### 3.3. Antiviral Activity—Comparative Data

The antiviral activity and in vitro cytotoxicity of the obtained ribosides (Rib), 2′-deoxyribosides (2′-dRib), and 2′-deoxy-2′-fluoroarabinosides (2′-F-Ara) substituted for benzimidazole analogues **2**–**8** were evaluated in [29,38] using the reference strain HSV-1/L_2_.

**Figure 11:**
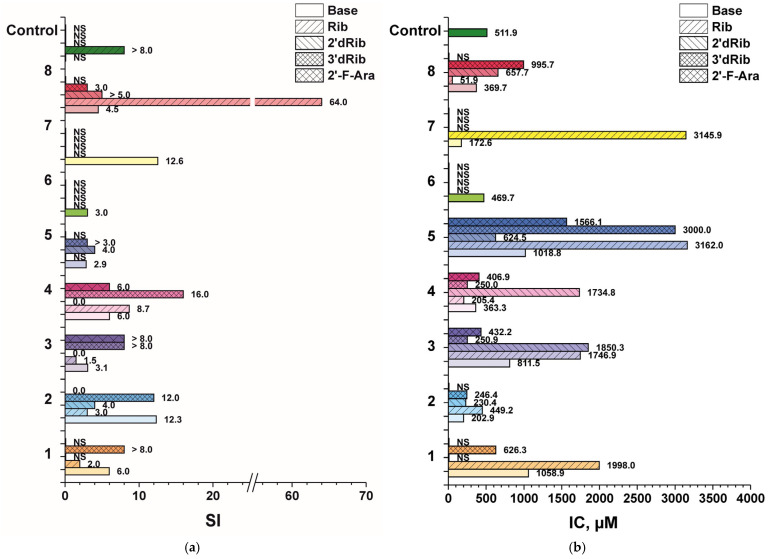
(**a**) IC_50_ values for the Rib, 2′-dRib, 3′-dRib, 2′-F-Ara nucleosides of benzimidazole **1** and its substituted analogues **2**–**8**, where control is ribavirin with IC_50_ values of 511.88 μM; (**b**) SI values for the Rib, 2′-dRib, 3′-dRib, 2′-F-Ara nucleosides of benzimidazole **1** and its substituted analogues **2**–**8**, where control is ribavirin with >8.00.

We decided to compare the antiviral activity of the 3′-deoxyribosides (3′-dRib) and the previously obtained Rib, 2′-dRib, 2′-F-Ara benzimidazole 1 and its substituted analogues **2**–**8**, as well as heterocyclic bases **1**–**8** themselves (Figure 11). Ribavirin was chosen as a comparison drug (control) because it is the closest structurally similar antiviral compound with proven efficacy against HSV-1 in vitro [46] to the tested compounds.

The presence of carbohydrate residue affected the values of minimal antiviral activity (Figure 11a). In most cases, the IC_50_ of the nucleosides synthesized was higher than the IC_50_ of the corresponding bases. This was probably due to the improved bioavailability of the molecules and the presence of nucleoside transport systems through the cytoplasmic membrane.

At the same time, SI values (Figure 11b) were higher for benzimidazoles 3 and 8 than for the corresponding nucleosides and ribavirin.

## 4. Conclusions

The substrate specificity of PNP towards substituted benzimidazoles was studied. The conditions for the synthesis of 3′-deoxyribosides of benzimidazole and its substituted analogues—substrate ratio, enzyme concentration, and DMSO concentration—were optimized. The addition of 5% DMSO was shown to increase the conversion of 3′-deoxyinosine to the product by 5–30% in reactions where bases are in excess. The synthesis of eight new substituted benzimidazole nucleosides was carried out. In the case of asymmetrically substituted bases, N1- and N3-nucleosides were formed, which was proved by the analysis of ^1^H and ^13^C NMR spectra. The antiviral activity and cytotoxicity of the nucleosides obtained were investigated. 4,6-Difluoro-1-(β-D-3′-deoxyribofuranosyl)benzimidazole, 4,5,6-trifluoro-1-(β-D-3′-deoxyribofuranosyl) benzimidazole, 4,6-difluoro-benzimidazole, and 2-amino-5-fluoro-6-O-methoxy-benzimidazole showed pronounced activity against herpes simplex virus 1 (SI = 12.00, 16.00, 12.32, and 12.55 correspondingly). The obtained data on the activity against HSV-1 of the lead compounds make them interesting and promising targets for further in vitro and in vivo property studies.

## Figures and Tables

**Table 1:** Optimized conditions for trial enzymatic reactions.

Compound (Base)	Base Structure	Base: 3′-dIno Ratio (by Moles)	PNP Concentration (Units/mL)	Conversion, % in 168 h
**9 (1)**	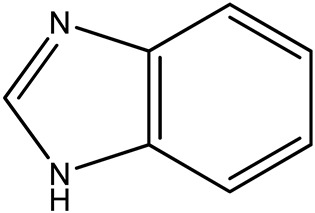	9:1	21	78
**10 (2)**	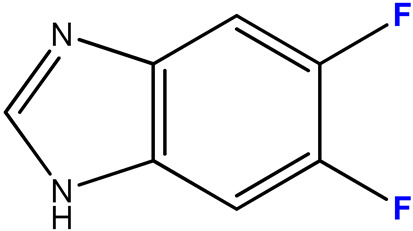	9:1	7	80
**11 (3)**	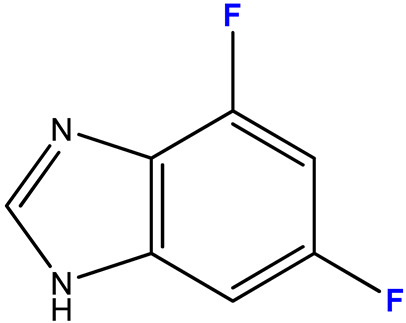	1:5	7	81
**12 (4)**	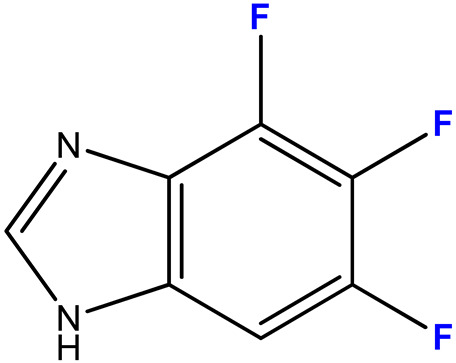	9:1	7	68
**13 (5)**	** 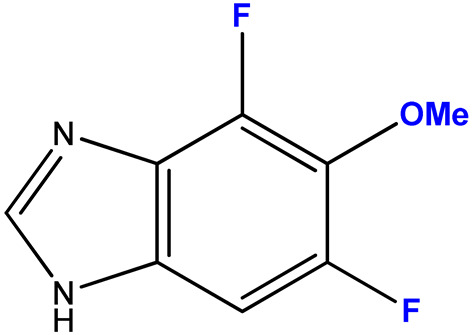 **	1:7	7	72
**14 (6)**	** 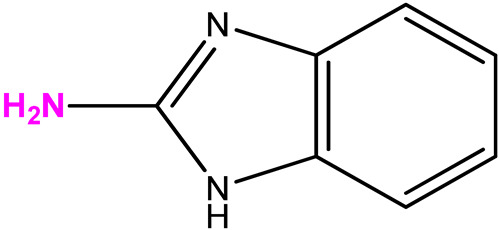 **	9:1	21	7
**15 (7)**	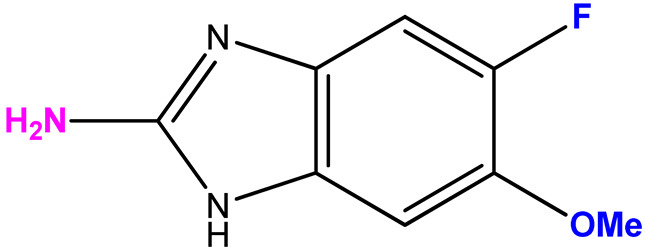	9:1	10.5	28
**16 (8)**	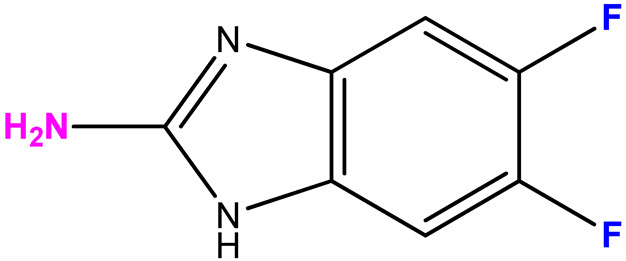	9:1	21	61

**Table 2:** Experimental data for the synthesis of nucleosides **9**–**13**, **16**.

Compound	Base, mg (mmol)	3′-dIno, mg (mmol)	Ratio Base: 3′dIno (by Moles)	PNP, Units	Potassium Phosphate (pH 7.0)/Volume, mL	Time, Days	Isomer Ratio (HPLC Data)		Conversion/Yield, % (mg)
							N-1	N-3	
**9**	**1**—271 (2.29)	64 (0.25)	9:1	4200	10 mM/200	20	-	-	54/27 (16)
**10**	**2**—182.58 (1.18)	100 (0.39)	3:1	1400	10 mM/100	40	-	-	93/32 (33.2)
**11**	**3**—25 (0.16)	205(0.81)	1:5	1400	6 mM/200	40	54	46	73/46 (7)
**12**	**4**—110 (0.64)	32.37 (0.13)	5:1	1400	10 mM/100	40	73	27	79/46 (17)
**13**	**5**—23 (0.12)	215 (0.85)	1:7	2100	6 mM/100	2	-	-	87/58 (20)
**16**	**8**—601 (3.5)	100 (0.4)	9:1	3500	9 mM/200	40	-	-	62/8 (8.5)

## Data Availability

The original contributions presented in this study are included in the article/Supplementary Materials. Further inquiries can be directed to the corresponding authors.

## Supplementary Materials

The following supporting information can be downloaded at https://www.mdpi.com/article/10.3390/biom15070922/s1, Figure S1. Dependence of conversion on the ratio of base 1 to 3′-dIno. Conditions: reaction volume 1 mL, 50 °C, 2 mM potassium phosphate (pH 7.0), PNP 7 units/mL. Figure S2. Dependence of 3′-dIno conversion into nucleoside **9** on PNP concentration. Conditions: base **1** to 3′-dIno ratio—9:1; reaction volume 1 mL, 50 °C, 2 mM potassium phosphate (pH 7.0). Figure S3. Dependence of conversion on the ratio of base **2** to 3′-dIno. Conditions: reaction volume 1 mL, 50 °C, 2 mM potassium phosphate (pH 7.0), PNP 7 units/mL. Figure S4. Dependence of 3′-dIno conversion into nucleoside **10** on PNP concentration. Conditions: base **2** to 3′-dIno ratio—9:1; reaction volume 1 mL, 50 °C, 2 mM potassium phosphate (pH 7.0). Figure S5. Dynamics of products **11a**, **11b** accumulation (total) depending on the ratio base **3** to 3′-dIno. Conditions: reaction volume 1 mL, 50 °C, 6 mM potassium phosphate (pH 7.0), PNP 7 units/mL. Figure S6. Dynamics of products **11a**, **11b** accumulation (total) depending on PNP concentration. Conditions: base **3** to 3′-dIno ratio—1:5; reaction volume 1 mL, 50 °C, 2 mM potassium phosphate (pH 7.0). Figure S7. Dynamics of products **12a**, **12b** accumulation (total) depending on the ratio base **4** to 3′-dIno. Conditions: reaction volume 1 mL, 50 °C, 2 mM potassium phosphate (pH 7.0), PNP 7 units/mL. Figure S8. Dynamics of products **12a**, **12b** accumulation (total) depending on PNP concentration. Conditions: base **4** to 3′-dIno ratio—9:1; reaction volume 1 mL, 50 °C, 2 mM potassium phosphate (pH 7.0). Figure S9. Dynamics of product **13** accumulation depending on the ratio base **5** to 3′-dIno. Conditions: reaction volume 1 mL, 50 °C, 9 mM potassium phosphate (pH 7.0), PNP 7 units/mL. Figure S10. Dynamics of product **13** accumulation depending on PNP concentration. Conditions: base **5** to 3′-dIno ratio—1:7; reaction volume 1 mL, 50 °C, 2 mM potassium phosphate (pH 7.0). Figure S11. Dynamics of product **14** accumulation depending on the ratio base **6** to 3′-dIno. Conditions: reaction volume 1 mL, 50 °C, 2 mM potassium phosphate (pH 7.0), PNP 7 units/mL. Figure S12. Dynamics of product **14** accumulation depending on PNP concentration. Conditions: base **6** to 3′-dIno ratio—9:1; reaction volume 1 mL, 50 °C, 2 mM potassium phosphate (pH 7.0). Figure S13. Dynamics of product **15** accumulation depending on the ratio base **7** to 3′-dIno. Conditions: reaction volume 1 mL, 50 °C, 2 mM potassium phosphate (pH 7.0), PNP 7 units/mL. Figure S14. Dynamics of product **15** accumulation depending on PNP concentration. Conditions: base **7** to 3′-dIno ratio—9:1; reaction volume 1 mL, 50 °C, 2 mM potassium phosphate (pH 7.0). Figure S15. Dynamics of product **16** accumulation depending on the ratio base **8** to 3′-dIno. Conditions: reaction volume 1 mL, 50 °C, 2 mM potassium phosphate (pH 7.0), PNP 7 units/mL. Figure S16. Dynamics of product **16** accumulation depending on PNP concentration. Conditions: base **7** to 3′-dIno ratio—9:1; reaction volume 1 mL, 50 °C, 2 mM potassium phosphate (pH 7.0). Figure S17. The ^1^H NMR spectrum of 1-(β-D-3′-deoxyribofuranosyl)benzimidazole **9.** Figure S18. The ^13^C NMR spectrum of 1-(β-D-3′-deoxyribofuranosyl)benzimidazole **9**. Figure S19. The ^1^H-^15^N HMBC NMR spectrum of 1-(β-D-3′-deoxyribofuranosyl)benzimidazole **9**. Figure S20. The ^1^H NMR spectrum of 5,6-difluoro-1-(β-D-3′-deoxyribofuranosyl)benzimidazole **10**. Figure S21. The ^13^C NMR spectrum of 5,6-difluoro-1-(β-D-3′-deoxyribofuranosyl)benzimidazole **10**. Figure S22. The ^1^H-^15^N HMBC NMR spectrum of 5,6-difluoro-1-(β-D-3′-deoxyribofuranosyl)benzimidazole **10**. Figure S23. The ^1^H NMR spectrum of 4,6-difluoro-1-(β-D-3′-deoxyribofuranosyl)benzimidazole **11a** (N1-isomer). Figure S24. The ^13^C NMR spectrum of 4,6-difluoro-1-(β-D-3′-deoxyribofuranosyl)benzimidazole **11a** (N1-isomer). Figure S25. The ^1^H-^15^N HMBC NMR spectrum of 4,6-difluoro-1-(β-D-3′-deoxyribofuranosyl)benzimidazole **11a** (N1-isomer). Figure S26. The ^1^H NMR spectrum of 4,6-difluoro-1-(β-D-3′-deoxyribofuranosyl)benzimidazole **11b** (N3-isomer). Figure S27. The ^13^C NMR spectrum of 4,6-difluoro-1-(β-D-3′-deoxyribofuranosyl)benzimidazole **11b** (N3-isomer). Figure S28. The ^1^H-^15^N HMBC NMR spectrum of 4,6-difluoro-1-(β-D-3′-deoxyribofuranosyl)benzimidazole **11b** (N3-isomer). Figure S29. The ^1^H NMR spectrum of 4,5,6-trifluoro-1-(β-D-3′-deoxyribofuranosyl)benzimidazole **12a** (N1-isomer). Figure S30. The ^13^C NMR spectrum of 4,5,6-trifluoro-1-(β-D-3′-deoxyribofuranosyl)benzimidazole **12a** (N1-isomer). Figure S31. The ^1^H-^15^N HMBC NMR spectrum of 4,5,6-trifluoro-1-(β-D-3′-deoxyribofuranosyl)benzimidazole **12a** (N1-isomer). Figure S32. The ^1^H NMR spectrum of 4,5,6-trifluoro-1-(β-D-3′-deoxyribofuranosyl)benzimidazole **12b** (N3-isomer). Figure S33. The ^13^C NMR spectrum of 4,5,6-trifluoro-1-(β-D-3′-deoxyribofuranosyl)benzimidazole **12b** (N3-isomer). Figure S34. The ^1^H-^15^N HMBC NMR spectrum of 4,5,6-trifluoro-1-(β-D-3′-deoxyribofuranosyl)benzimidazole **12b** (N3-isomer). Figure S35. The ^1^H NMR spectrum of 4,6-difluoro-5-metoxy-1-(β-D-3′-deoxyribofuranosyl)benzimidazole **13**. Figure S36. The ^13^C NMR spectrum of 4,6-difluoro-5-metoxy-1-(β-D-3′-deoxyribofuranosyl)benzimidazole **13**. Figure S37. The ^1^H-^15^N HMBC NMR spectrum of 4,6-difluoro-5-metoxy-1-(β-D-3′-deoxyribofuranosyl)benzimidazole **13**. Figure S38. The ^1^H NMR spectrum of 2-amino-5,6-difluoro-1-(β-D-3′-deoxyribofuranosyl)benzimidazole **16**. Figure S39. The ^13^C NMR spectrum of 2-amino-5,6-difluoro-1-(β-D-3′-deoxyribofuranosyl)benzimidazole **16**. Figure S40. The ^1^H-^15^N HMBC NMR spectrum of 2-amino-5,6-difluoro-1-(β-D-3′-deoxyribofuranosyl)benzimidazole **16**. Figure S41. The ^1^H-^15^N HSQC NMR spectrum of 2-amino-5,6-difluoro-1-(β-D-3′-deoxyribofuranosyl)benzimidazole **16**. Table S1. In vitro Antiviral Activity of Compounds **1**–**13**, **16** against HSV-1.

## Abbreviations

The following abbreviations are used in this manuscript:

PNP	purine nucleoside phosphorylase
WHO	World Health Organization
FDA	Food and Drug Administration
HSV	herpes simplex virus
HCMV	human cytomegalovirus
EBV	Epstein–Barr virus
DRB	5,6-dichloro-1-(β-D-ribofuranosyl)benzimidazole
TCRB	2,5,6-trichloro-1-(β-D-ribofuranosyl)benzimidazole
BDCRB	2-bromo-5,6-dichloro-1-(β-D-ribofuranosyl)benzimidazole
MBV	Maribavir
DMSO	dimethyl sulfoxide
Hyp	hypoxantine
Pi	inorganic phosphate
TK	thymidine kinase
MOI	multiplicity of infection
SI	selectivity index
CPE	cytopathic effect
