# Supplementary material for: New Benzimidazole 3′-Deoxynucleosides: Synthesis and Antiherpes Virus Properties
Source: Biomolecules. 2025 Jun 23;15(7):922. doi: 10.3390/biom15070922 (PMC12293048; doi:10.3390/biom15070922)
Supplement: Supplementary file 1 [file biomolecules-15-00922-s001.zip › biomolecules-3637490-supplementary.pdf]

Supporting Information

New benzimidazole 3'-deoxynucleosides: synthesis and antiherpes virus properties.

Aleksandra O. Arnautova[1, 2, *], Irina A. Aleksakhina[1], Ekaterina A. Zorina[1], Maria Ya. Berzina[1], Ilya V. Fateev[1], Barbara Z. Eletskaya[1], Konstantin V. Antonov[1], Olga S. Smirnova[1], Alexander S. Paramonov[1], Alexey L. Kayushin[1], Valeria L. Andronova[3], Georgii A. Galegov[3], Maria A. Kostromina[1], Evgeny A. Zayats[1], Inna L. Karpenko[2], Svetlana K. Kotovskaya[4, 5], Valery N. Charushin[4, 5], Roman S. Esipov[1], Anatoly I. Miroshnikov[1], Irina D. Konstantinova[1]

[1]Shemyakin-Ovchinnikov Institute of Bioorganic Chemistry, Russian Academy of Sciences, Miklukho-Maklaya St. 16/10, 117997 GSP, Moscow B-437, Russian Federation
[2] Engelhardt Institute of Molecular Biology Russian Academy of Sciences, Vavilova St. 32, Moscow, 119991 Russian Federation
[3]Ivanovsky Institute of Virology (Gamaleya National Research Center for Epidemiology, Ministry of Healthcare of the Russian Federation), Gamaleya St. 18, 123098, Moscow, Russian Federation
[4]Postovsky Institute of Organic Synthesis, The Ural Branch of the Russian Academy of Sciences, S. Kovalevskaya/Academicheskaya St. 22/20, 620041, Ekaterinburg, Russian Federation
[5]Yeltsin Ural Federal University, N., Mira St. 19, 620002, Ekaterinburg, Russian Federation

Content	Page number
Figure SI-1. Dependence of conversion on the ratio of base 1 to 3'-dIno. Conditions: reaction volume 1 ml, 50 °C, 2 mM potassium phosphate (pH 7.0), PNP 7 units/ml.	4
Figure SI-2. Dependence of 3'-dIno conversion into nucleoside **9** on PNP concentration. Conditions: base **1** to 3'-dIno ratio - 9:1; reaction volume 1 ml, 50 °C, 2 mM potassium phosphate (pH 7.0).	4
Figure SI-3. Dependence of conversion on the ratio of base **2** to 3'-dIno. Conditions: reaction volume 1 ml, 50 °C, 2 mM potassium phosphate (pH 7.0), PNP 7 units/ml.	5
Figure SI-4. Dependence of 3'-dIno conversion into nucleoside **10** on PNP concentration. Conditions: base **2** to 3'-dIno ratio - 9:1; reaction volume 1 ml, 50 °C, 2 mM potassium phosphate (pH 7.0).	5
Figure SI-5. Dynamics of products **11a, 11b** accumulation (total) depending on the ratio base **3** to 3'-dIno. Conditions: reaction volume 1 ml, 50 °C, 6 mM potassium phosphate (pH 7.0), PNP 7 units/ml.	6
Figure SI-6. Dynamics of products **11a, 11b** accumulation (total) depending on PNP concentration. Conditions: base **3** to 3'-dIno ratio – 1:5; reaction volume 1 ml, 50 °C, 2 mM potassium phosphate (pH 7.0).	6
Figure SI-7. Dynamics of products **12a, 12b** accumulation (total) depending on the ratio base **4** to 3'-dIno. Conditions: reaction volume 1 ml, 50 °C, 2 mM potassium phosphate (pH 7.0), PNP 7 units/ml.	7
Figure SI-8. Dynamics of products **12a, 12b** accumulation (total) depending on PNP concentration. Conditions: base **4** to 3'-dIno ratio – 9:1; reaction volume 1 ml, 50 °C, 2 mM potassium phosphate (pH 7.0).	7
Figure SI-9. Dynamics of product **13** accumulation depending on the ratio base **5** to 3'-dIno. Conditions: reaction volume 1 ml, 50 °C, 9 mM potassium phosphate (pH 7.0), PNP 7 units/ml.	8

Figure SI-10. Dynamics of product **13** accumulation depending on PNP concentration. Conditions: base **5** to 3'-dIno ratio – 1:7; reaction volume 1 ml, 50 °C, 2 mM potassium phosphate (pH 7.0).	8
Figure SI-11. Dynamics of product **14** accumulation depending on the ratio base **6** to 3'-dIno. Conditions: reaction volume 1 ml, 50 °C, 2 mM potassium phosphate (pH 7.0), PNP 7 units/ml.	9
Figure SI-12. Dynamics of product **14** accumulation depending on PNP concentration. Conditions: base **6** to 3'-dIno ratio – 9:1; reaction volume 1 ml, 50 °C, 2 mM potassium phosphate (pH 7.0).	9
Figure SI-13. Dynamics of product **15** accumulation depending on the ratio base **7** to 3'-dIno. Conditions: reaction volume 1 ml, 50 °C, 2 mM potassium phosphate (pH 7.0), PNP 7 units/ml.	10
Figure SI-14. Dynamics of product **15** accumulation depending on PNP concentration. Conditions: base **7** to 3'-dIno ratio – 9:1; reaction volume 1 ml, 50 °C, 2 mM potassium phosphate (pH 7.0).	10
Figure SI-15. Dynamics of product **16** accumulation depending on the ratio base **8** to 3'-dIno. Conditions: reaction volume 1 ml, 50 °C, 2 mM potassium phosphate (pH 7.0), PNP 7 units/ml.	11
Figure SI-16. Dynamics of product **16** accumulation depending on PNP concentration. Conditions: base **7** to 3'-dIno ratio – 9:1; reaction volume 1 ml, 50 °C, 2 mM potassium phosphate (pH 7.0).	11
Figure SI-17. The ^{1}H NMR spectrum of 1-(β-D-3'-deoxyribofuranosyl)benzimidazole **9.**	12
Figure SI-18. The ^{13}C NMR spectrum of 1-(β-D-3'-deoxyribofuranosyl)benzimidazole **9.**	13
Figure SI-19. The ^{1}H-^{15}N HMBC NMR spectrum of 1-(β-D-3'-deoxyribofuranosyl)benzimidazole **9.**	13
Figure SI-20. The ^{1}H NMR spectrum of 5,6-difluoro-1-(β-D-3'-deoxyribofuranosyl)benzimidazole **10.**	14
Figure SI-21. The ^{13}C NMR spectrum of 5,6-difluoro-1-(β-D-3'-deoxyribofuranosyl)benzimidazole **10.**	15
Figure SI-22. The ^{1}H-^{15}N HMBC NMR spectrum of 5,6-difluoro-1-(β-D-3'-deoxyribofuranosyl)benzimidazole **10.**	15
Figure SI-23. The ^{1}H NMR spectrum of 4,6-difluoro-1-(β-D-3'-deoxyribofuranosyl)benzimidazole **11a** (N1-isomer).	16
Figure SI-24. The ^{13}C NMR spectrum of 4,6-difluoro-1-(β-D-3'-deoxyribofuranosyl)benzimidazole **11a** (N1-isomer).	17
Figure SI-25. The ^{1}H-^{15}N HMBC NMR spectrum of 4,6-difluoro-1-(β-D-3'-deoxyribofuranosyl)benzimidazole **11a** (N1-isomer).	18
Figure SI-26. The ^{1}H NMR spectrum of 4,6-difluoro-1-(β-D-3'-deoxyribofuranosyl)benzimidazole **11b** (N3-isomer).	18
Figure SI-27. The ^{13}C NMR spectrum of 4,6-difluoro-1-(β-D-3'-deoxyribofuranosyl)benzimidazole **11b** (N3-isomer).	19
Figure SI-28. The ^{1}H-^{15}N HMBC NMR spectrum of 4,6-difluoro-1-(β-D-3'-deoxyribofuranosyl)benzimidazole **11b** (N3-isomer).	20

Figure SI-29. The ^{1}H NMR spectrum of 4,5,6-trifluoro-1-(β-D-3'-deoxyribofuranosyl)benzimidazole **12a** (N1-isomer).	20
Figure SI-30. The ^{13}C NMR spectrum of 4,5,6-trifluoro-1-(β-D-3'-deoxyribofuranosyl)benzimidazole **12a** (N1-isomer).	21
Figure SI-31. The ^{1}H-^{15}N HMBC NMR spectrum of 4,5,6-trifluoro-1-(β-D-3'-deoxyribofuranosyl)benzimidazole **12a** (N1-isomer).	22
Figure SI-32. The ^{1}H NMR spectrum of 4,5,6-trifluoro-1-(β-D-3'-deoxyribofuranosyl)benzimidazole **12b** (N3-isomer).	22
Figure SI-33. The ^{13}C NMR spectrum of 4,5,6-trifluoro-1-(β-D-3'-deoxyribofuranosyl)benzimidazole **12b** (N3-isomer).	23
Figure SI-34. The ^{1}H-^{15}N HMBC NMR spectrum of 4,5,6-trifluoro-1-(β-D-3'-deoxyribofuranosyl)benzimidazole **12b** (N3-isomer).	24
Figure SI-35. The ^{1}H NMR spectrum of 4,6-difluoro-5-metoxy-1-(β-D-3'-deoxyribofuranosyl)benzimidazole **13.**	24
Figure SI-36. The ^{13}C NMR spectrum of 4,6-difluoro-5-metoxy-1-(β-D-3'-deoxyribofuranosyl)benzimidazole **13.**	25
Figure SI-37. The ^{1}H-^{15}N HMBC NMR spectrum of 4,6-difluoro-5-metoxy-1-(β-D-3'-deoxyribofuranosyl)benzimidazole **13.**	26
Figure SI-38. The ^{1}H NMR spectrum of 2-amino-5,6-difluoro-1-(β-D-3'-deoxyribofuranosyl)benzimidazole **16.**	26
Figure SI-39. The ^{13}C NMR spectrum of 2-amino-5,6-difluoro-1-(β-D-3'-deoxyribofuranosyl)benzimidazole **16.**	27
Figure SI-40. The ^{1}H-^{15}N HMBC NMR spectrum of 2-amino-5,6-difluoro-1-(β-D-3'-deoxyribofuranosyl)benzimidazole **16.**	28
Figure SI-41. The ^{1}H-^{15}N HSQC NMR spectrum of 2-amino-5,6-difluoro-1-(β-D-3'-deoxyribofuranosyl)benzimidazole **16.**	29
Table SI-1 – *In vitro* Antiviral Activity of Compounds **1 – 13**, **16** against HSV-1.	29-30

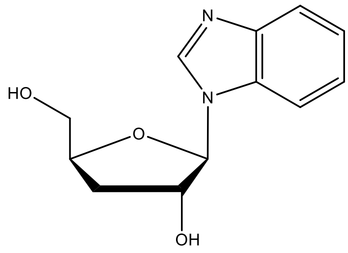

9

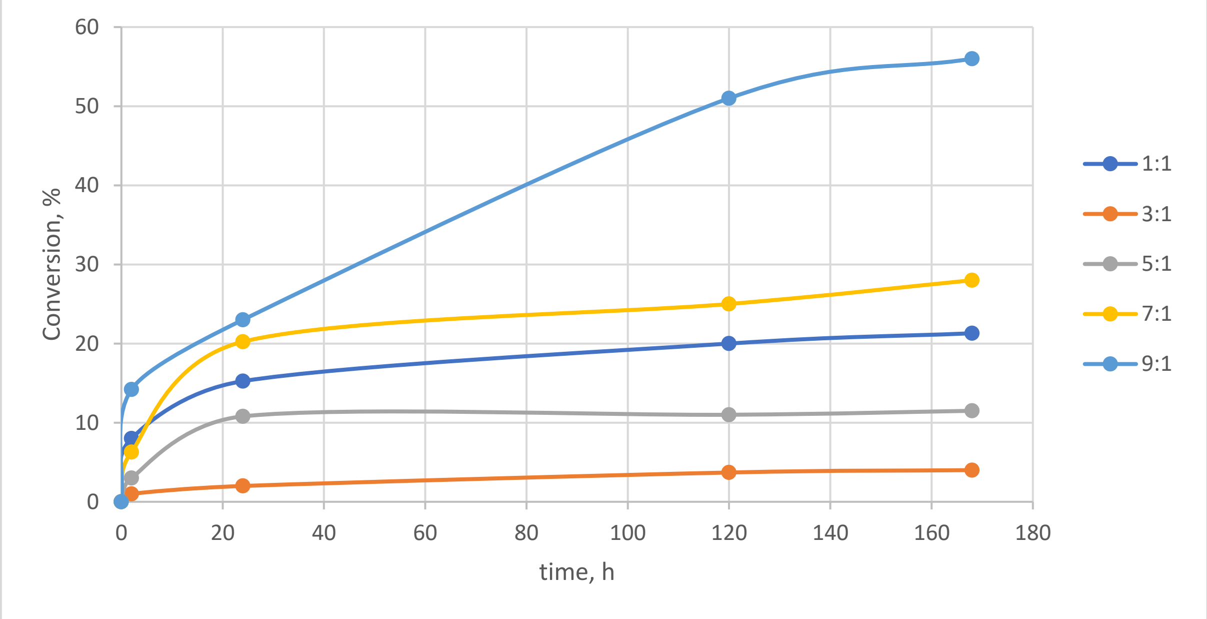

Figure SI-1. Dependence of conversion on the ratio of base 1 to 3'-dIno. Conditions: reaction volume 1 ml, 50 °C, 2 mM potassium phosphate (pH 7.0), PNP 7 units/ml.

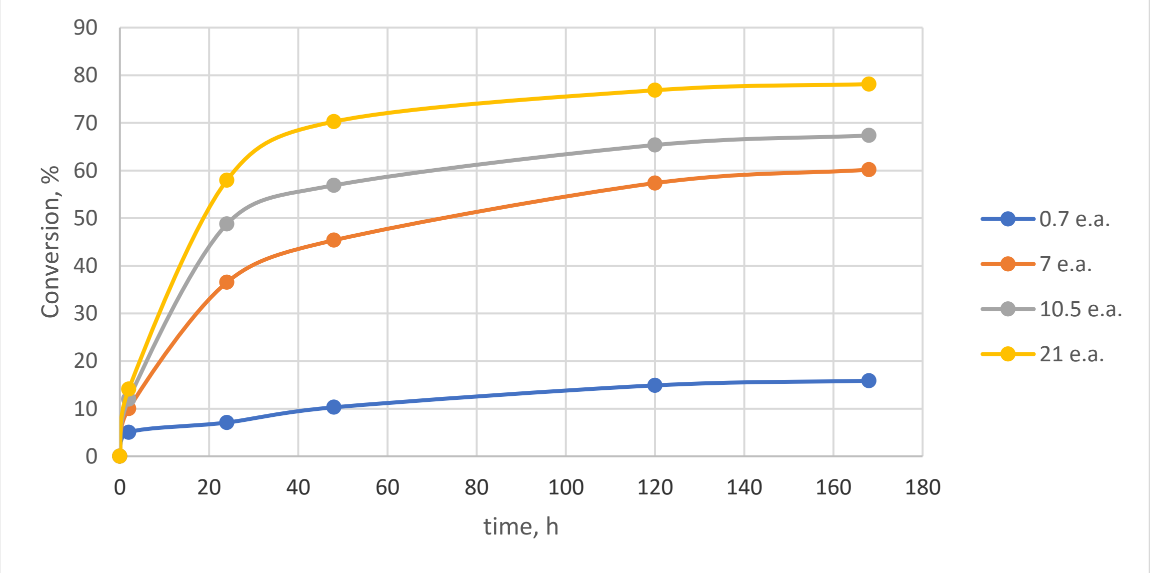

Figure SI-2. Dependence of 3'-dIno conversion into nucleoside **9** on PNP concentration. Conditions: base **1** to 3'-dIno ratio - 9:1; reaction volume 1 ml, 50 °C, 2 mM potassium phosphate (pH 7.0).

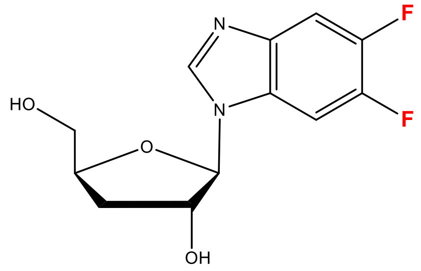

10

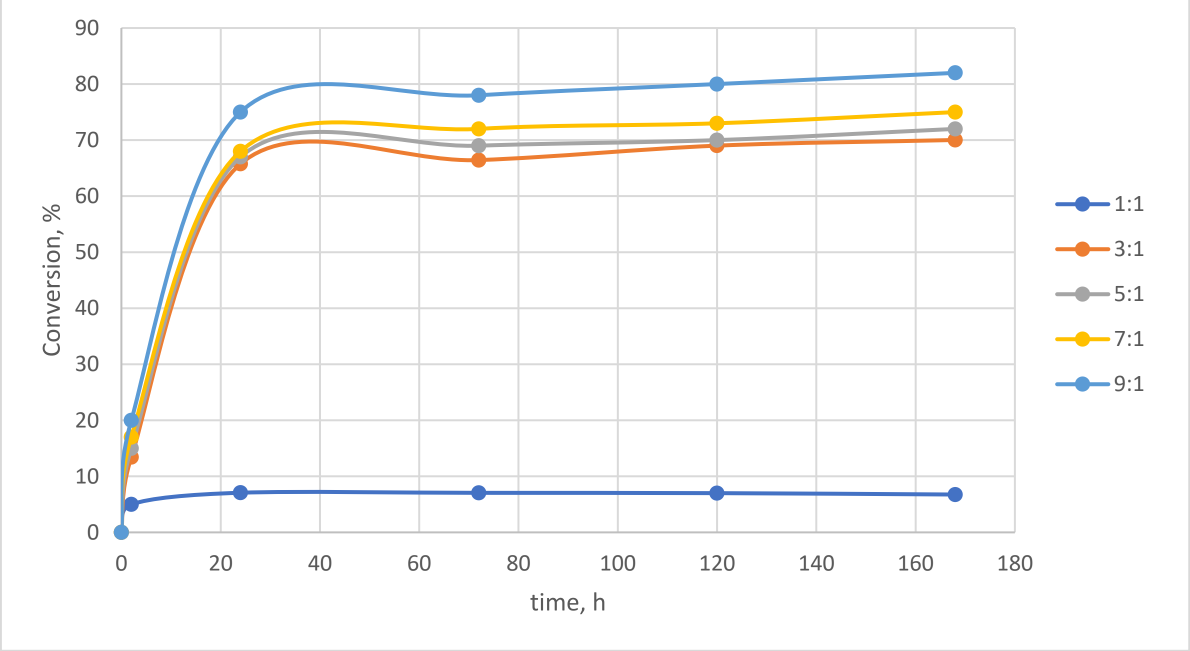

Figure SI-3. Dependence of conversion on the ratio of base **2** to 3'-dIno. Conditions: reaction volume 1 ml, 50 °C, 2 mM potassium phosphate (pH 7.0), PNP 7 units/ml.

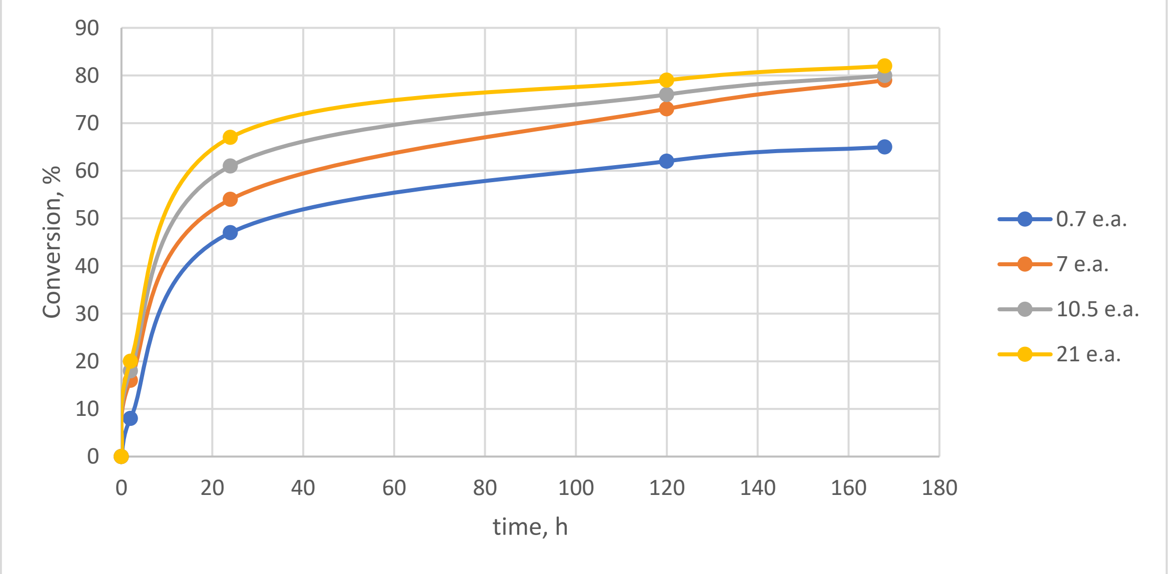

Figure SI-4. Dependence of 3'-dIno conversion into nucleoside **10** on PNP concentration. Conditions: base **2** to 3'-dIno ratio - 9:1; reaction volume 1 ml, 50 °C, 2 mM potassium phosphate (pH 7.0).

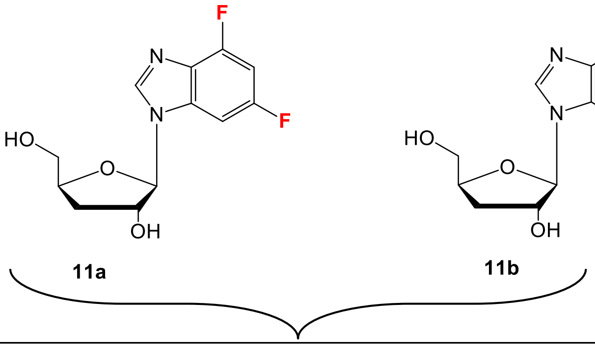

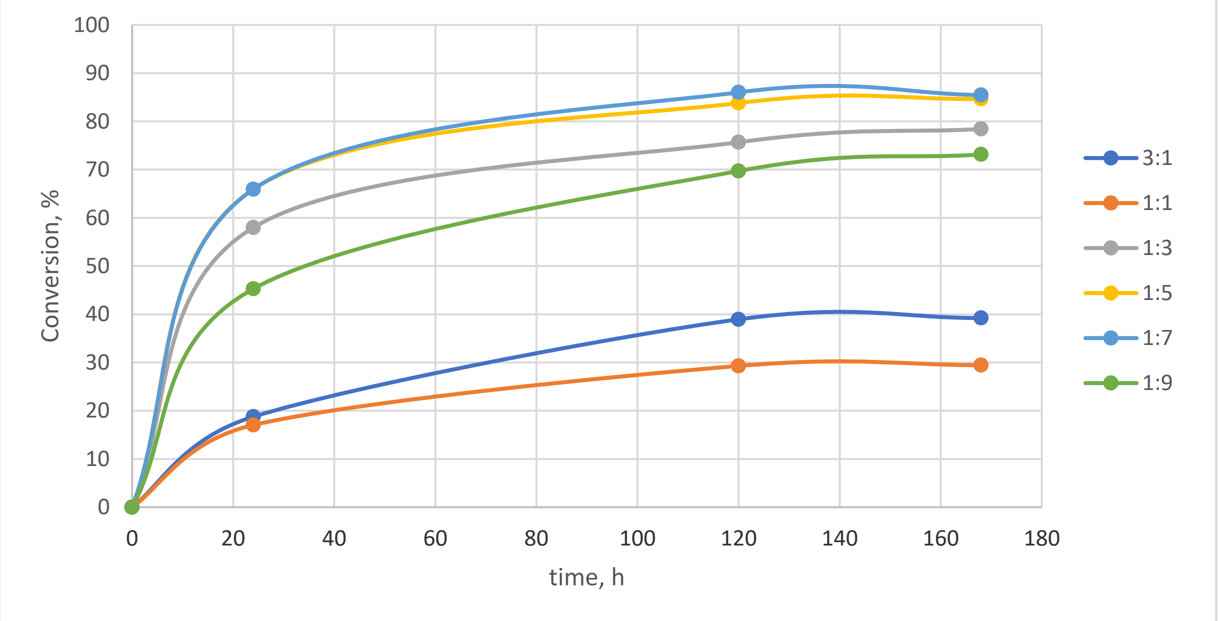

Figure SI-5. Dynamics of products **11a, 11b** accumulation (total) depending on the ratio base **3** to 3'-dIno. Conditions: reaction volume 1 ml, 50 °C, 6 mM potassium phosphate (pH 7.0), PNP 7 units/ml.

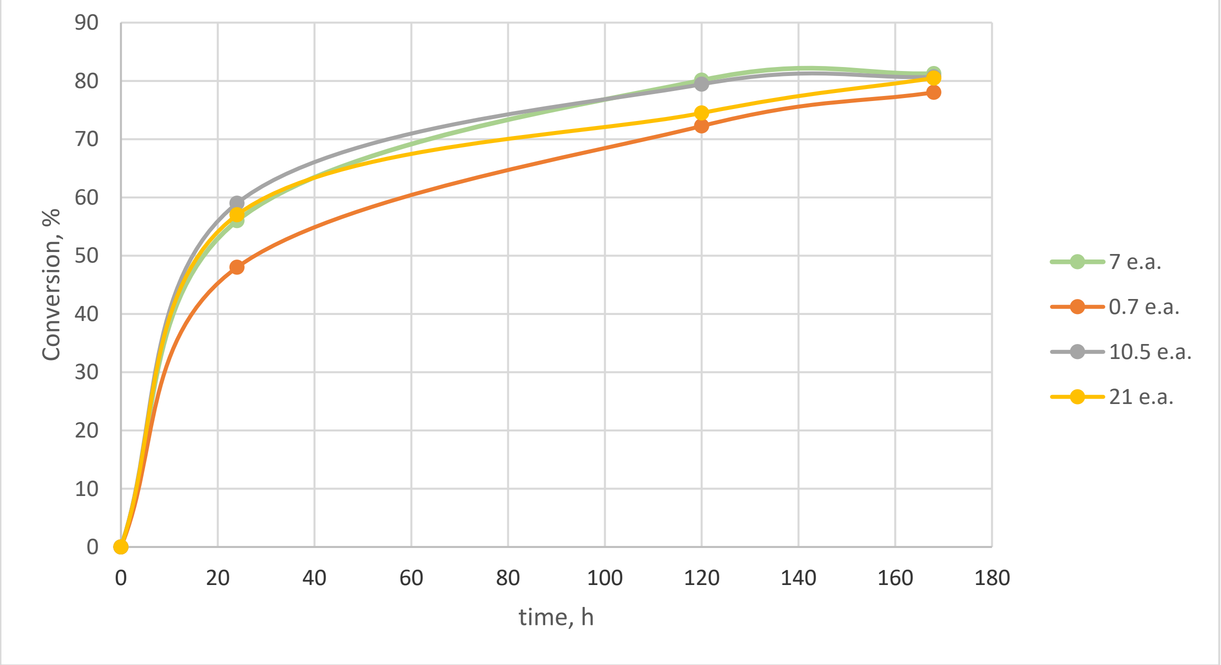

Figure SI-6. Dynamics of products **11a, 11b** accumulation (total) depending on PNP concentration. Conditions: base **3** to 3'-dIno ratio – 1:5; reaction volume 1 ml, 50 °C, 2 mM potassium phosphate (pH 7.0).

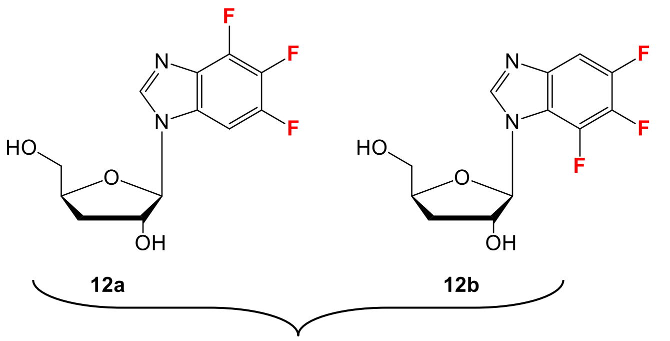

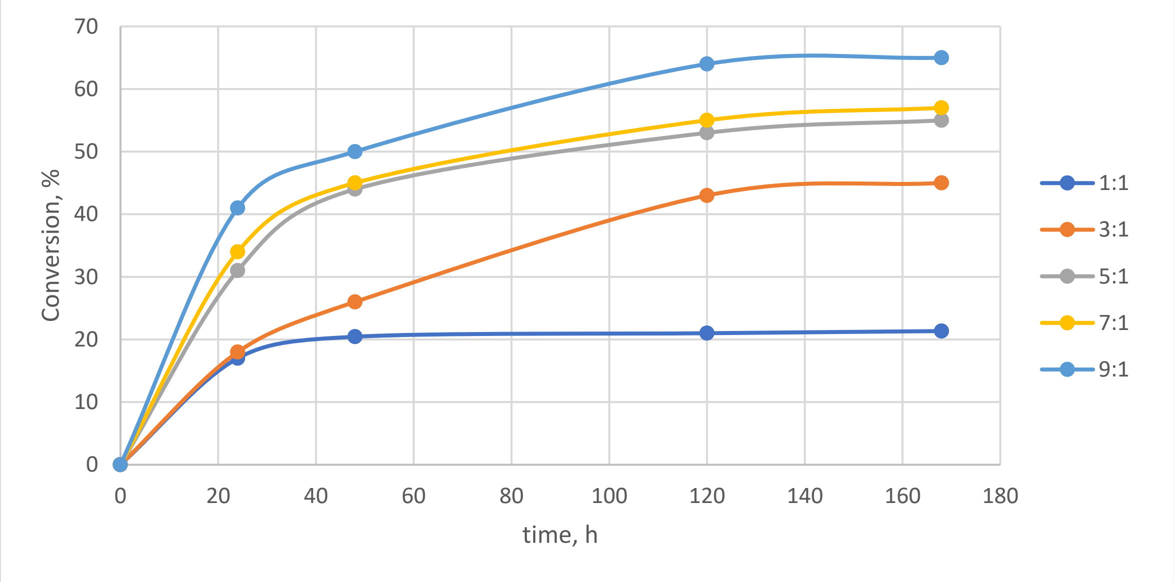

Figure SI-7. Dynamics of products **12a, 12b** accumulation (total) depending on the ratio base **4** to 3'-dIno. Conditions: reaction volume 1 ml, 50 °C, 2 mM potassium phosphate (pH 7.0), PNP 7 units/ml.

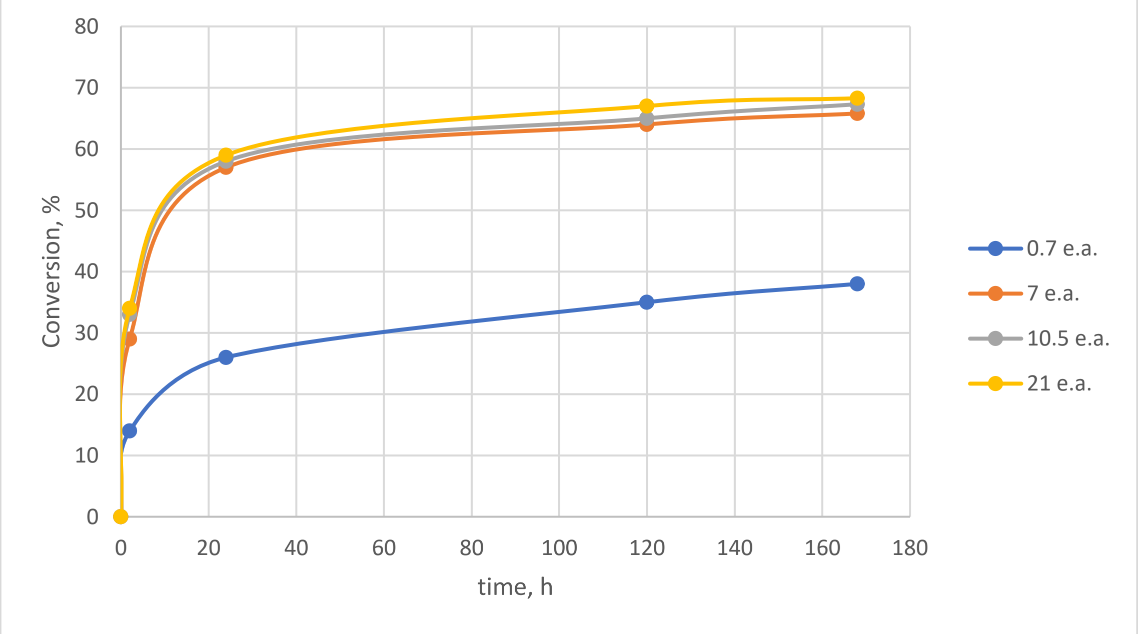

Figure SI-8. Dynamics of products **12a, 12b** accumulation (total) depending on PNP concentration. Conditions: base **4** to 3'-dIno ratio – 9:1; reaction volume 1 ml, 50 °C, 2 mM potassium phosphate (pH 7.0).

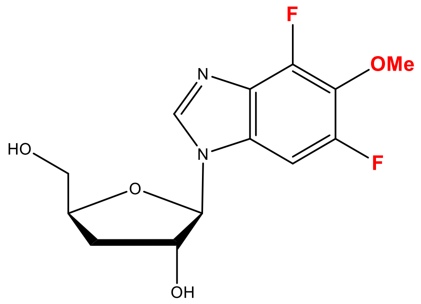

13

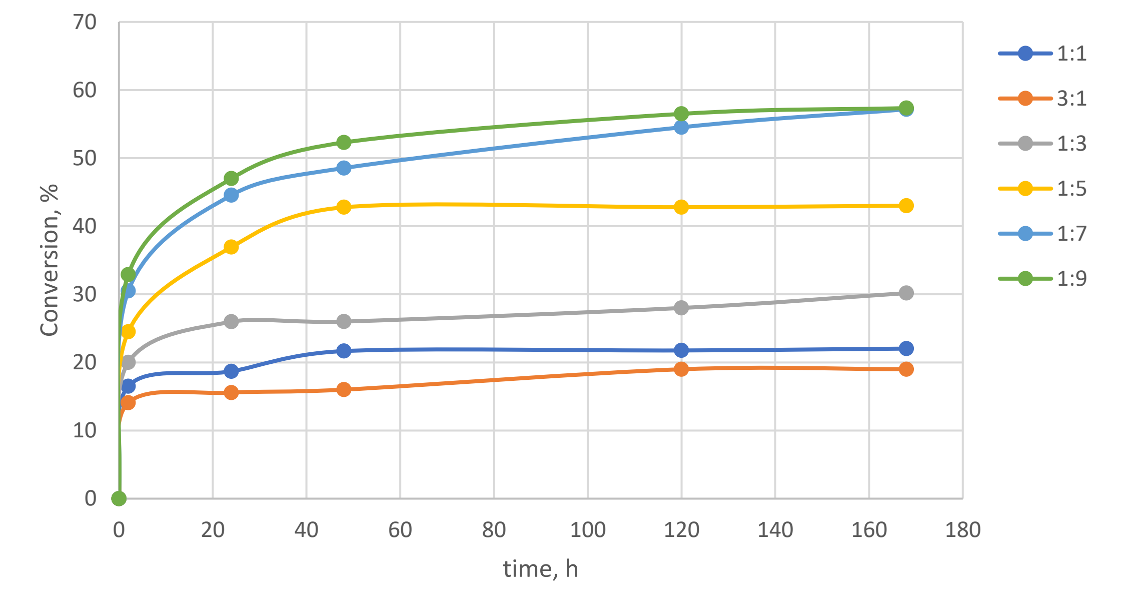

Figure SI-9. Dynamics of product **13** accumulation depending on the ratio base **5** to 3'-dIno. Conditions: reaction volume 1 ml, 50 °C, 9 mM potassium phosphate (pH 7.0), PNP 7 units/ml.

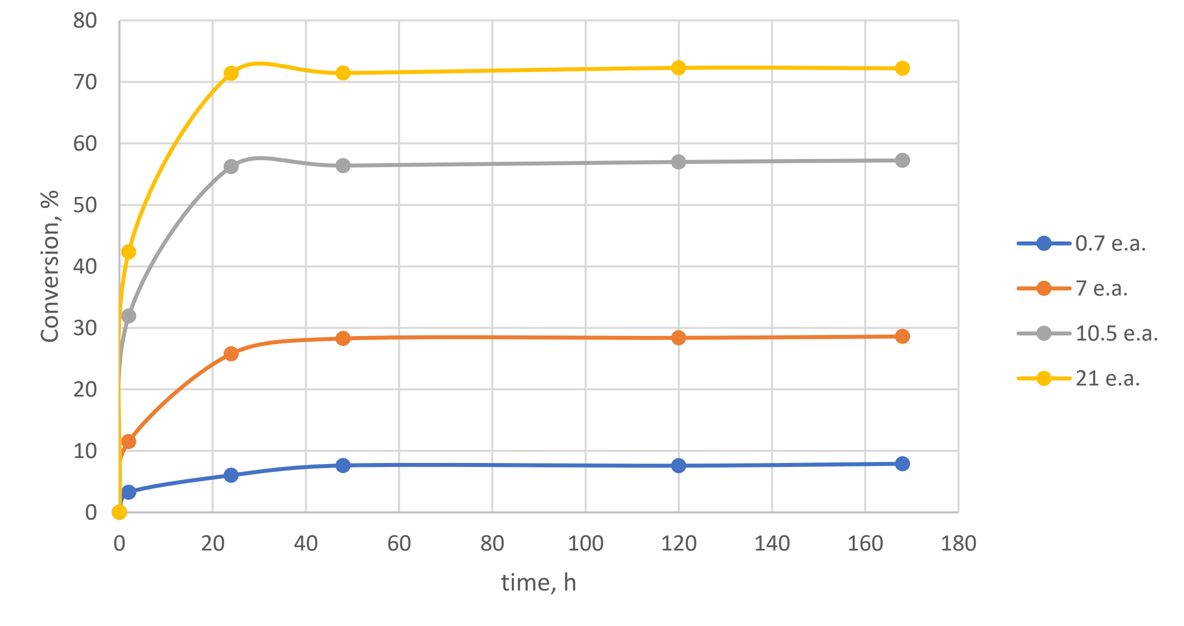

Figure SI-10. Dynamics of product **13** accumulation depending on PNP concentration. Conditions: base **5** to 3'-dIno ratio – 1:7; reaction volume 1 ml, 50 °C, 2 mM potassium phosphate (pH 7.0).

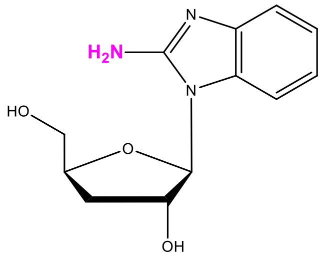

14

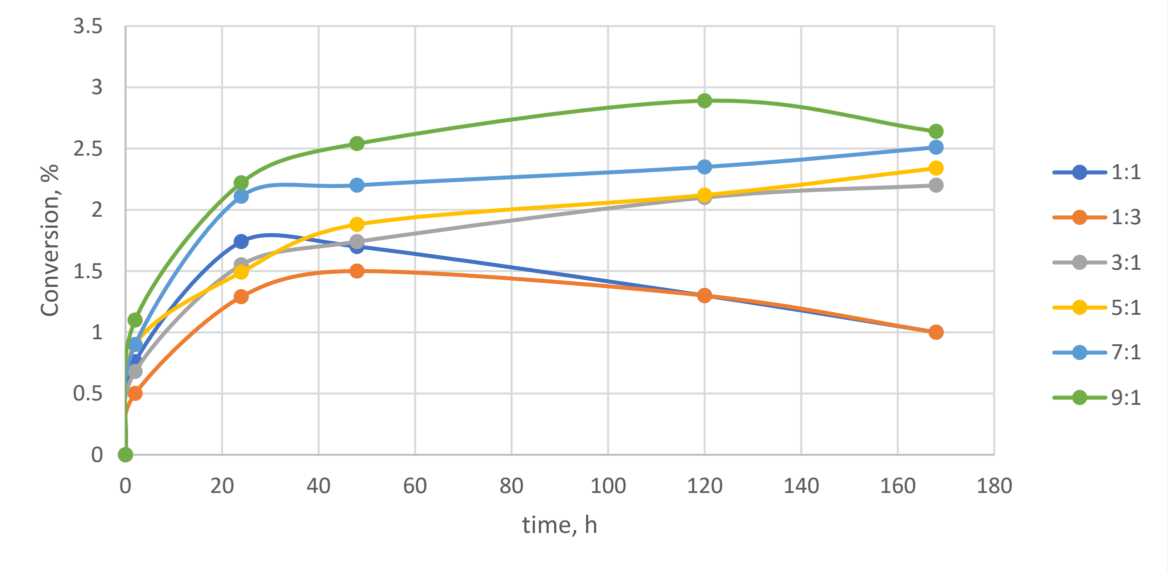

Figure SI-11. Dynamics of product **14** accumulation depending on the ratio base **6** to 3'-dIno. Conditions: reaction volume 1 ml, 50 °C, 2 mM potassium phosphate (pH 7.0), PNP 7 units/ml.

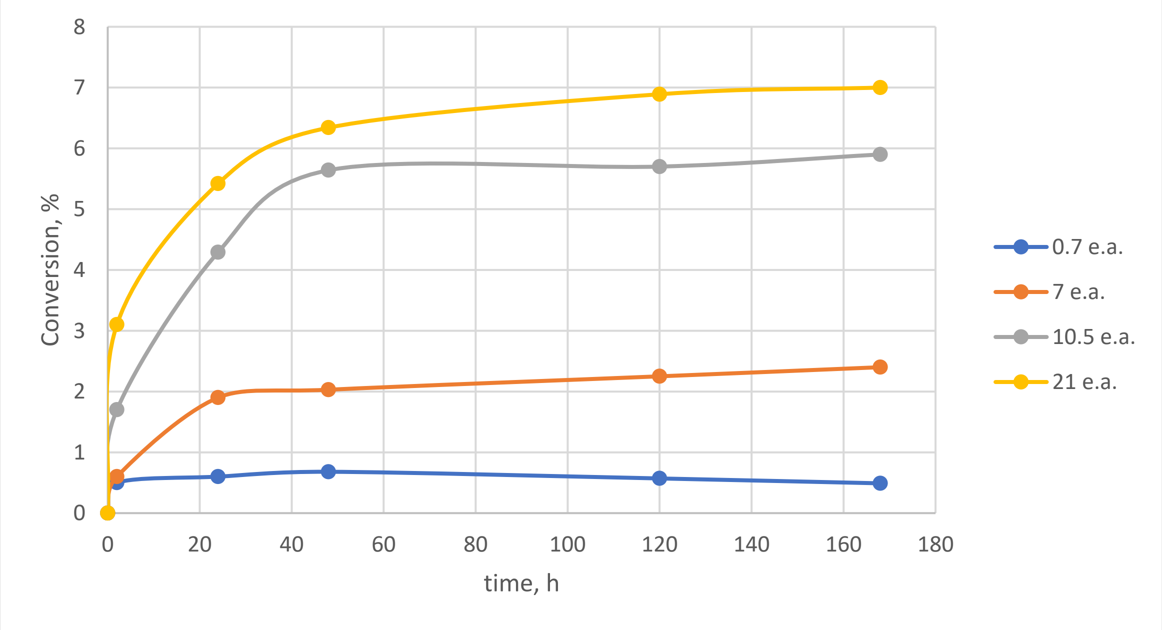

Figure SI-12. Dynamics of product **14** accumulation depending on PNP concentration. Conditions: base **6** to 3'-dIno ratio – 9:1; reaction volume 1 ml, 50 °C, 2 mM potassium phosphate (pH 7.0).

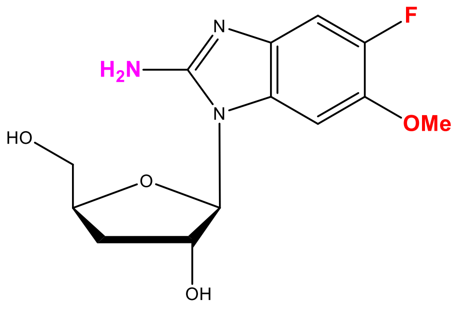

15

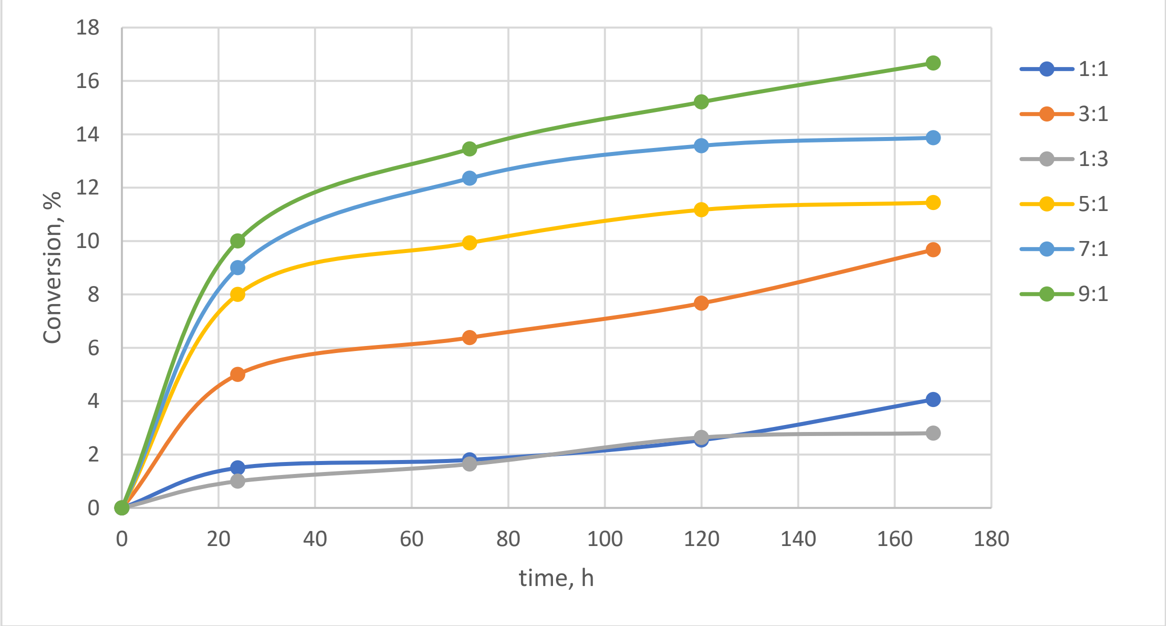

Figure SI-13. Dynamics of product **15** accumulation depending on the ratio base **7** to 3'-dIno. Conditions: reaction volume 1 ml, 50 °C, 2 mM potassium phosphate (pH 7.0), PNP 7 units/ml.

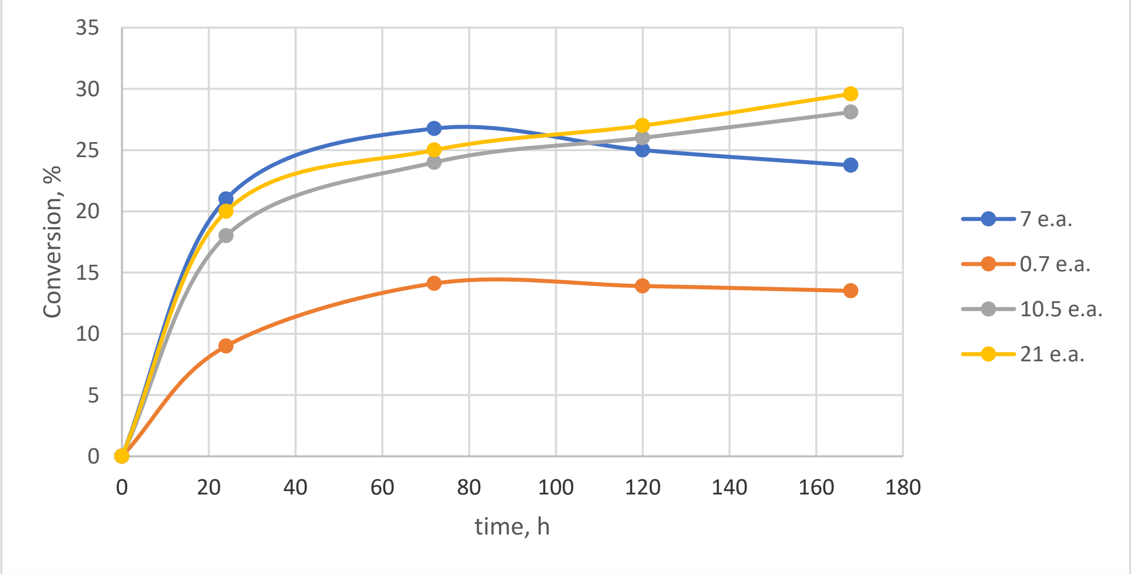

Figure SI-14. Dynamics of product **15** accumulation depending on PNP concentration. Conditions: base **7** to 3'-dIno ratio – 9:1; reaction volume 1 ml, 50 °C, 2 mM potassium phosphate (pH 7.0).

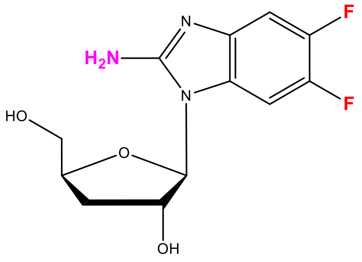

16

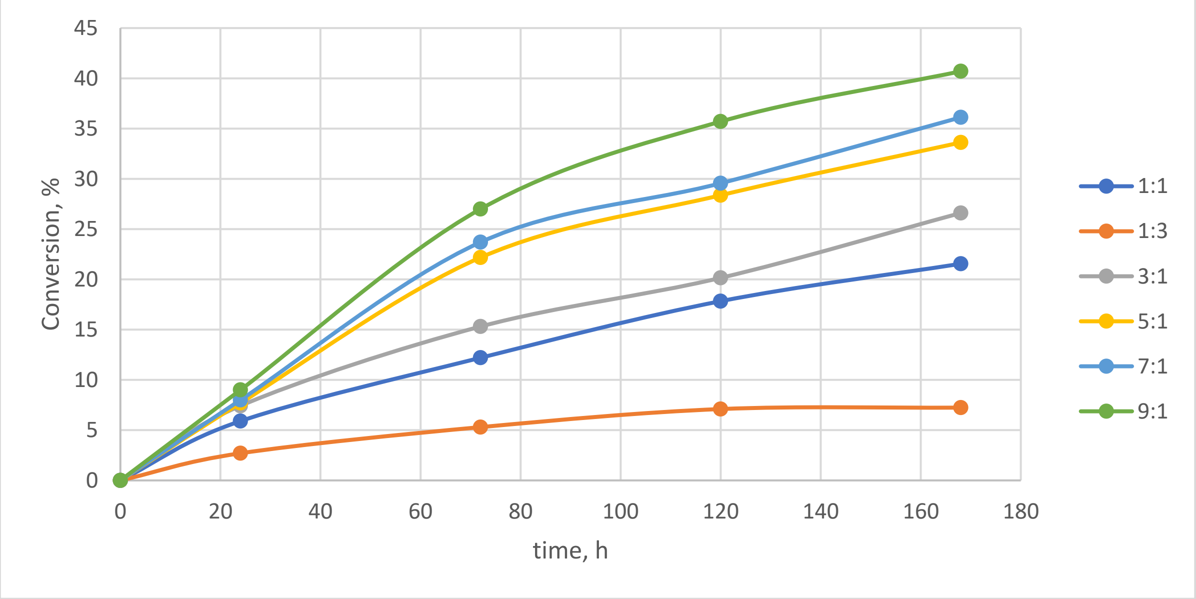

Figure SI-15. Dynamics of product **16** accumulation depending on the ratio base **8** to 3'-dIno. Conditions: reaction volume 1 ml, 50 °C, 2 mM potassium phosphate (pH 7.0), PNP 7 units/ml.

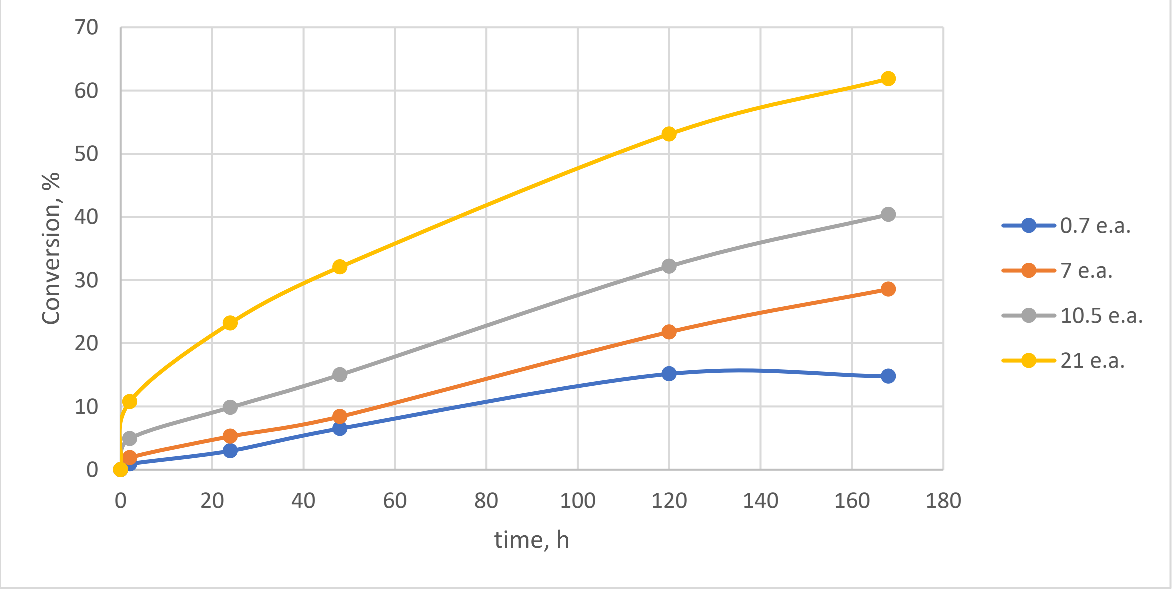

Figure SI-16. Dynamics of product **16** accumulation depending on PNP concentration. Conditions: base **7** to 3'-dIno ratio – 9:1; reaction volume 1 ml, 50 °C, 2 mM potassium phosphate (pH 7.0).

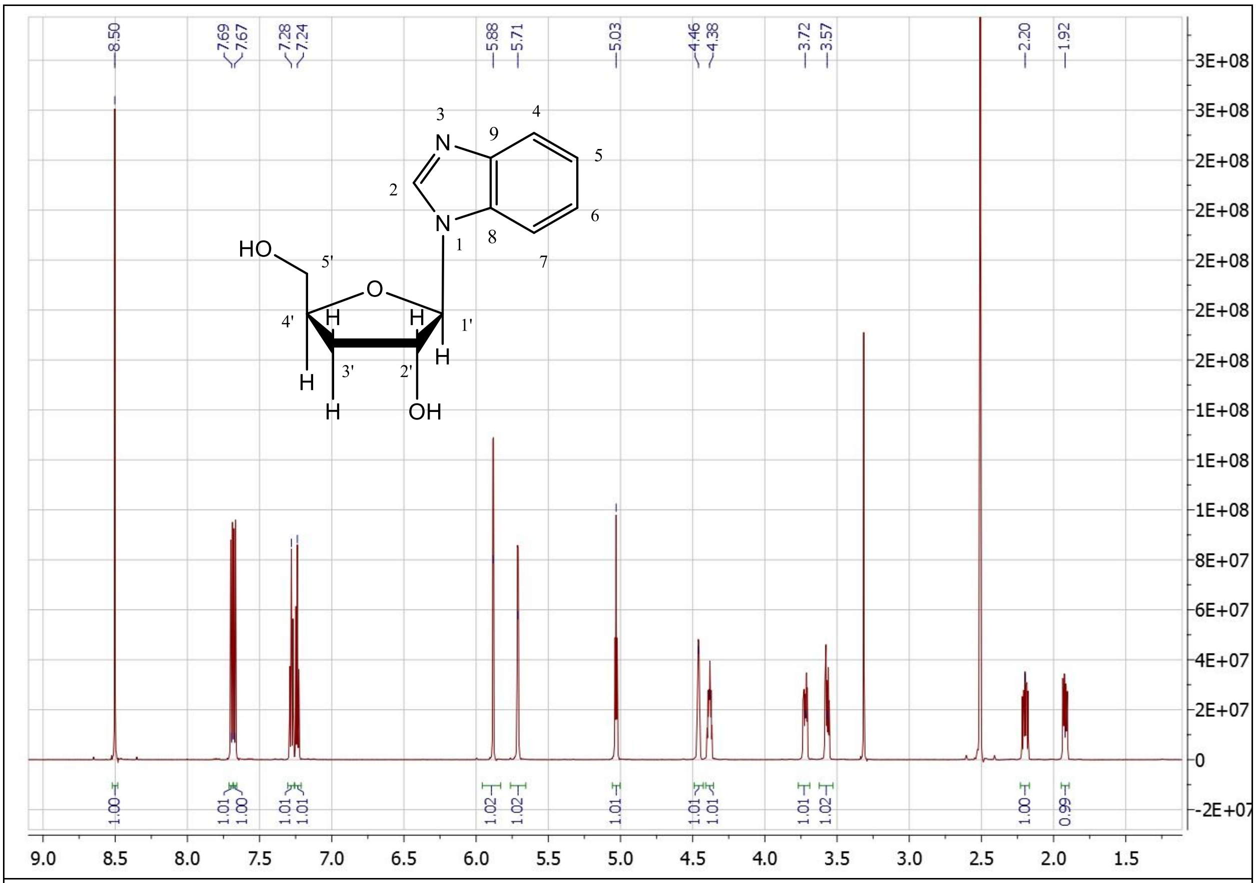

Figure SI-17. The ^{1}H NMR spectrum of 1-(β-D-3'-deoxyribofuranosyl)benzimidazole **9.**

^{1}H NMR (700 MHz, DMSO-d6): 8.50 (s, 1H, H-2), 7.69 (d, *J* = 8.0, 1H, H-7), 7.67 (d, *J* = 7.8, 1H, H-4), 7.30 - 7.26 (m, 1H, H-5), 7.26 - 7.22 (m, 1H, H-6), 5.88 (d, *J* = 2.4, 1H, H-1'), 5.71 (d, *J* = 3.5, 1H, 2'-OH), 5.02 – 5.06 (m, 1H, 5'-OH), 4.47-4.43 (m, 1H, H2'), 4.41-4.36 (m, 1H, H4'), 3.74 - 3.69 (m, 1H, H-5'), 3.58 -3.54 (m, 1H, H-5'), 2.23 - 2.17 (m, 1H, H-3'), 1.92 (ddd, *J* = 9.2; 6.4; 3.0, 1H, H-3').

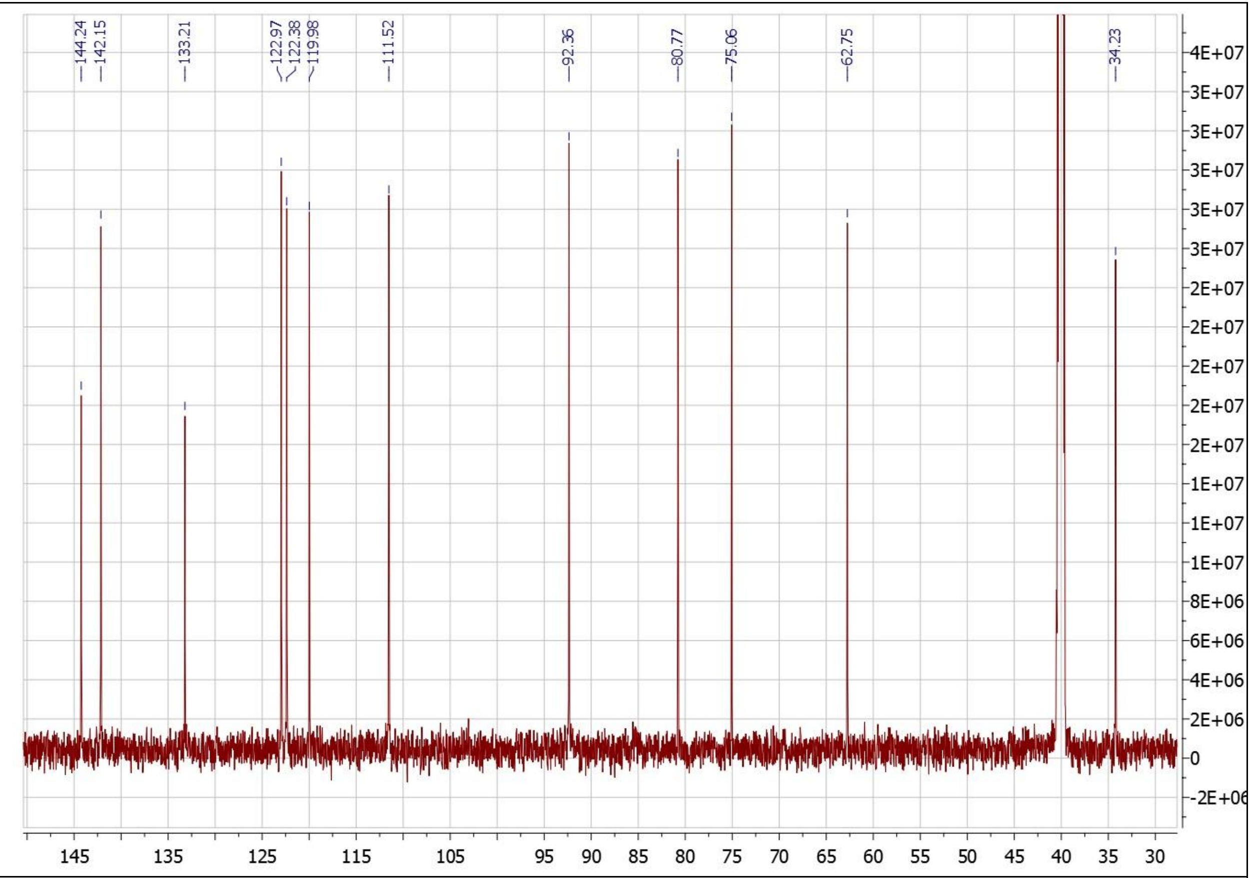

Figure SI-18. The ^{13}C NMR spectrum of 1-(β-D-3'-deoxyribofuranosyl)benzimidazole **9.**

^{13}C NMR (176 MHz, DMSO-d6): 144.24 (C9), 142.15 (C2), 133.21 (C8), 122.97 (C5), 122.38 (C6), 119.98 (C7), 111.52 (C4), 92.36 (C1'), 80.77 (C4'), 75.06 (C2'), 62.75 (C5'), 34.23 (C3').

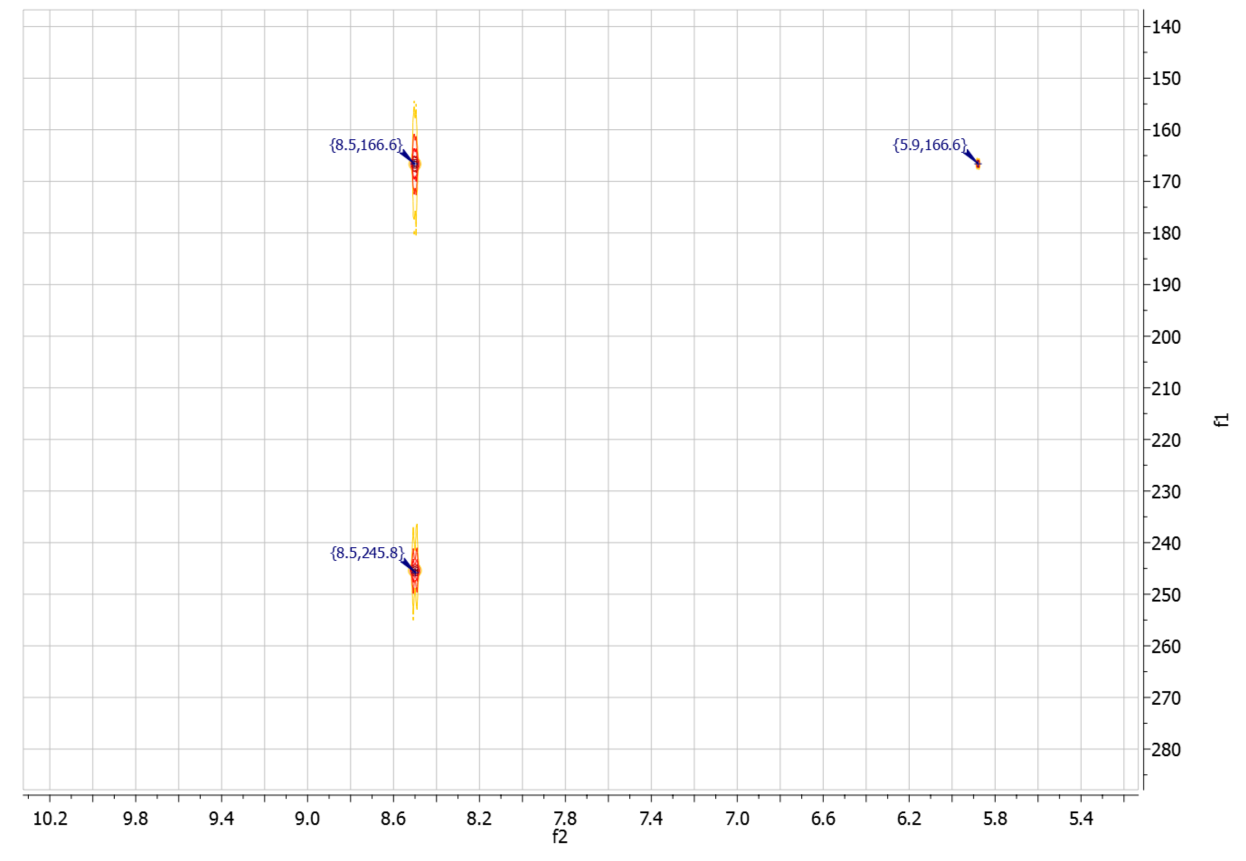

Figure SI-19. The ^{1}H-^{15}N HMBC NMR spectrum of 1-(β-D-3'-deoxyribofuranosyl)benzimidazole **9.**

^{15}N NMR (71 MHz, DMSO-d6): 245.8 (N3), 166.6 (N1).

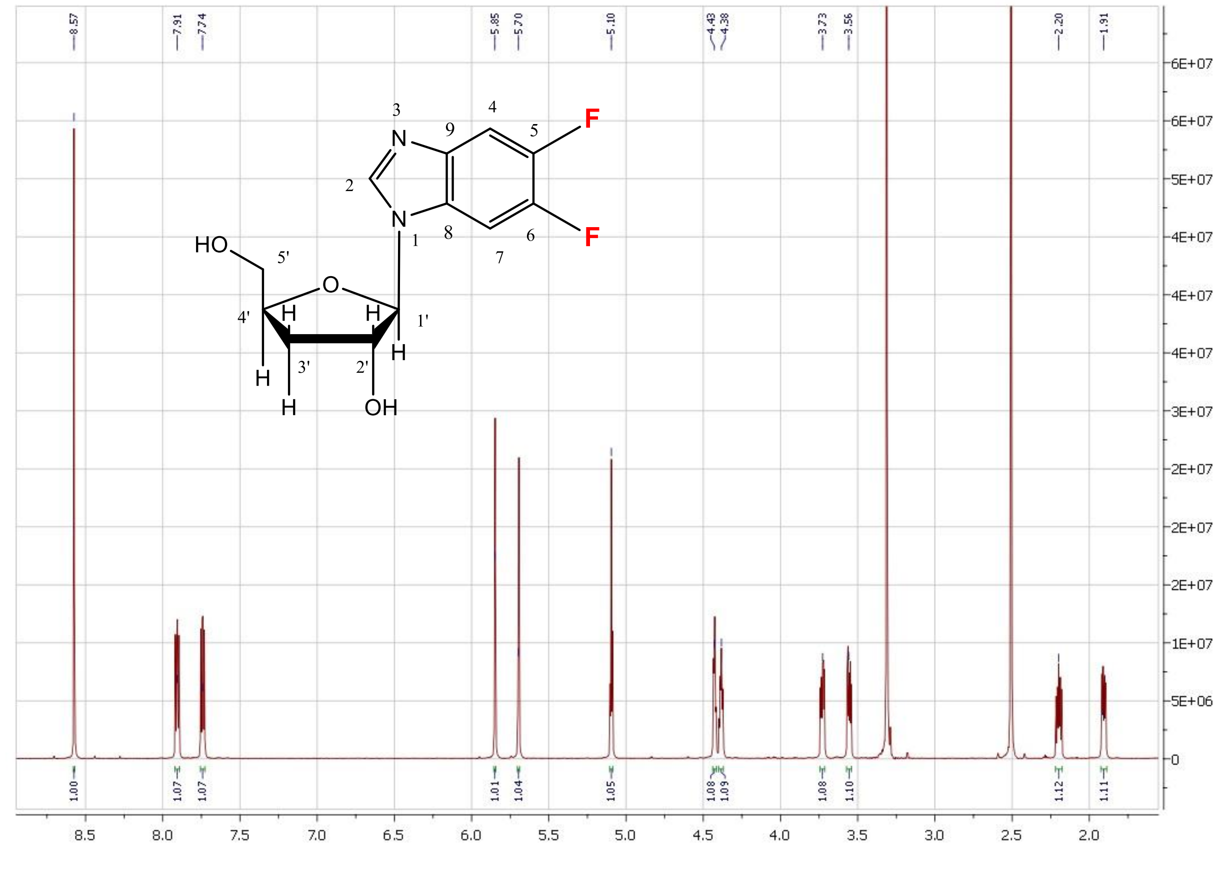

Figure SI-20. The ^{1}H NMR spectrum of 5,6-difluoro-1-(β-D-3'-deoxyribofuranosyl)benzimidazole **10.**
^{1}H NMR (700 MHz, DMSO-d6): 8.57 (s, 1H, H-2), 7.91 (dd, *J* = 10.8; 7.4, 1H, H-7), 7.74 (dd, *J* = 11.0; 7.5, 1H, H-4), 5.85 (d, *J* = 2.7, 1H, H-1'), 5.70 (d, *J* = 3.9, 1H, 2'-OH), 5.12 – 5.08 (m, 1H, 5'-OH), 4.45-4.41 (m, 1H, H2'), 4.40-4.36 (m, 1H, H4'), 3.75 - 3.70 (m, 1H, H-5'). 3.59 -3.53 (m, 1H, H-5'), 2.23-2.16 (m, 1H, H-3'), 1.94-1.88 (m, 1H, H-3').

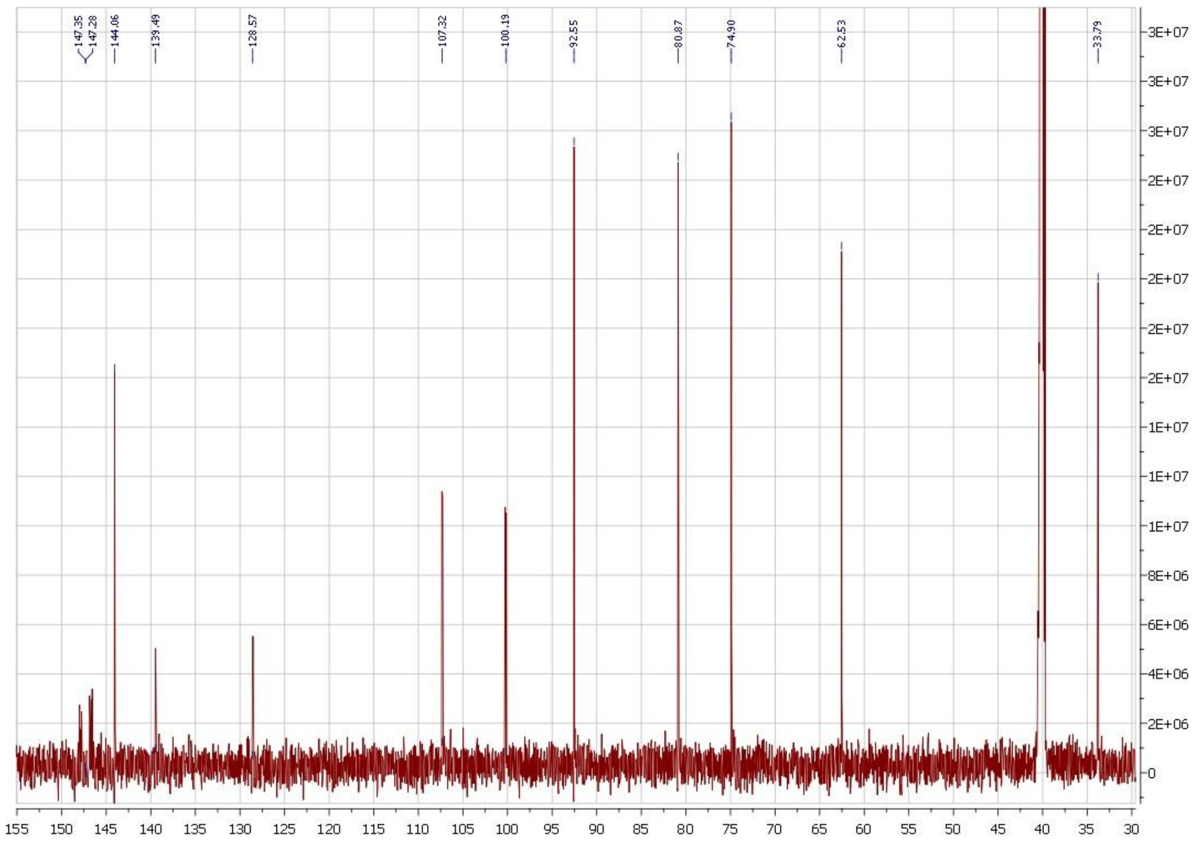

Figure SI-21. The ^{13}C NMR spectrum of 5,6-difluoro-1-(β-D-3'-deoxyribofuranosyl)benzimidazole **10.** ^{13}C NMR (176 MHz, DMSO-d6): 147.35 (dd, *J* = 239.7; 44.8, C5), 146.28 (dd, *J* = 232.4; 44.8, C6), 144.06 (s, C2), 139.49 (d, *J* = 10.8, C9), 128.57 (d, *J* = 11.2, C8), 107.32 (s, C4), 100.19 (d, *J* = 23.5, C7), 92.55 (s, C1'), 80.87 (s, C4'), 74.90 (s, C2'), 62.53 (s, C5'), 33.79 (s, C3').

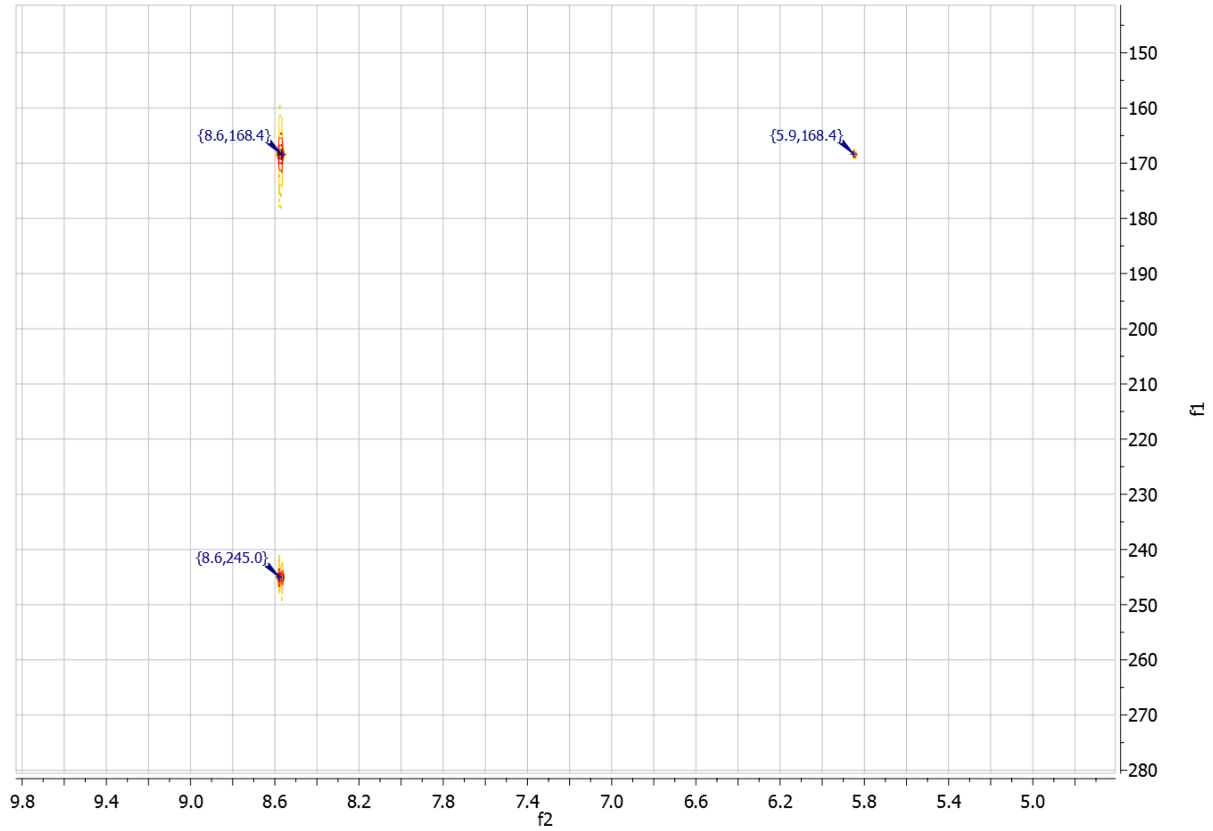

Figure SI-22. The ^{1}H-^{15}N HMBC NMR spectrum of 5,6-difluoro-1-(β-D-3'-deoxyribofuranosyl)benzimidazole **10.**

^{15}N NMR (71 MHz, DMSO-d6) : 245.0 (N3), 168.4 (N1).

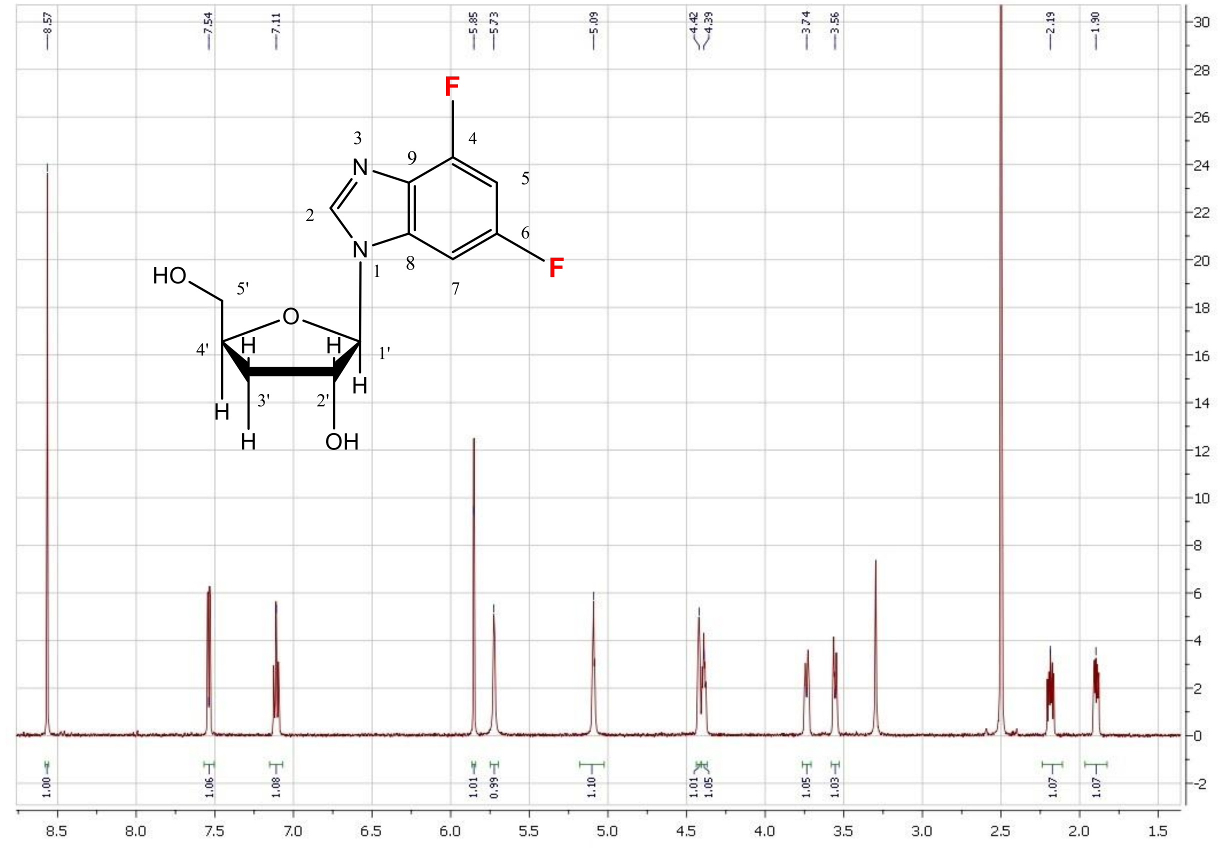

Figure SI-23. The ^{1}H NMR spectrum of 4,6-difluoro-1-(β-D-3'-deoxyribofuranosyl)benzimidazole **11a** (N1-isomer).

^{1}H NMR (700 MHz, DMSO-d6): 8.57 (s, 1H, H-2), 7.54 (dd, *J* = 9.1; 2.1, 1H, H-7), 7.11 (dt, *J* = 10.8; 2.1, 1H, H-5), 5.85 (d, *J* = 2.2, 1H, H-1'), 5.73 (br. s, 1H, 2'-OH), 5.11 – 5.06 (m, 1H, 5'-OH), 4.44 – 4.41 (m, 1H, H-2'), 4.41 - 4.37 (m, 1H, H-4'), 3.76 – 3.71 (m, 1H, H-5'), 3.58 – 3.53 (m, 1H, H-5'), 2.21 - 2.16 (m, 1H, H-3'), 1.92 - 1.87 (m, 1H, H-3').

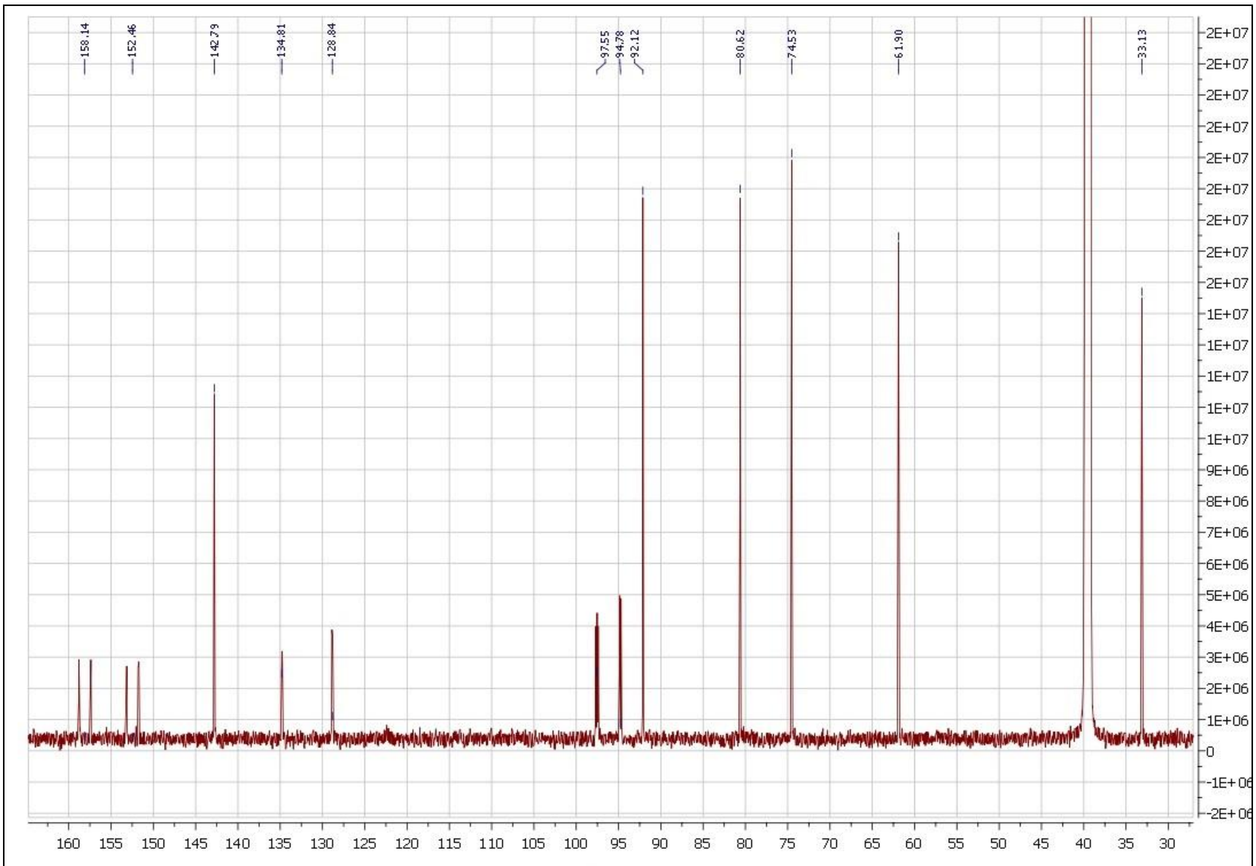

Figure SI-24. The ^{13}C NMR spectrum of 4,6-difluoro-1-(β-D-3'-deoxyribofuranosyl)benzimidazole **11a** (N1-isomer).

^{13}C NMR (176 MHz, DMSO-d6): 158.14 (dd, *J* = 238.5; 11.2, C6), 152.46 (dd, *J* = 253.1; 15.3, C4), 142.79 (s, C2), 134.81 (dd, *J* = 16.0; 11.1, C8), 128.84 (d, *J* = 16.4, C9), 97.55 (dd, *J* = 29.5; 22.0, C5), 94.78 (dd, *J* = 28.0; 4.0, C7), 92.12 (s, C1'), 80.62 (s, C4'), 74.53 (s, C2'), 61.90 (s, C5'), 33.13 (s, C3').

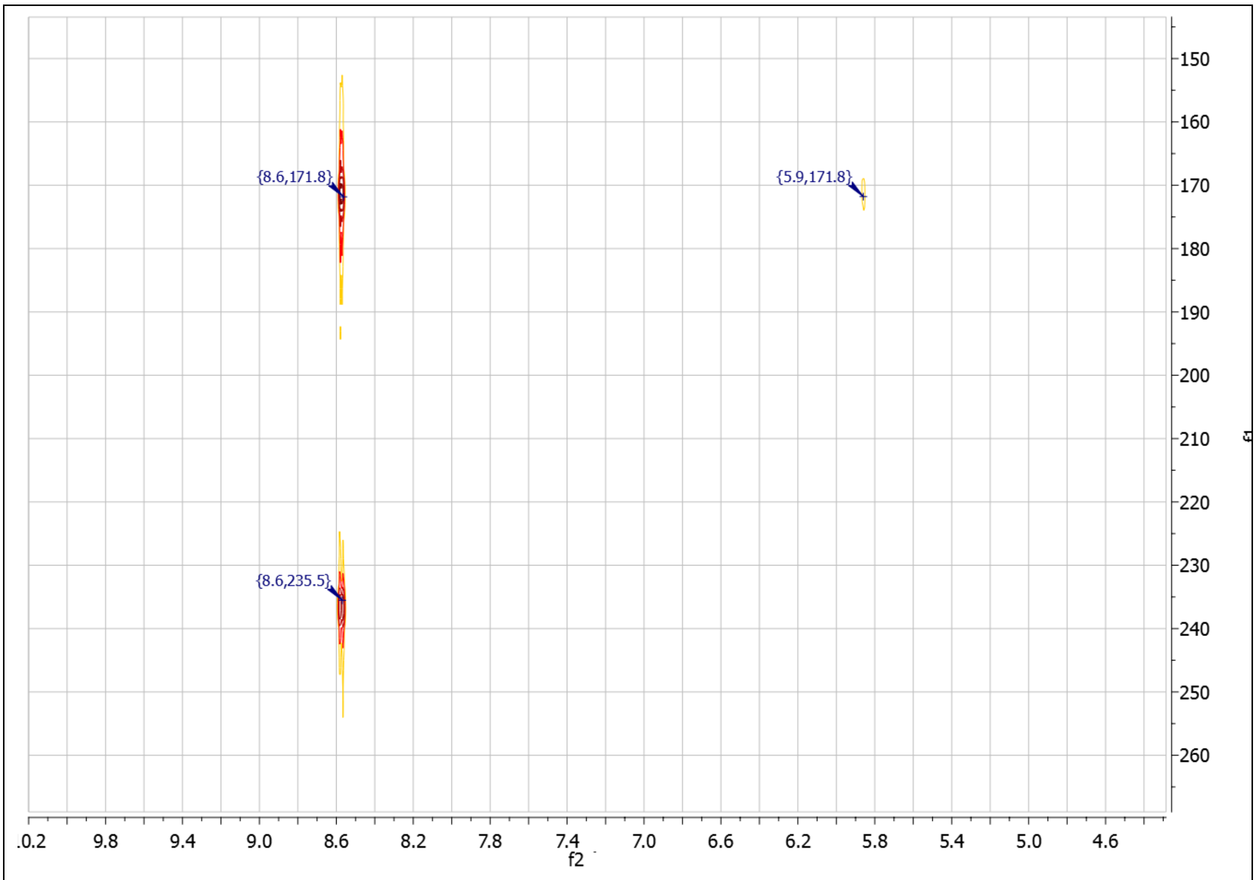

Figure SI-25. The ^{1}H-^{15}N HMBC NMR spectrum of 4,6-difluoro-1-(β-D-3'-deoxyribofuranosyl)benzimidazole **11a** (N1-isomer).
^{15}N NMR (71 MHz, DMSO-d6):235.5 (N3), 171.8 (N1).

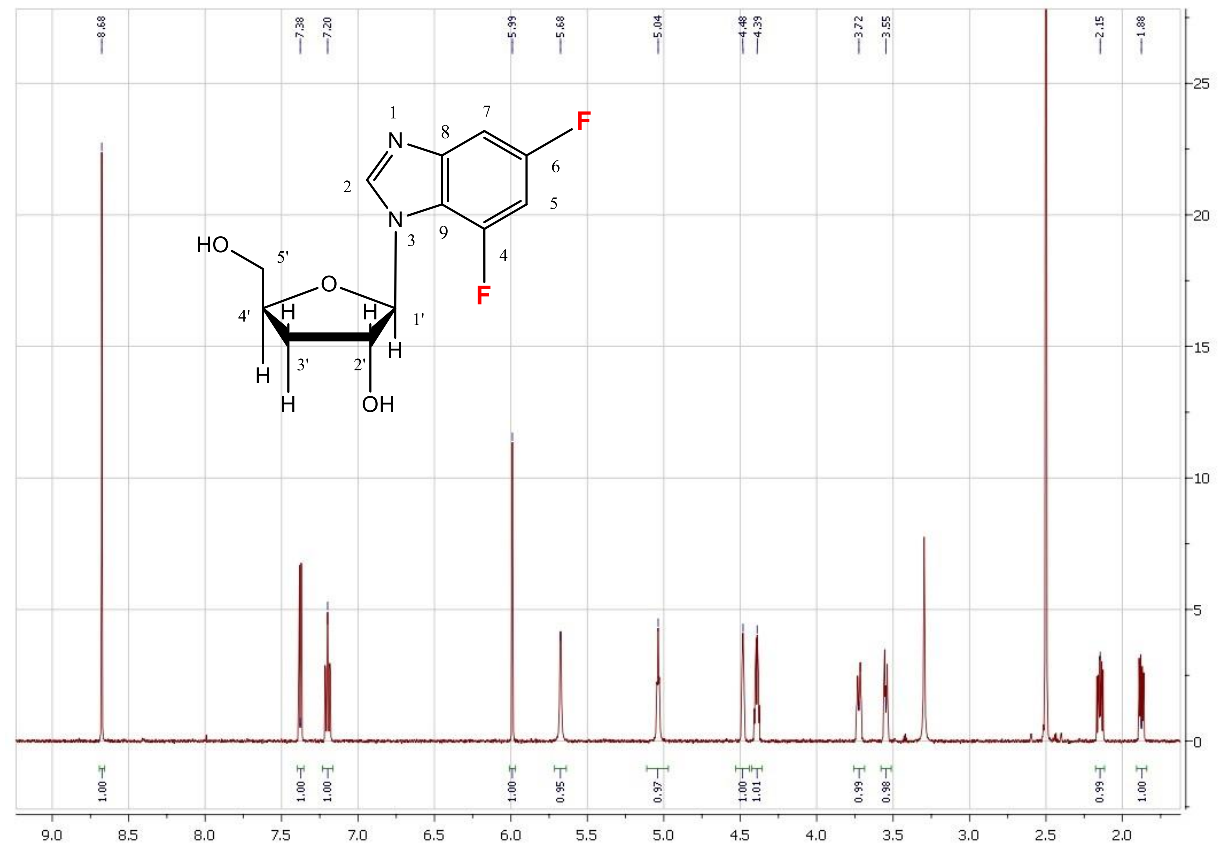

Figure SI-26. The ^{1}H NMR spectrum of 4,6-difluoro-1-(β-D-3'-deoxyribofuranosyl)benzimidazole **11b** (N3-isomer).

^{1}H NMR (700 MHz, DMSO-d6): 8.68 (s, 1H, H-2), 7.38 (dd, *J* = 9.2; 2.3, 1H, H-7), 7.20 (dt, *J* = 11.7; 2.2, 1H, H-5), 6.00-5.98 (br s, 1H, H-1'), 5.68 (d, *J* = 3.8, 1H, 2'-OH), 5.07 – 4.99 (m, 1H, 5'-OH), 4.50 – 4.47 (m, 1H, H-2'), 4.41 - 4.37 (m, 1H, H-4'), 3.74 – 3.70 (m, 1H, H-5'), 3.57 – 3.53 (m, 1H, H-5'), 2.18 - 2.12 (m, 1H, H-3'), 1.91 - 1.84 (m, 1H, H-3').

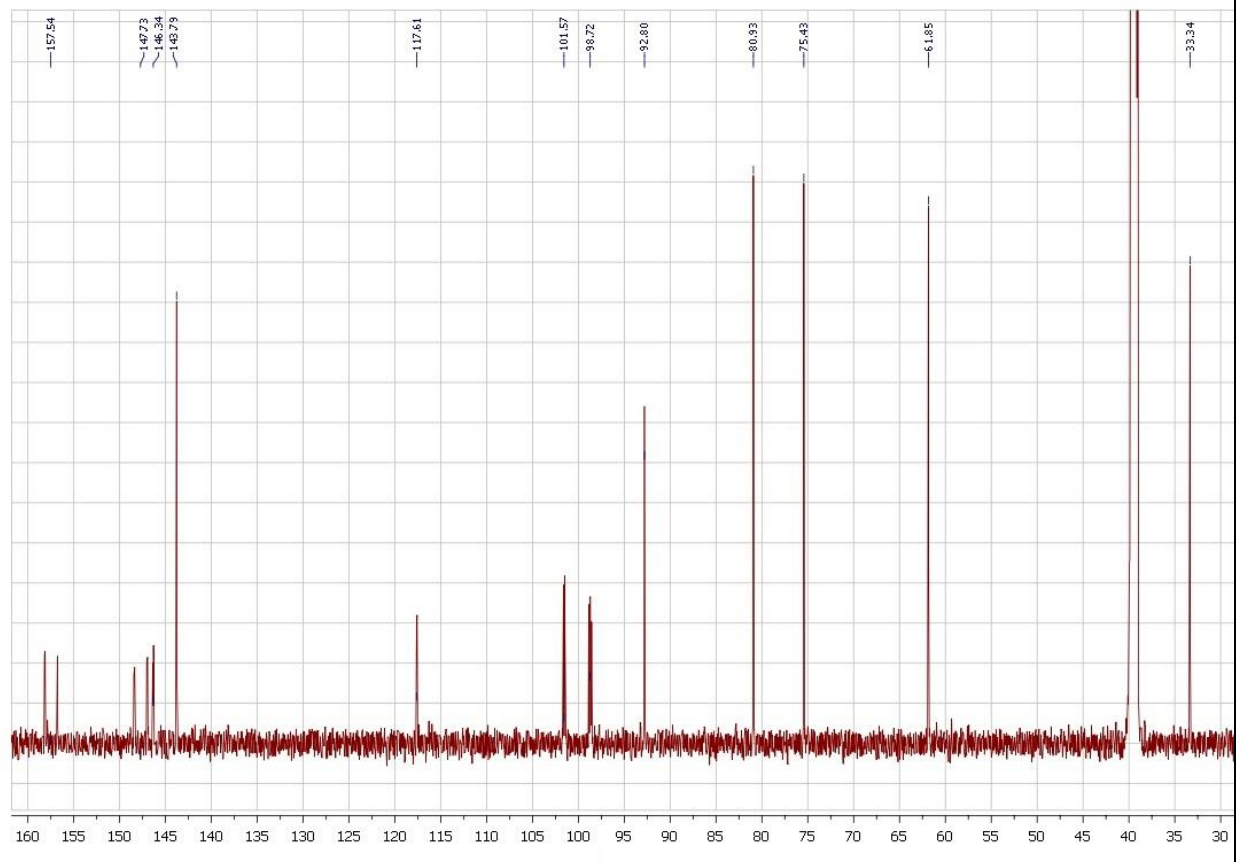

Figure SI-27. The ^{13}C NMR spectrum of 4,6-difluoro-1-(β-D-3'-deoxyribofuranosyl)benzimidazole **11b** (N3-isomer).

^{13}C NMR (176 MHz, DMSO-d6): 157.54 (dd, *J* = 237.0; 11.0, C6), 147.73 (dd, *J* = 248.7; 15.5, C4), 146.34 (dd, *J* = 14.4; 5.5, C8), 143.79 (s, C2), 117.61 (d, *J* = 10.3, C9), 101.57 (dd, *J* = 23.9; 4.1, C7), 98.72 (dd, *J* = 30.5; 22.6, C5), 92.80 (d, *J* = 3.6, C1'), 80.93 (C4'), 75.43 (C2'), 61.85 (C5'), 33.34 (C3').

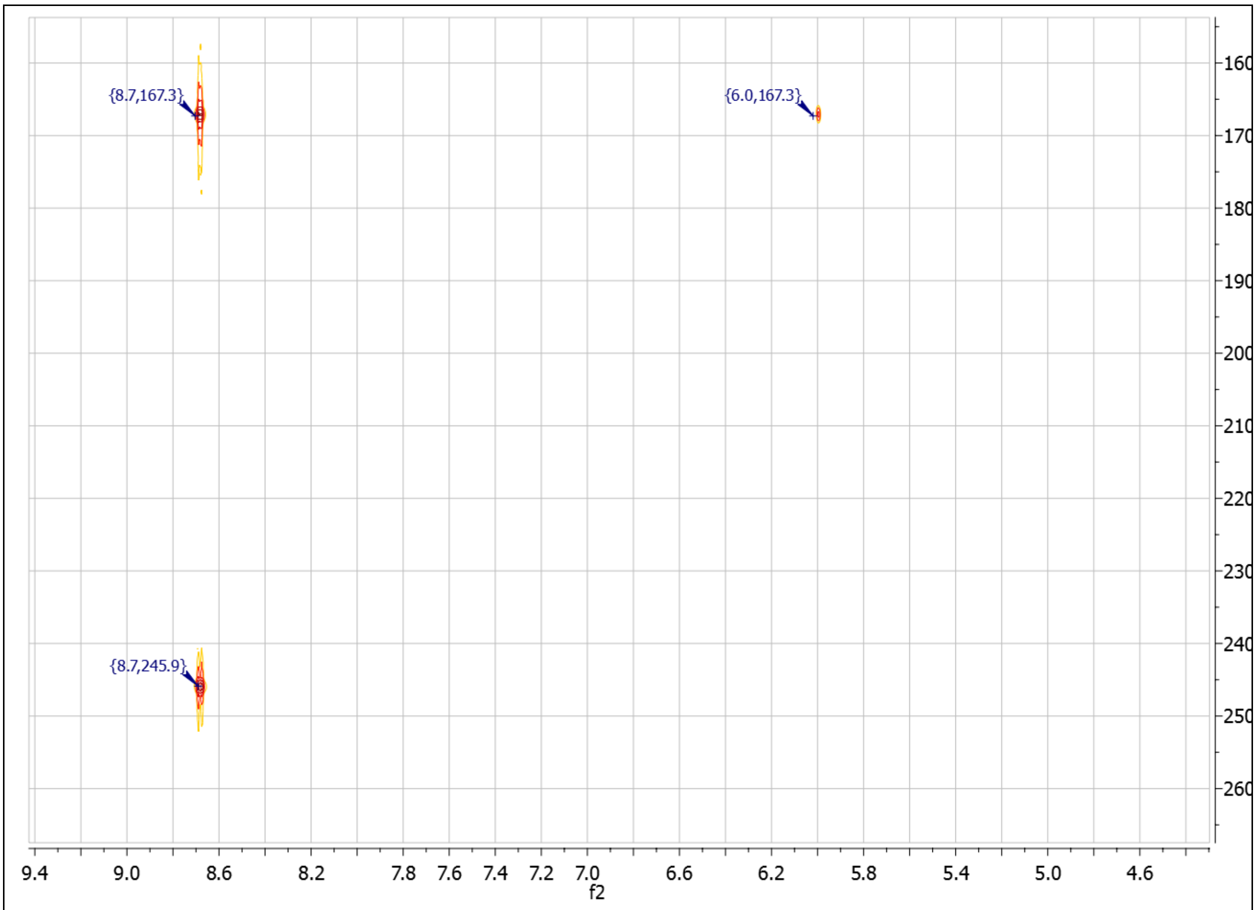

Figure SI-28. The ^{1}H-^{15}N HMBC NMR spectrum of 4,6-difluoro-1-(β-D-3'-deoxyribofuranosyl)benzimidazole **11b** (N3-isomer).
^{15}N NMR (71 MHz, DMSO-d6): 245.9 (N3), 167.3 (N1).

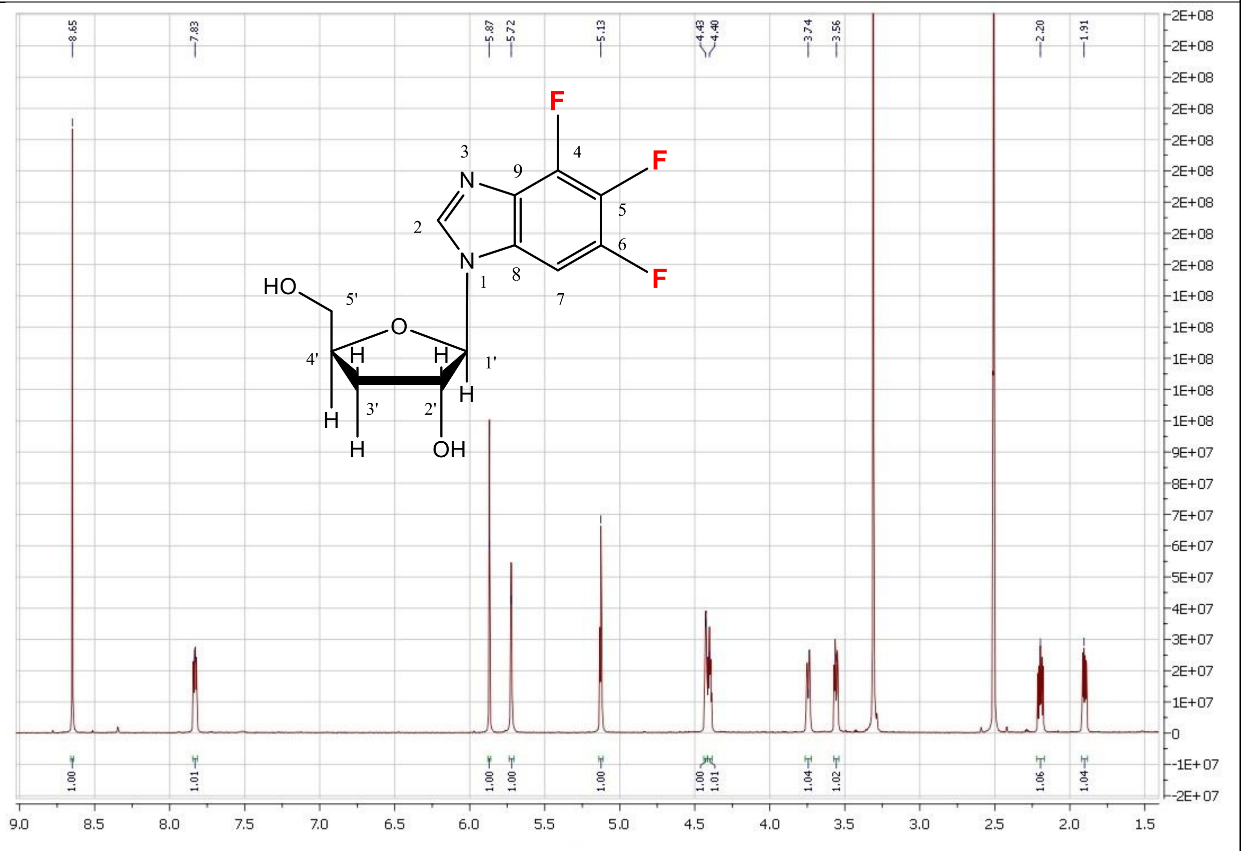

Figure SI-29. The ^{1}H NMR spectrum of 4,5,6-trifluoro-1-(β-D-3'-deoxyribofuranosyl)benzimidazole **12a** (N1-isomer).

^{1}H NMR (700 MHz, DMSO-d6): 8.65 (s, 1H, H-2), 7.85 - 7.82 (m, 1H, H-7), 5.87 (d, *J* = 2.3, 1H, H-1'), 5.72 (d, *J* = 3.4, 1H, 2'-OH), 5.15 – 5.08 (m, 1H, 5'-OH), 4.44-4.42 (m, 1H, H2'), 4.41-4.39 (m, 1H, H4'), 3.77 - 3.72 (m, 1H, H-5'). 3.58 -3.53 (m, 1H, H-5'), 2.22-2.17 (m, 1H, H-3'), 1.93-1.88 (m, 1H, H-3').

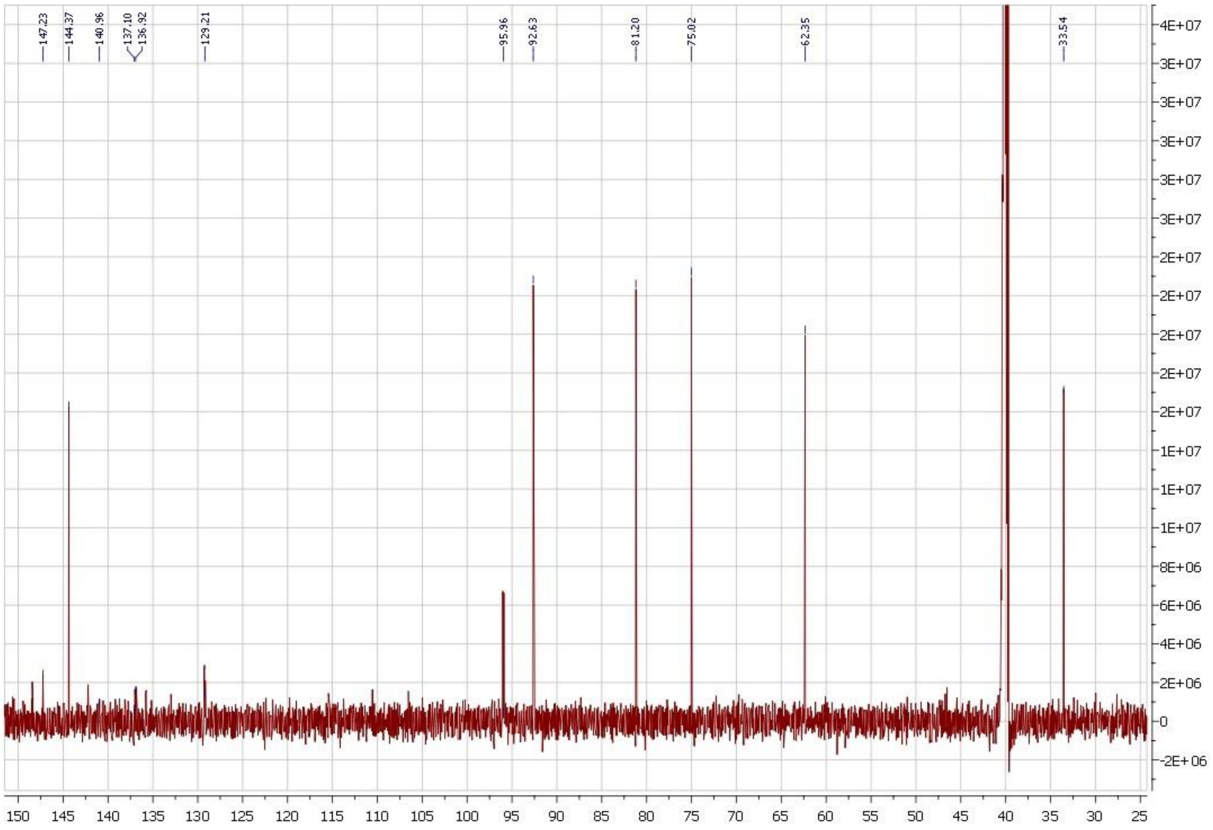

Figure SI-30. The ^{13}C NMR spectrum of 4,5,6-trifluoro-1-(β-D-3'-deoxyribofuranosyl)benzimidazole **12a** (N1-isomer).

^{13}C NMR (176 MHz, DMSO-d6): 148.59 - 147.15 (m, C6), 144.37 (d, *J* = 2.2, C2), 141.07 - 140.85 (m, C4), 137.16 - 136.73 (m, C9), 137.01 - 136.76 (m, C5), 129.42 - 129.06 (m, C8), 95.96 (d, *J* = 24.1, C7), 92.63 (s, C1'), 81.20 (s, C4'), 75.02 (s, C2'), 62.35 (s, C5'), 33.54 (s, C3').

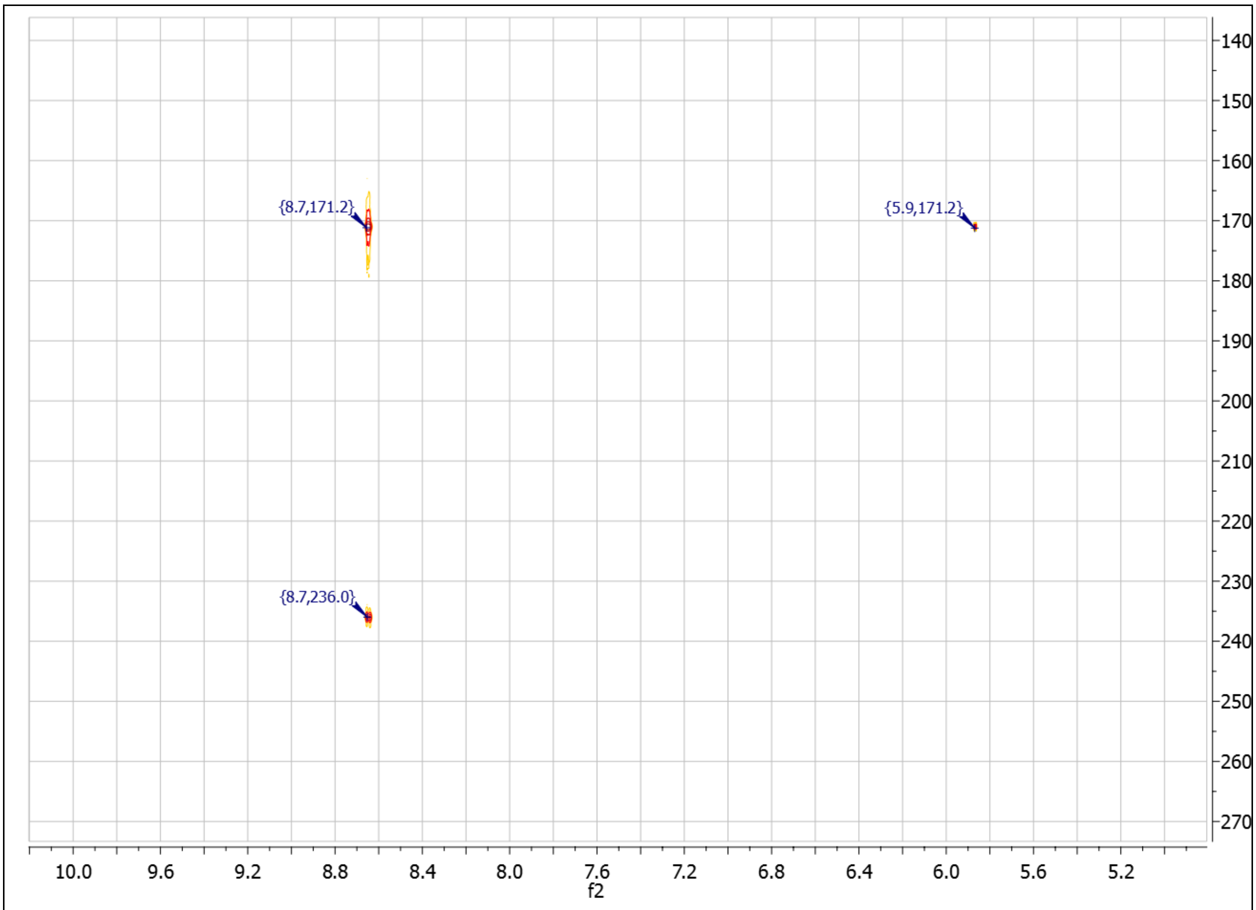

Figure SI-31. The ^{1}H-^{15}N HMBC NMR spectrum of 4,5,6-trifluoro-1-(β-D-3'-deoxyribofuranosyl)benzimidazole **12a** (N1-isomer).
^{15}N NMR (71 MHz, DMSO-d6): 236.0 (N3),171.2 (N1).

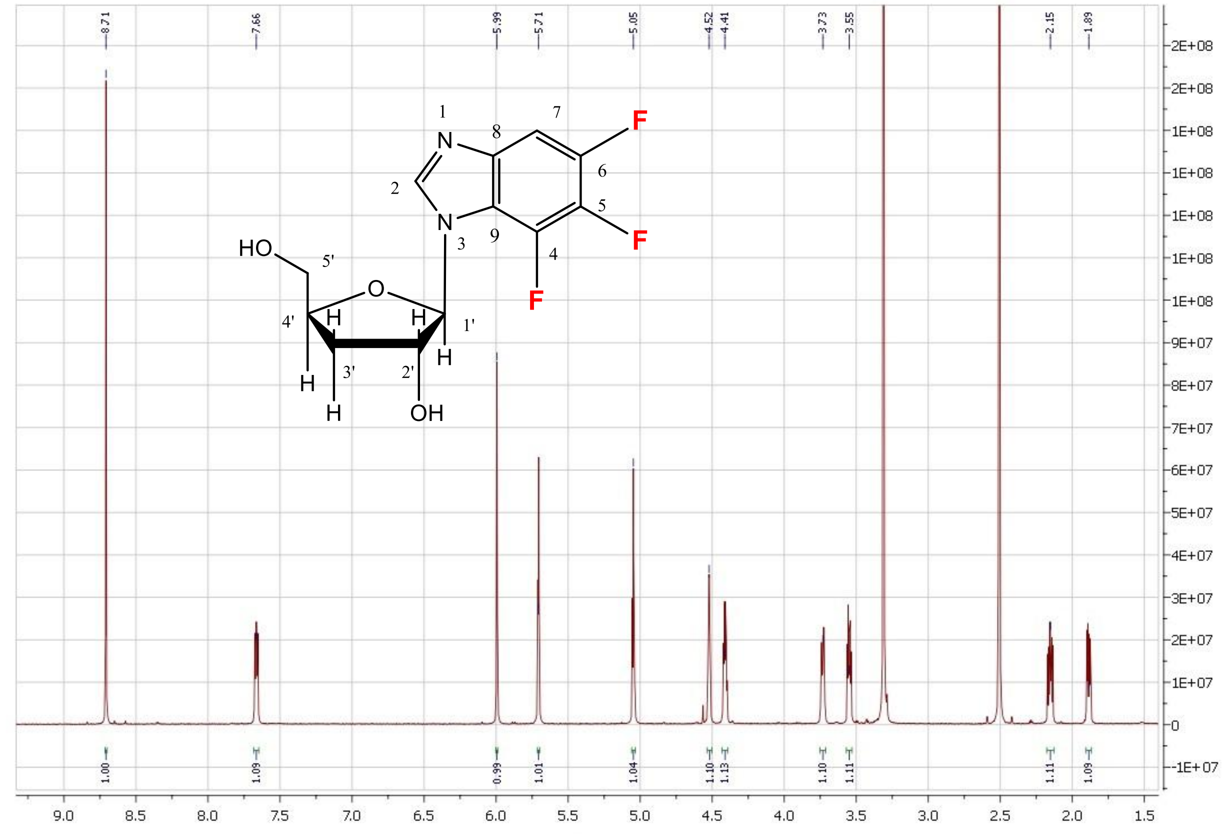

Figure SI-32. The ^{1}H NMR spectrum of 4,5,6-trifluoro-1-(β-D-3'-deoxyribofuranosyl)benzimidazole **12b** (N3-isomer).

^{1}H NMR (700 MHz, DMSO-d6): 8.71 (s, 1H, H-2), 7.68 - 7.65 (m, 1H, H-7), 5.99 (br. s, 1H, H-1'), 5.71 (d, *J* = 4.4, 1H, 2'-OH), 5.057 – 4.99 (m, 1H, 5'-OH), 4.52 (br. s, 1H, H-2'), 4.44-4.39 (m, 1H, H-4'), 3.75-3.70 (m, 1H, H-5'), 3.57-3.52 (m, 1H, H-5'), 2.18-2.12 (m, 1H, H-3'), 1.91-1.86 (m, 1H, H-3').

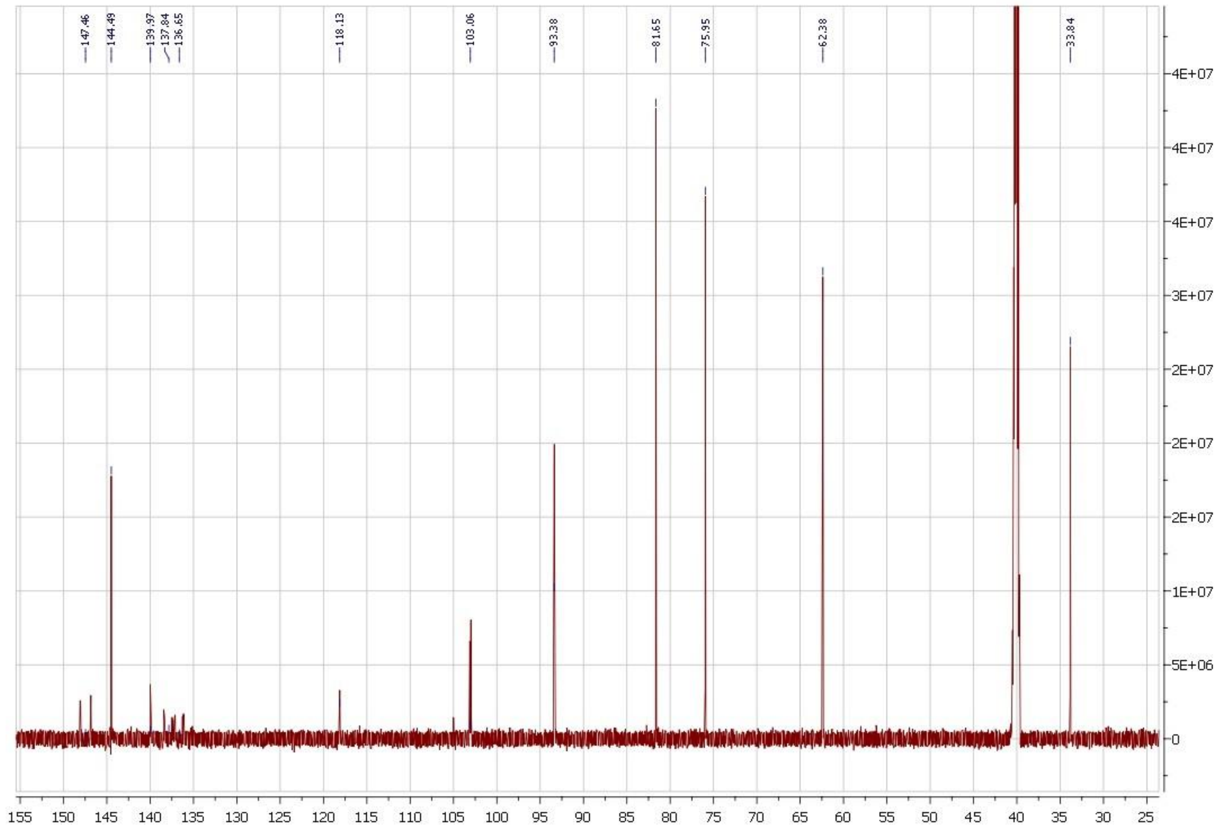

Figure SI-33. The ^{13}C NMR spectrum of 4,5,6-trifluoro-1-(β-D-3'-deoxyribofuranosyl)benzimidazole **12b** (N3-isomer).

^{13}C NMR (176 MHz, DMSO-d6): 147.46 (dd, *J* = 240.1; 12.5, C6), 144.49 (d, *J* = 1.9, C2), 140.02 - 139.90 (m, C8), 138.51 - 137.04 (m, C4), 137.11 - 136.16 (m, C5), 118.18 - 118.09 (m, C9), 103.06 (ddd, *J* = 19.9; 3.8; 1.9, C7), 93.38 (d, *J* = 3.3, C1'), 81.65 (s, C4'), 75.95 (s, C2'), 62.38 (s, C5'), 33.84 (s, C3').

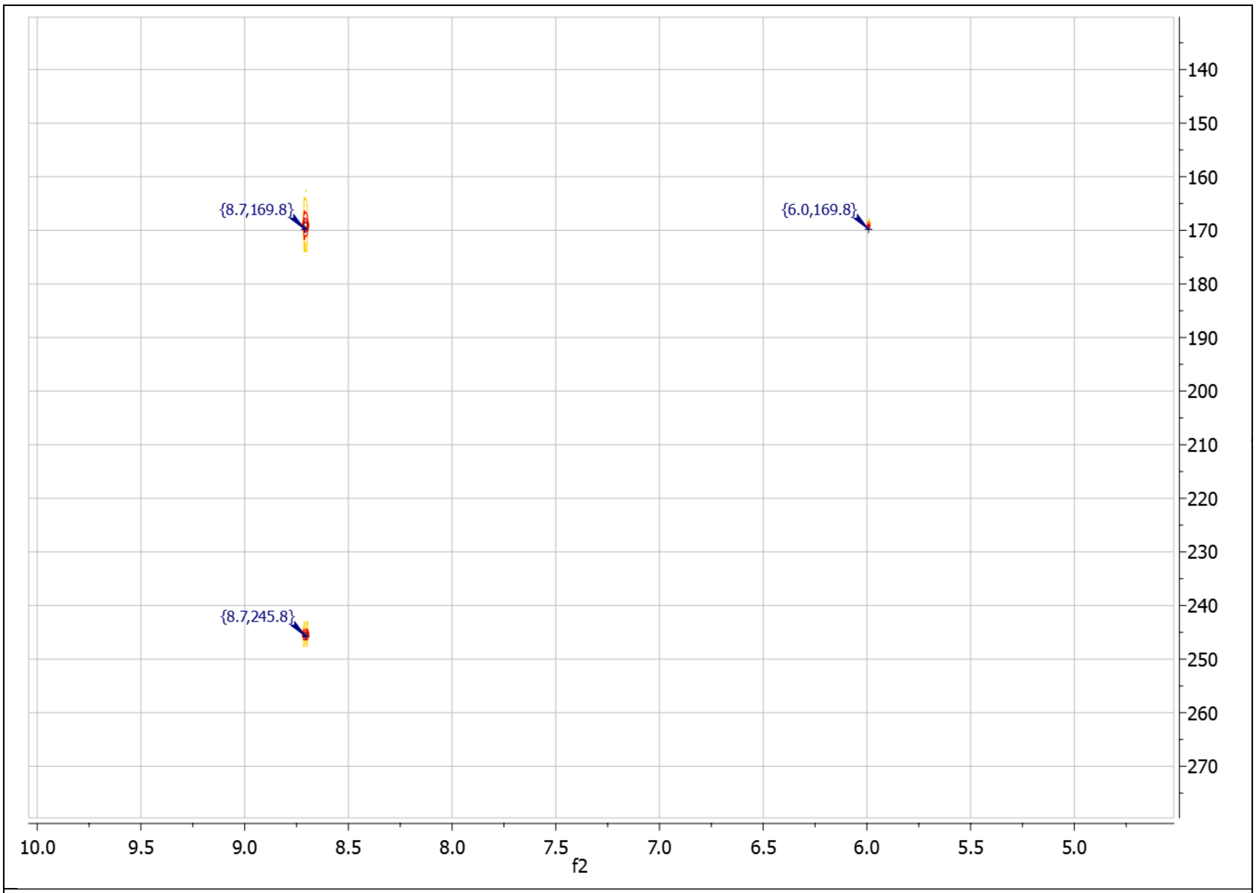

Figure SI-34. The ^{1}H-^{15}N HMBC NMR spectrum of 4,5,6-trifluoro-1-(β-D-3'-deoxyribofuranosyl)benzimidazole **12b** (N3-isomer).
^{15}N NMR (71 MHz, DMSO-d6): 245.8 (N1),169.8 (N3).

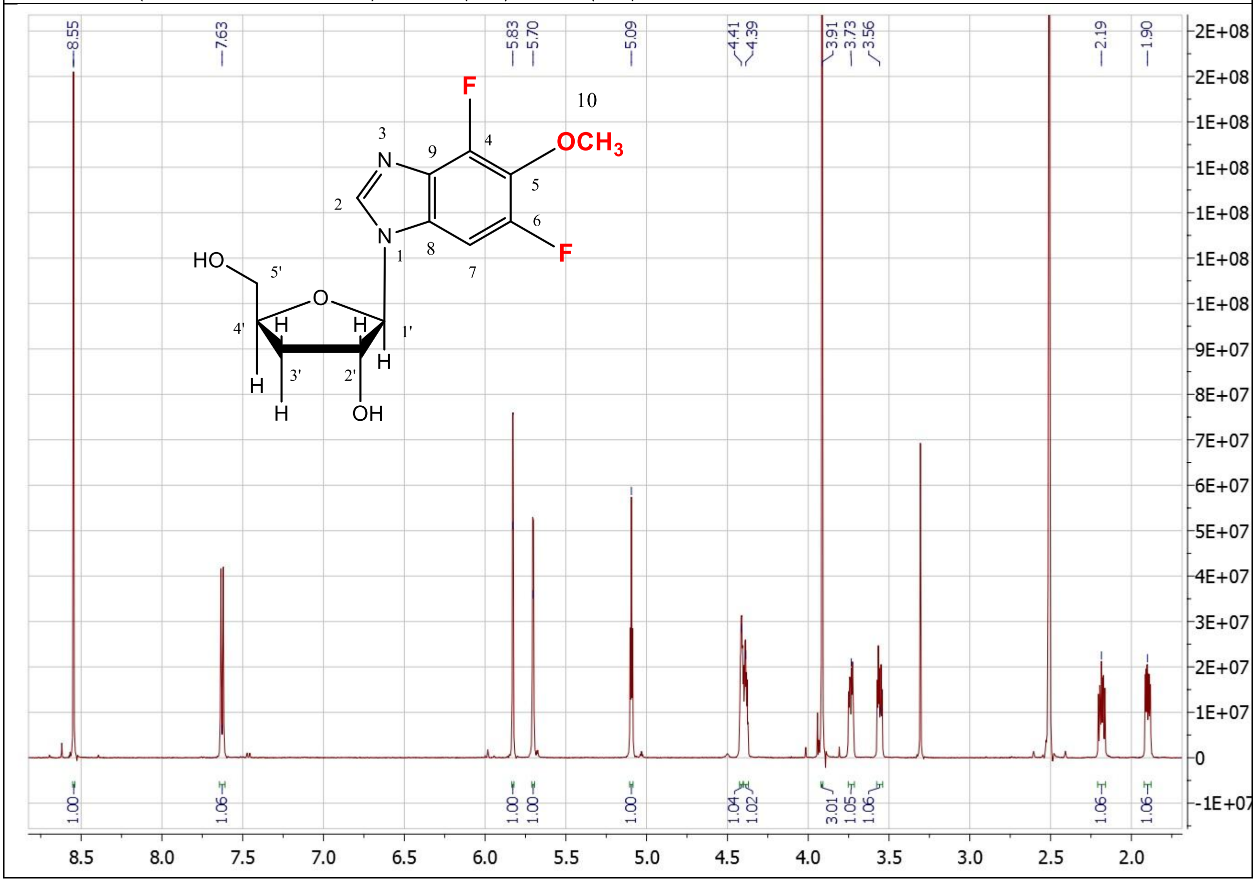

Figure SI-35. The ^{1}H NMR spectrum of 4,6-difluoro-5-metoxy-1-(β-D-3'-deoxyribofuranosyl)benzimidazole **13.**

^{1}H NMR (700 MHz, DMSO-d6): 8.55, (s, 1H, H-2), 7.63 (d, *J* = 10.4, 1H, H-7), 5.83 (d, *J* = 2.4, 1H, H-1'), 5.70 (d, *J* = 3.6, 1H, 2'-OH), 5.13 – 5.07 (m, 1H, 5'-OH), 4.43 – 4.40 (m, 1H, 2'-OH), 4.40 – 4.37 (m, 1H, H-4'), 3.91 (s, 3H, H-10), 3.75 - 3.72 (m, 1H, H-5'), 3.58 – 3.54 (m, 1H, H-5'), 2.22 - 2.15 (m, 1H, H-3'), 1.90 (ddd, *J* = 2.9; 6.3; 9.5, 1H, H-3').

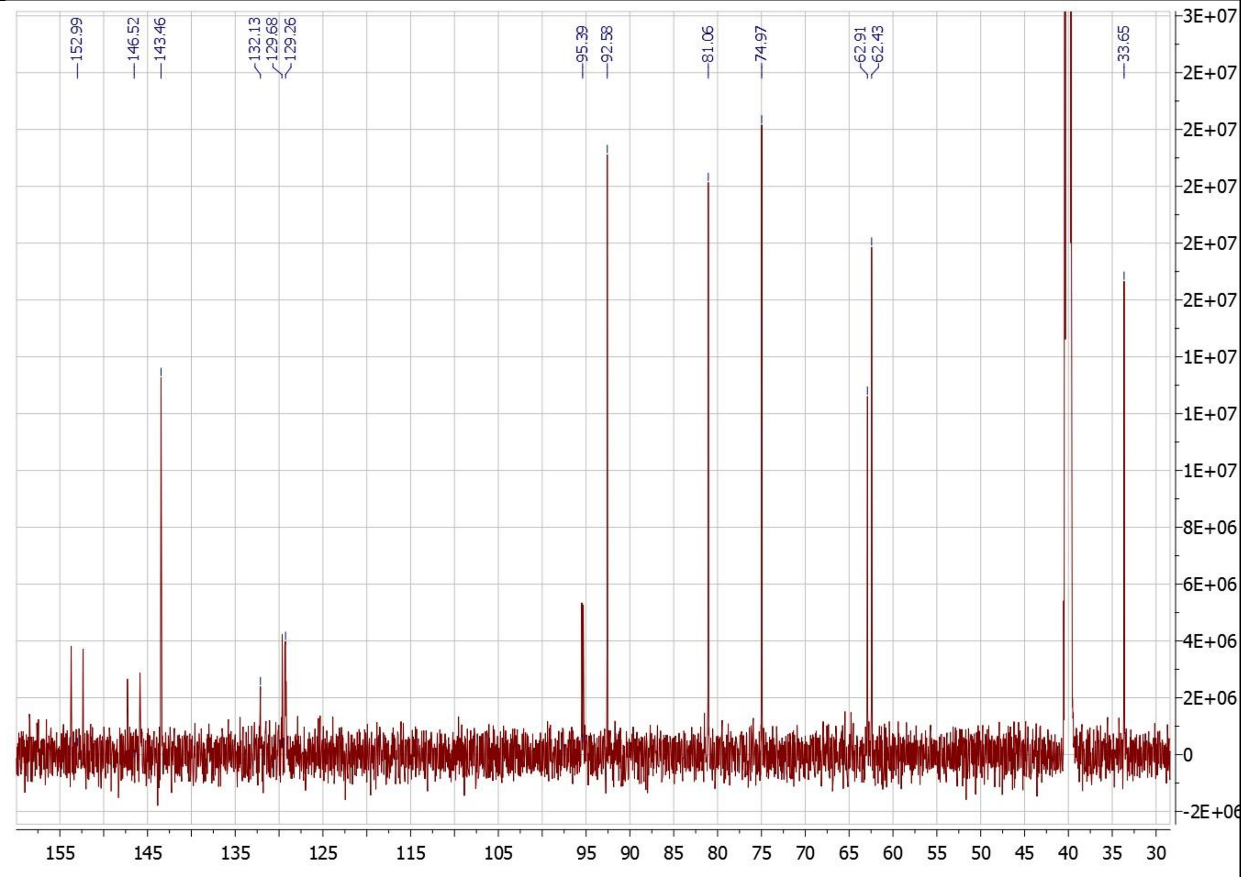

Figure SI-36. The ^{13}C NMR spectrum of 4,6-difluoro-5-metoxy-1-(β-D-3'-deoxyribofuranosyl)benzimidazole **13.**

^{13}C NMR (176 MHz, DMSO-d6): 152.99 (dd, *J* = 240.0; 2.6, C6), 146.52 (dd, *J* = 254.1; 6.7, C4), 143.46 (s, C2), 132.28 - 132.07 (m, C5), 129.68 (d, *J* = 14.3, C9), 129.26 (dd, *J* = 15.1; 10.0, C8), 95.39 (dd, *J* = 25.8; 3.1, C7), 92.58 (s, C1'), 81.06 (s, C4'), 74.97 (s, C2'), 62.91 (s, C10), 62.43 (s, C5'), 33.65 (s, C3').

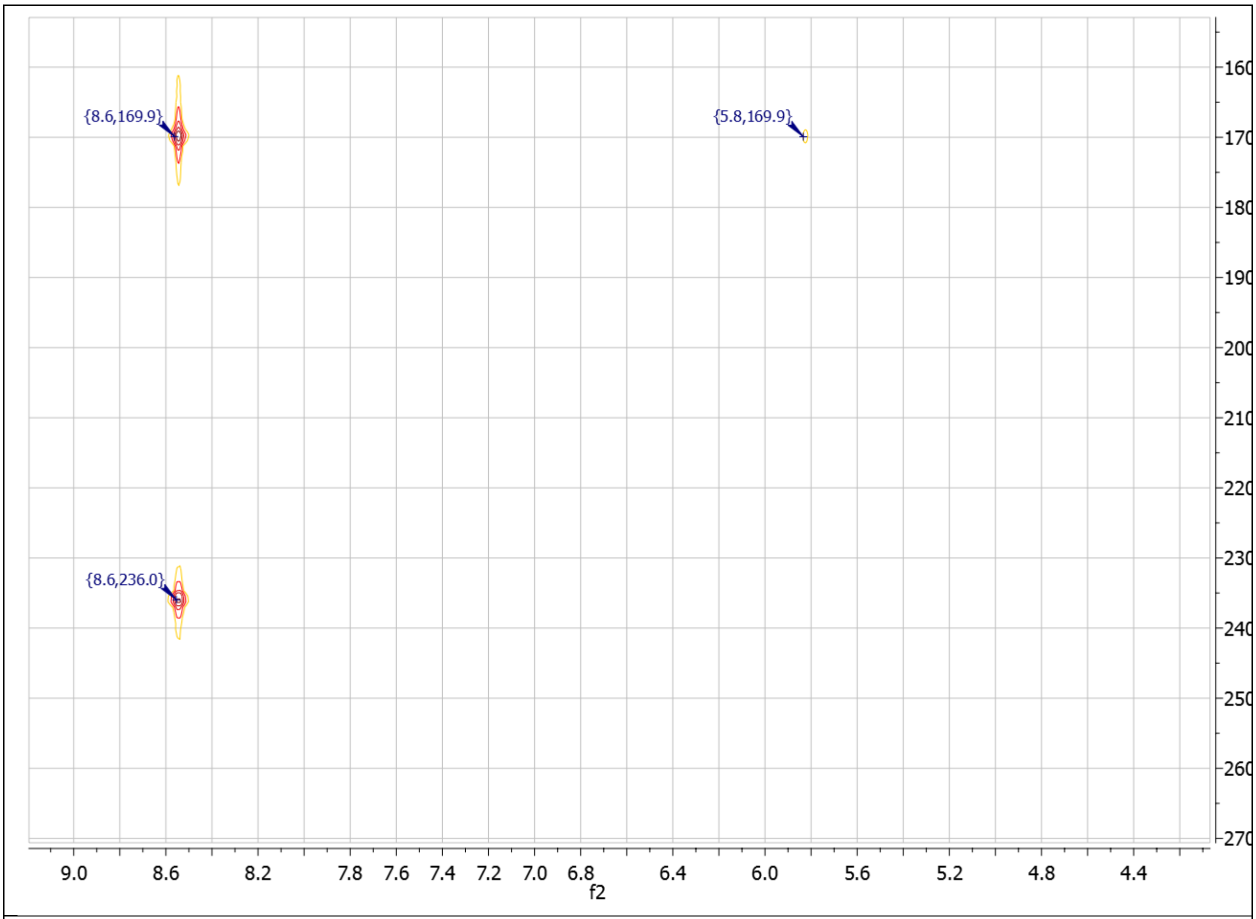

Figure SI-37. The ^{1}H-^{15}N HMBC NMR spectrum of 4,6-difluoro-5-metoxy-1-(β-D-3'-deoxyribofuranosyl)benzimidazole **13.**

^{15}N NMR (71 MHz, DMSO-d6): 236.0 (N3),169.9 (N1).

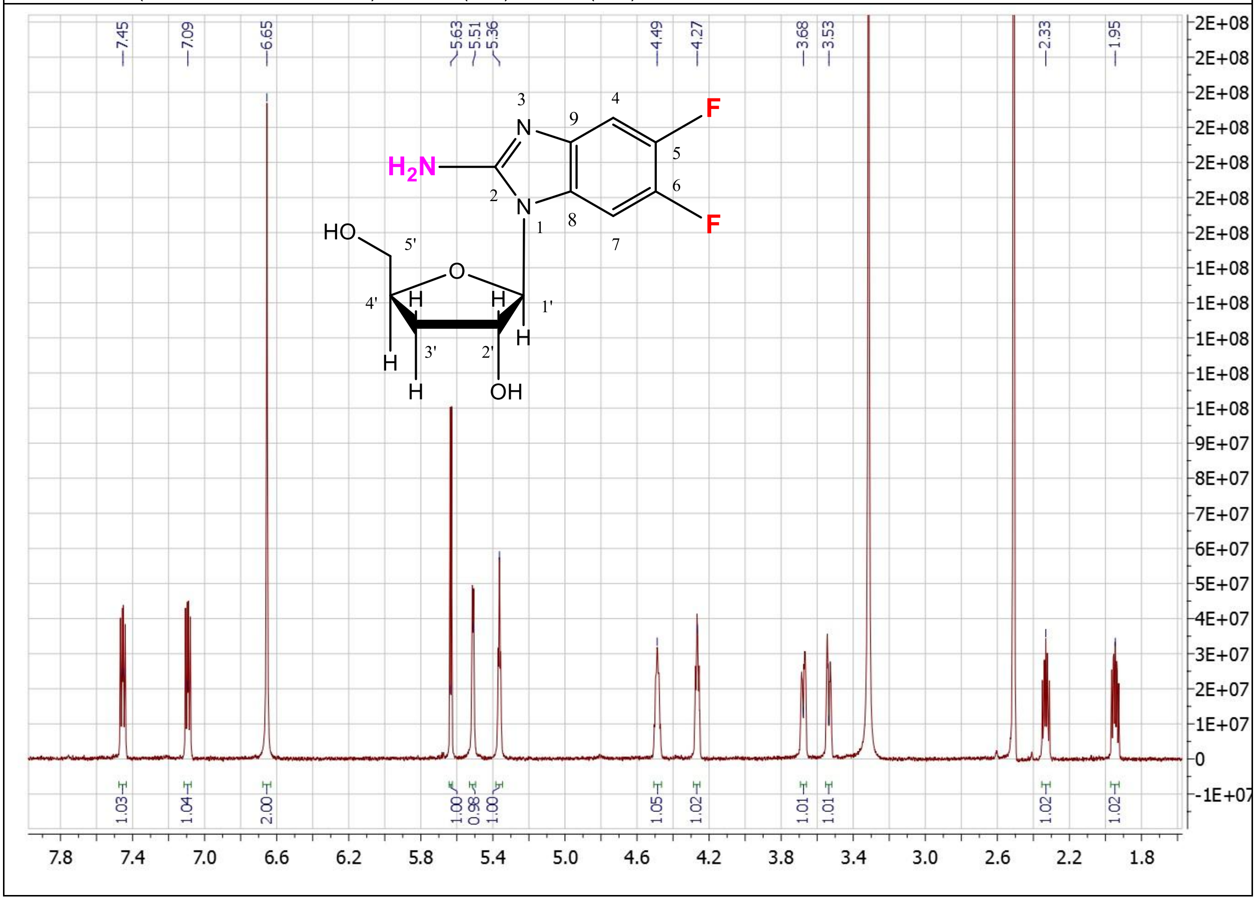

Figure SI-38. The ^{1}H NMR spectrum of 2-amino-5,6-difluoro-1-(β-D-3'-deoxyribofuranosyl)benzimidazole **16.**

^{1}H NMR (700 MHz, DMSO-d6): 7.45 (dd, *J* = 11.2; 7.5, 1H, H-4), 7.09 (dd, *J* = 11.3; 7.4, 1H, H-7), 6.65 (s, 2H, H-10), 5.63 (d, *J* = 5.5, 1H, H-1'), 5.51 (d, *J* = 4.8, 1H, 2'-OH), 5.30 – 5.38 (m, 1H, 5'-OH), 4.50 – 4.45 (m, 1H, H-2'), 4.28 – 4.24 (m, 1H, H-4'), 3.69 - 3.65 (m, 1H, H-5'), 3.55 – 3.50 (m, 1H, H-5'), 2.33 (ddd, *J* = 13.1; 8.0; 6.3, 1H, H-3'), 1.95 (ddd, *J* = 13.1; 8.0; 6.3, 1H, H-3').

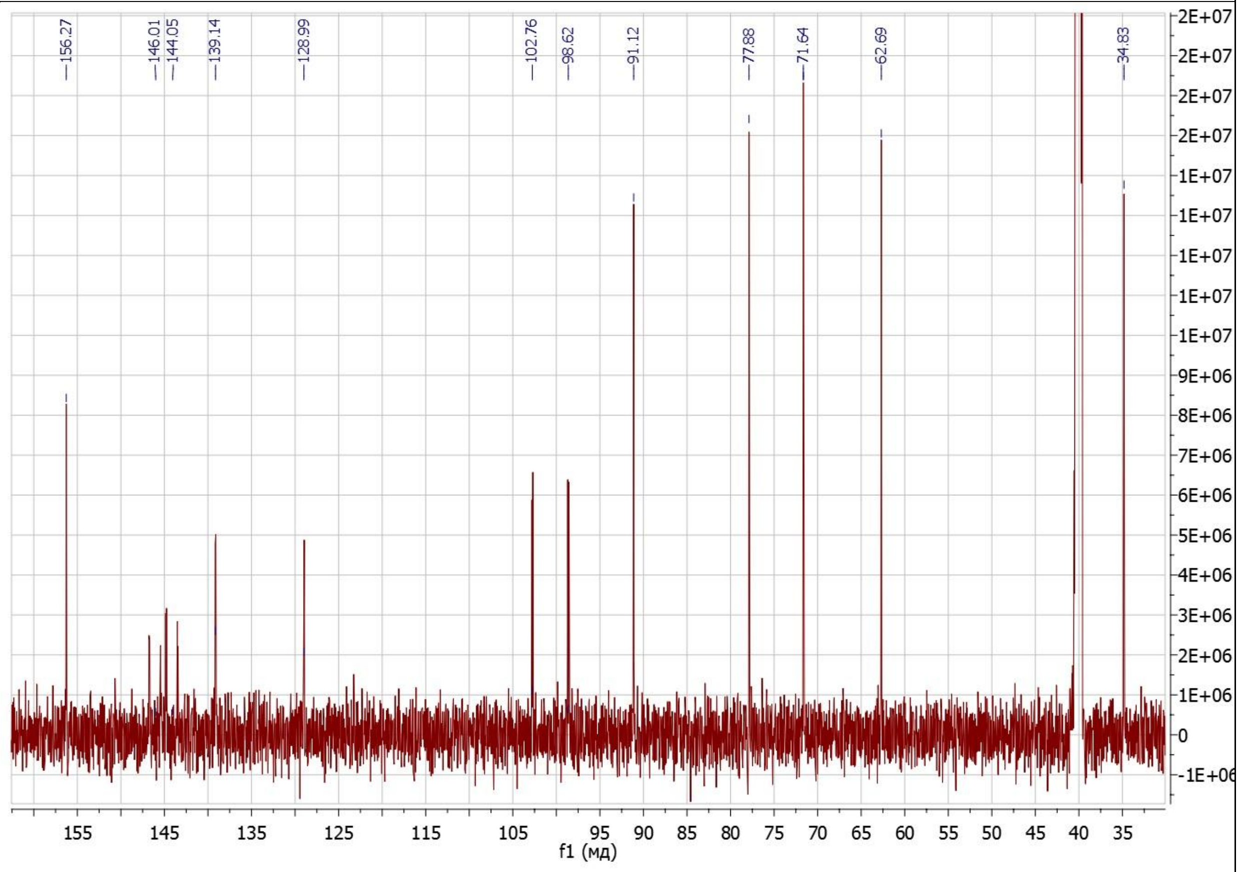

Figure SI-39. The ^{13}C NMR spectrum of 2-amino-5,6-difluoro-1-(β-D-3'-deoxyribofuranosyl)benzimidazole **16.**

^{13}C NMR (176 MHz, DMSO-d6): 156.27 (s, C2), 146.01 (dd, *J* = 234.4; 14.5, C5), 144.05 (dd, *J* = 232.1; 14.5, C6), 139.14 (d, *J* = 10.6 C9), 128.99 (d, *J* = 12.0, C8), 102.76 (d, *J* = 20.1, C7), 98.62 (d, *J* = 23.5, C4), 91.12 (s, C1'), 77.88 (s, C4'), 71.64 (s, C2'), 62.69 (s, C5'), 34.83 (s, C3').

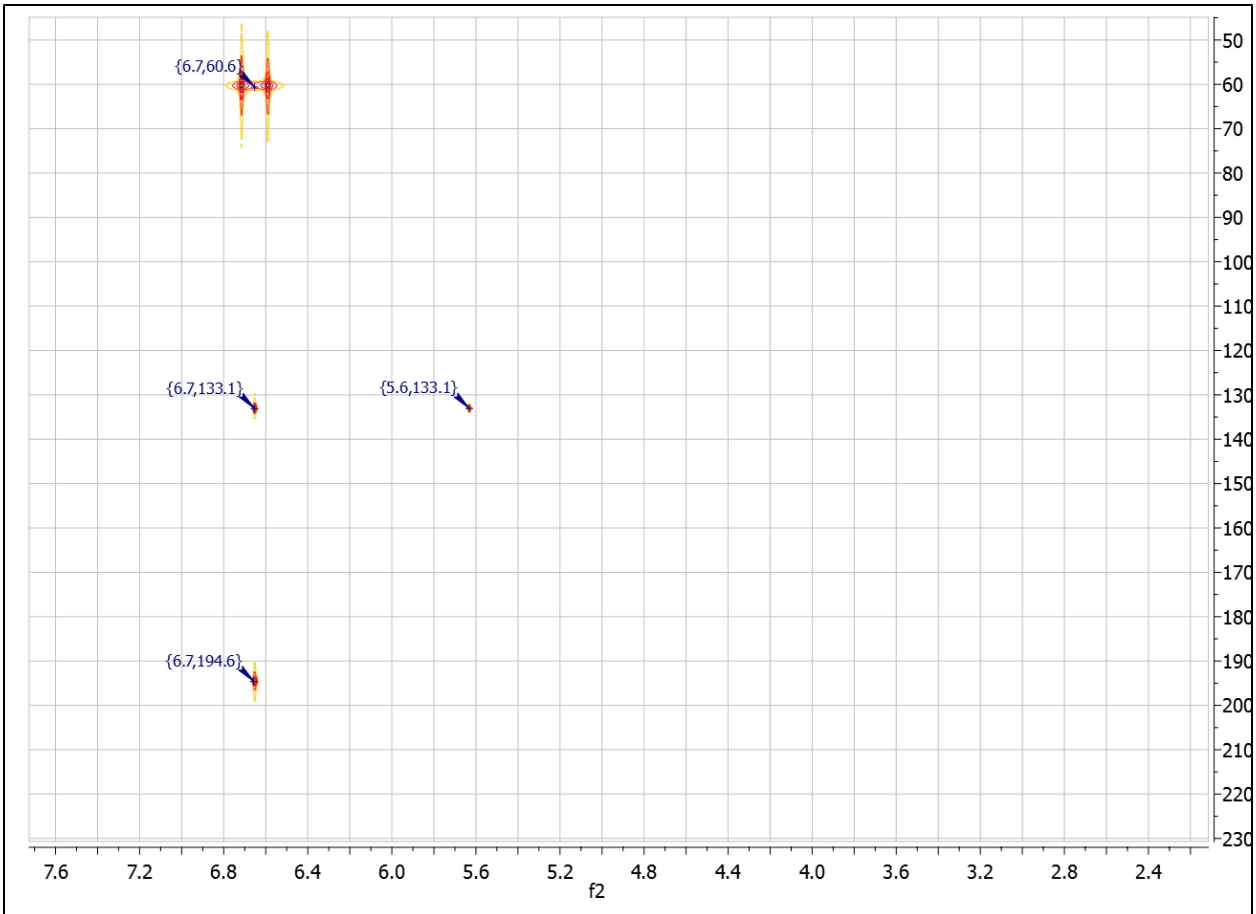

Figure SI-40. The ^{1}H-^{15}N HMBC NMR spectrum of 2-amino-5,6-difluoro-1-(β-D-3'-deoxyribofuranosyl)benzimidazole **16.**

^{15}N NMR (71 MHz, DMSO-d6): 194.6 (N3), 133.1 (N1), 60.6 (N10).

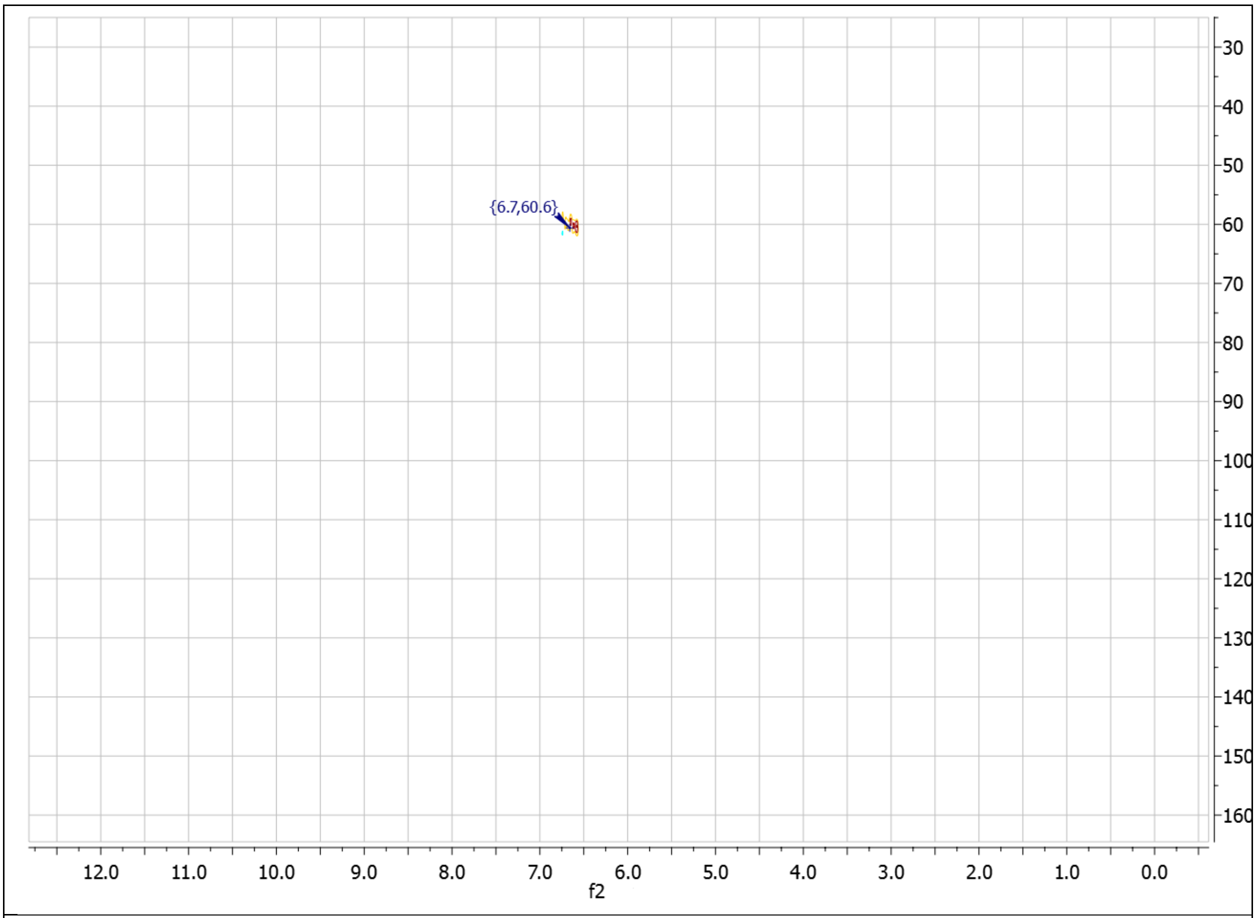

Figure SI-41. The ^{1}H-^{15}N HSQC NMR spectrum of 2-amino-5,6-difluoro-1-(β-D-3'-deoxyribofuranosyl)benzimidazole **16.**

Table SI-1 – *In vitro* Antiviral Activity of Compounds **1 – 13**, **16** against HSV-1.

Compound	MTC [a], µM	CD_{50} [b], µM	HSV-1/L_2			HSV-1/ L_2/R^{ACV}		
			IC_{50} [c]	IC_{95}	SI [d]	IC_{50} [c]	IC_{95}	SI [d]
			MOI 0.01 PFU/ml [f]					
Ribavirin (control)	4095.00	n.i.	511.88	2047.0	>8	511.88	2047.0	>8
**1**	4235.49	6313.85±167.47	1058.87	4235.49	5.96	1058.87	4235.49	5.96
**9**	2505.13	>5019.25	626.28	2505.13	>8	1252.56	5019.25	>4
**2**	1623.06	2433.81±37.72	202.88	1623.06	12.32	405.77	1623.06	6.16
**10**	1970.86	2901.81±60.28	246.36	985.43	12	1478.15	≥1970.86	2
**3**	811.53	2489.32±92.46	811.53	1623.06	3.07	811.53	1623.06	3.07
**11a**	1003.68	>2007.37	250.92	1003.68	>8	<2007.37	2007.37	>1
**4**	1453.32	2179.98±27.03	363.33	1453.32	6.00	363.33	1453.32	6.00
**12a**	1999.65	≥3999.31	249.96	999.83	16	493.91	1999.65	8
**5**	1358.40	2909.48±123.18	1018.80	2716.80	2.86	1358.40	2716.80	2.14
**13**	1665.22	>3330.45	3000	3330.45	>3	3000	3330.45	>2
**6**	939.43	1422.89±18.04	469.71	939.43	3.03	704.57	939.43	2.02

**7**	1380.68	2165.79±33.74	172.59	690.34	12.55	172.59	690.34	12.55
**8**	739.43	1659.39±60.16	369.71	739.43	4.49	369.71	739.43	4.49
**16**	1991.35	3315.58±141.99	995.68	>1991.35	3	995.68	>1991.35	3

The results of three independent experiments are given.
Number of stained cells in control – 4,95±0,07.
[a] MTC – maximal tolerable concentration – highest concentration of a compound that does not cause death of Vero E6 cells;
[b] CD_{50} - 50% cytotoxic concentration of compound, required to reduce the viability of Vero E6 cells by 50%;
[c] IC_{50} и IC_{95} - concentrations of compounds inhibiting CPE development by 50% and almost completely;
[d] SI – selectivity index (CD_{50}/IC_{50});
[f] MOI - multiplicity of infection, PFU – plaque-forming unit;
«>» - no effect is achieved when the compound is used at the maximum concentration tested;
«n.i.» - have not been investigated.
